# Synthesis of Combretastatin A-4 and 3′-Aminocombretastatin A-4 derivatives with Aminoacid Containing Pendants and Study of their Interaction with Tubulin and as Downregulators of the VEGF, hTERT and c-Myc Gene Expression

**DOI:** 10.3390/molecules25030660

**Published:** 2020-02-04

**Authors:** Raül Agut, Eva Falomir, Juan Murga, Celia Martín-Beltrán, Raquel Gil-Edo, Alberto Pla, Miguel Carda, J. Alberto Marco

**Affiliations:** 1 Departamento de Química Inorgánica y Orgánica, Universidad Jaume I, E-12071 Castellón, Spain; ragut@uji.es (R.A.); jmurga@uji.es (J.M.); beltranc@uji.es (C.M.-B.); ragil@uji.es (R.G.-E.); apla@uji.es (A.P.); 2 Departamento de Química Orgánica, Universidad de Valencia, E-46100 Valencia, Spain; alberto.marco@uv.es

**Keywords:** combretastatin A-4, 3′-aminocombretastatin A-4, cytotoxicity, tubulin, microtubules, cell cycle, *VEGF*, *hTERT*, c-*Myc*

## Abstract

Natural product combretastatin A-4 (CA-4) and its nitrogenated analogue 3′-aminocombretastatin A-4 (AmCA-4) have shown promising antitumor activities. In this study, a range of CA-4 and AmCA-4 derivatives containing amino acid pendants have been synthesized in order to compare their biological actions with those of their parent compounds. Thus, inhibition of cell proliferation on tumor cell lines HT-29, MCF-7 and A-549, as well as on the nontumor cell line HEK-273; in vitro tubulin polymerization; mitotic cell arrest; action on the microtubule cell network and inhibition of *VEGF*, *hTERT,* and *c-Myc* genes have been evaluated. Some AmCA-4 derivatives bearing L-amino acids exhibited inhibition of cell proliferation at low nanomolar levels exceeding the values shown by AmCA-4. Furthermore, while CA-4 and AmCA-4 derivatives do not show significant effects on the in vitro tubulin polymerization and cell cycle arrest, some selected CA-4 and AmCA-4 derivatives are able to cause total depolymerization of the microtubule network on A-549 cells. The best results were obtained in the inhibition of gene expression, particularly on the *VEGF* gene, in which some AmCA-4 derivatives greatly exceeded the inhibition values achieved by the parent compound.

## 1. Introduction

**Figure 1:**
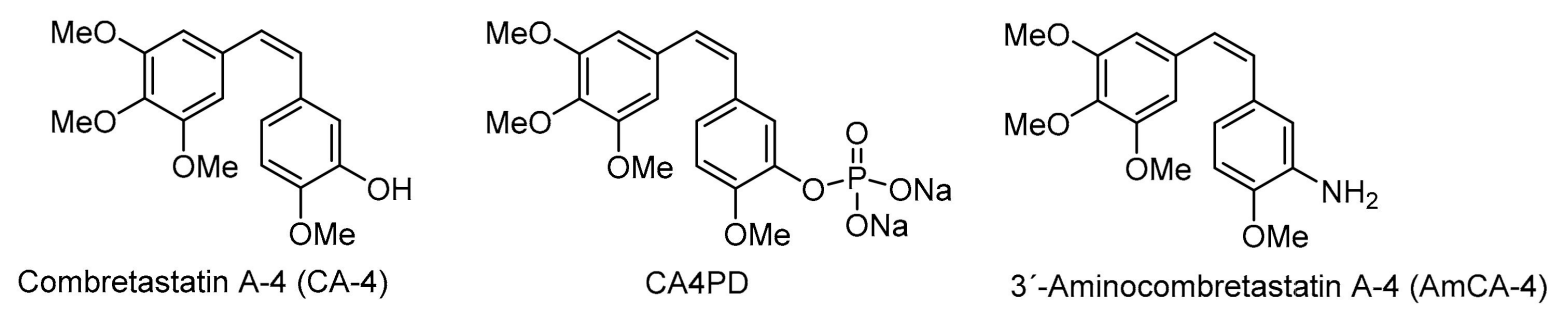
Structures of combretastatin A-4, combretastatin A-4 disodium phosphate and 3′-aminocombretastatin A-4.

Cancer is a disease characterized by the growth and spread by the body of aberrant cells. Cancer, along with cardiovascular diseases, are the two leading causes of mortality in developed countries. The ageing of the populations in these countries cause an increase in cancer mortality so that there is considerable interest in the discovery of new anticancer agents. A range of natural products or direct derivatives are used in the treatment of cancer, and over the last 35 years, more than half of the new compounds indicated for the treatment of cancer are natural products or derivatives thereof [1]. Combretastatins are a class of natural phenolic stilbene compounds that have been isolated from the African willow tree *Combretum caffrum*. The most potent member of these natural stilbenes is combretastatin A-4 (CA-4, see Figure 1), whose antimitotic action is based on the inhibition of tubulin polymerization. CA-4 shows strong cytotoxicity against a variety of cancer cells, including multidrug resistant cancer cell lines [2,3,4]. In addition, CA-4 has also been demonstrated to exert effects in the proliferation of endothelial cells [5] and to act as a vascular-disrupting agent [6].

Recently, Steinmetz and colleagues have confirmed, by resolution of the crystal structure of a tubulin-combretastatin A-4 complex, that the binding site of CA-4 to β-tubulin is the colchicine site [7]. Combretastatin A-4 disodium phosphate (CA4DP, see Figure 1) is a water-soluble prodrug of CA-4 that shows potent antivascular and antitumor effects in a wide variety of tumor cell lines [8]. 3′-Aminocombretastatin (AmCA-4, see Figure 1) is a non-natural combretastatin analogue that also shows strong cytotoxicity, inhibition of tubulin polymerization and antivascular activity [9,10,11].

The formation of new blood vessels from pre-existing vasculature is known as angiogenesis. Tumoral angiogenesis causes tumor growth and dissemination of tumor cells, which ends up as metastasis [12]. There are several “on” and “off” switches that regulate the angiogenesis process. Vascular endothelial growth factor (VEGF) is an inducer of angiogenesis and promotes endothelial cell survival, proliferation and migration. Overexpression in the production of VEGF has been reported to occur in various types of tumors [13,14]; therefore, it is not surprising that VEGF and angiogenesis process have become targets in cancer therapy [15].

Telomerase is a reverse transcriptase enzyme that carries a short RNA molecule used as a template to elongate telomeres. Telomerase is inactive in most somatic cells but is active in normal stem cells and most cancer cells [16]. Telomerase does not incite the oncogenic process, but its action is needed for the growth and development of many cancers. Since telomerase is not expressed in most normal human cells, this has led to the development of targeted telomerase anticancer agents [17]. The reverse transcriptase subunit of human telomerase (hTERT) catalyzes the nucleotide polymerization process. Human telomerase is regulated during development and differentiation, mainly through transcriptional control of the *hTER*T gene, the expression of which is restricted to cells that exhibit telomerase activity. For the expression of the *hTERT* gene, several transcriptional factors such as c-Myc, a proto-oncoprotein [18], have been found to play an important role through upregulation of the mRNA encoding the hTERT protein subunit [19,20]. Moreover, deregulation of c-Myc has been described as an upregulator of VEGF and hTERT gene expression and as one of the major factors responsible for increased tumor malignancy [21].

During the past few years, we have been investigating a range of analogues of natural products for their potential values in anticancer therapy as multitarget agents [22,23]. We have published several reports on the biological properties of combretastatin and aminocombretastatin A-4 derivatives [24,25,26,27,28,29]. In addition to their cytotoxicity, we have also investigated their ability to inhibit the expression of certain genes related to the angiogenesis process and the telomerase activation, such as c-*Myc*, *VEGF* and *hTERT* genes, as well as their actions as vascular disrupting agents. Therefore, based on the aspects commented above and in continuation of our research on novel natural product analogues with multitarget actions in cancer therapy, we wanted to ascertain whether the introduction of amino acid subunities in combretastatin A-4 and 3′-aminocombretastatin A-4 might give rise to noticeable changes in the biological activity. For these reasons, we have conceived the synthesis and biological evaluation of a range of combretastatin and 3′-aminocombretastatin A-4 derivatives having amino acid pendants.

## 2. Results

### 2.1. Chemistry

**Scheme 1:**
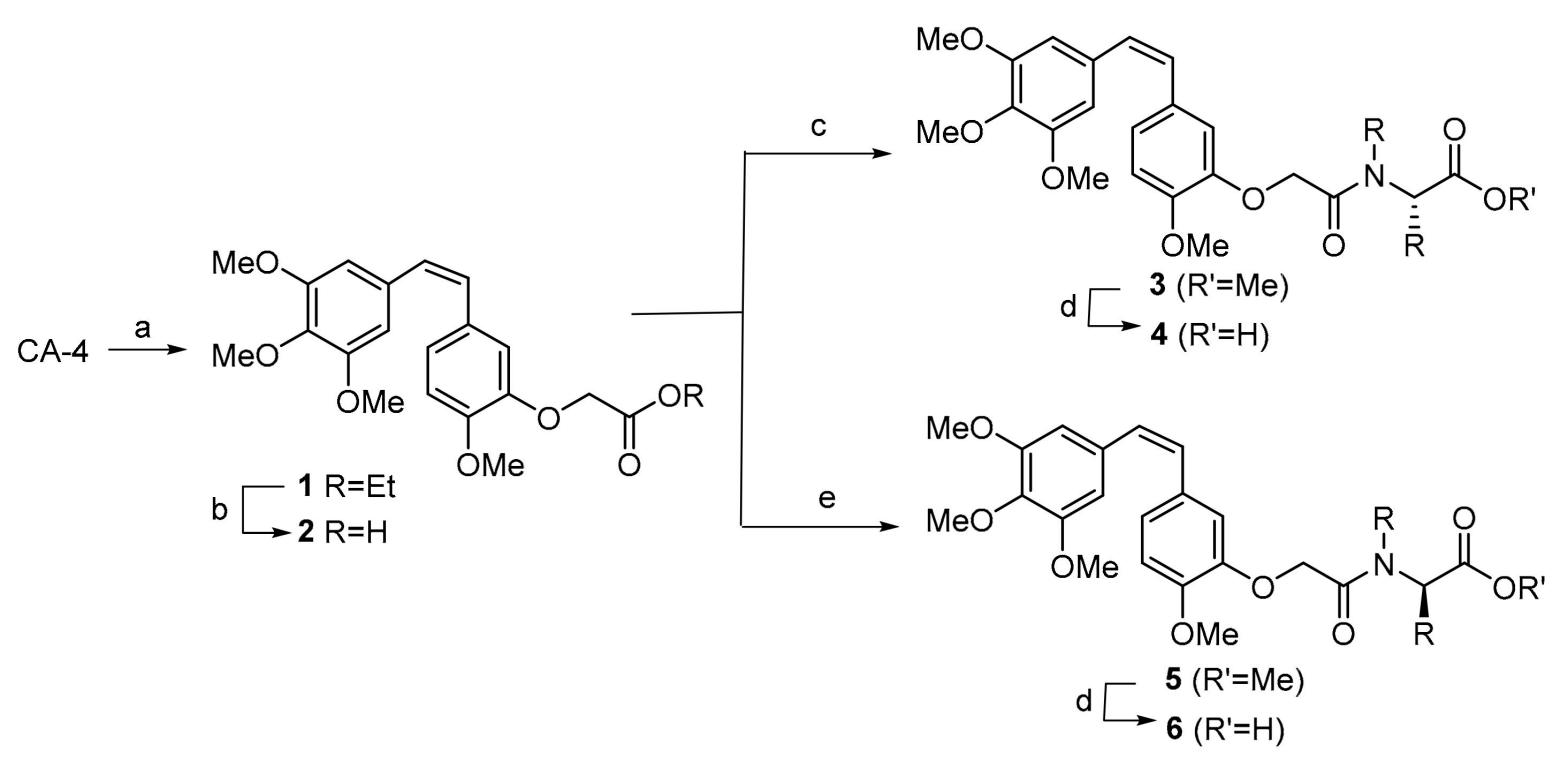
Synthesis of combretastatin A-4 (CA-4) derivatives. Reagents and conditions: (**a**) K_2_CO_3_ and DMF, RT 1 h., then BrCH_2_COEt, RT 24 h, 94%. (**b**) NaOH and EtOH, RT 1 h, 91%. (**c**) l-amino ester, EDCI·HCl, DMAP and DMF, 0 °C, 2 h, then RT 2 h. (**d**) LiOH and MeOH/H_2_O, RT 1 h. (**e**) d-amino ester, EDCI·HCl, DMAP and DMF, 0 °C, 2 h, then RT 2 h.

In order to obtain CA-4 derivatives carrying amino acids, we have used an acetic acid linkage to connect the CA-4 and the amino acid fragments, as indicated in Scheme 1. Thus, CA-4 was converted into compound **1** by reaction with ethyl bromoacetate in the presence of K_2_CO_3_. Saponification of **1** led to acid **2,** which was coupled with a range of methyl l- and d-amino esters in the presence of EDCI·HCl and DMAP [30] to afford compounds of general structures **3** and **5**, respectively. Saponification of these compounds afforded combretastatin A-4/amino acid conjugates **4** and **6**.

**Figure 2:**
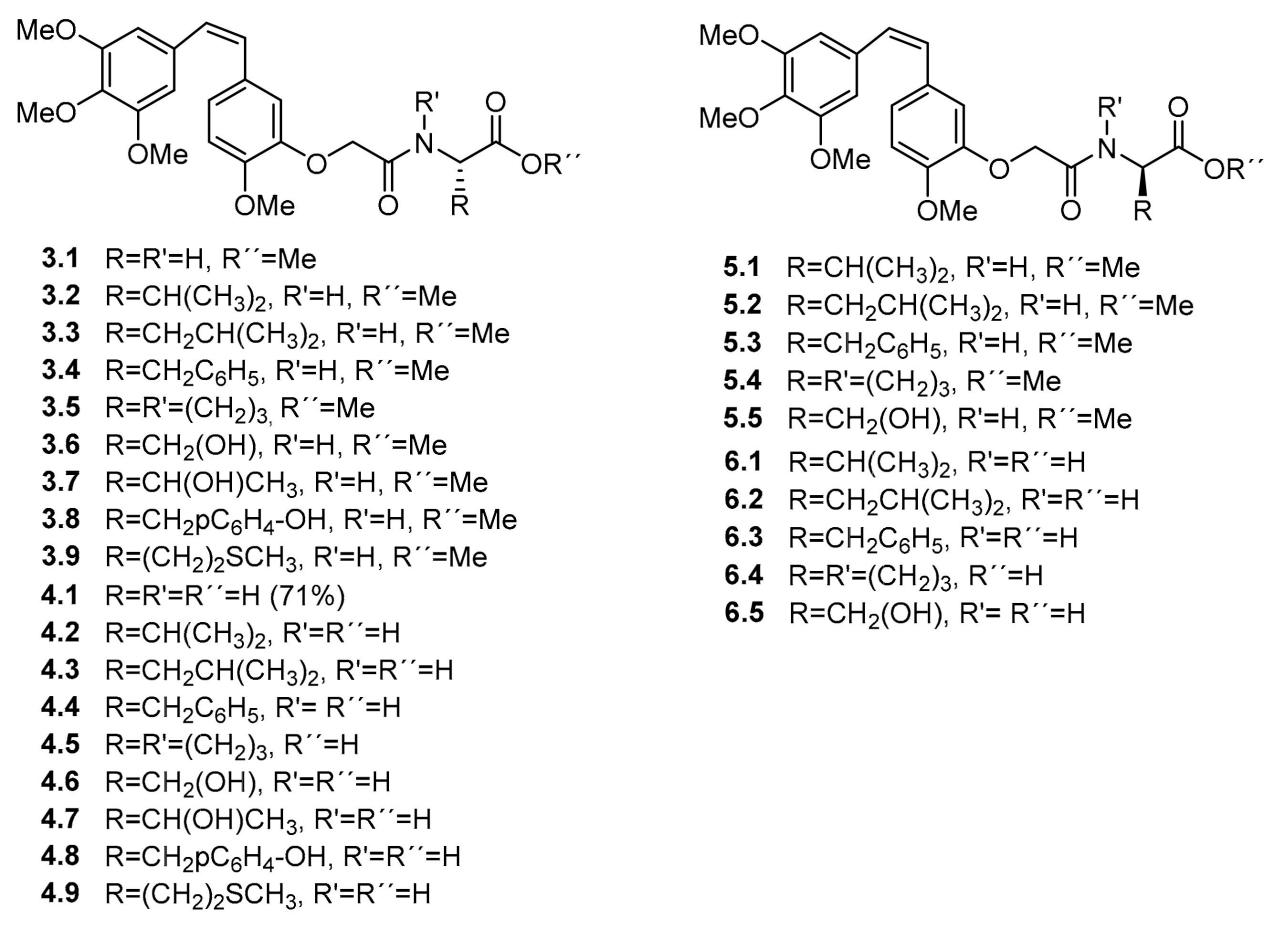
Structures of CA-4 derivatives.

The structures of the CA-4 derivatives, as well as the esters from which they are derived, are indicated in Figure 2 (for yields and spectral data, see Supplementary Materials).

**Scheme 2:**
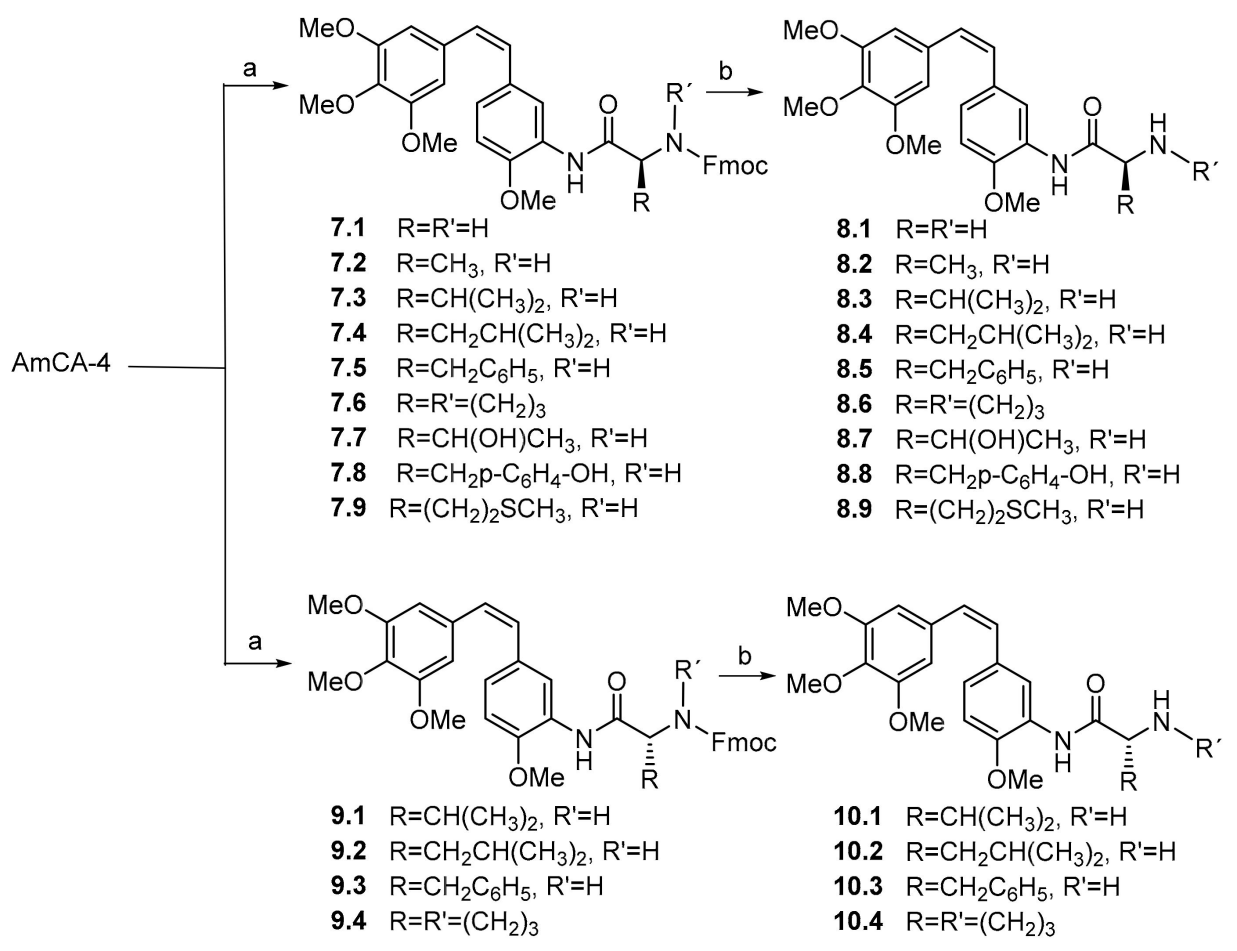
Synthesis of 3′-aminocombretastatin A-4 (AmCA-4) derivatives. Reagents and conditions: (a) *N*-Fmoc-l-amino acid or *N*-Fmoc-d-amino acid, DCC, HOBt and DMF, RT 16 h. (b) KF, 18-crown-6 and DMF, RT 4 h.

As regards the AmCA-4 derivatives carrying amino acids, they were obtained by means of direct coupling with *N*-fluorenylmethoxycarbonyl (*N*-Fmoc)-protected amino acids [31], followed by *N*-Fmoc deprotection [32] (see Scheme 2; for yields and spectral data, see Supplementary Materials).

### 2.2. Inhibition of Cell Proliferation

The ability of the synthetic compounds to inhibit cell proliferation was established by means of their IC_50_ values towards the tumor cell lines HT-29 (human colon adenocarcinoma), MCF-7 (breast adenocarcinoma) and A-549 (human pulmonary adenocarcinoma), as well as towards the nontumor cell line HEK-293 (human embryonic kidney cells). Results for compounds derived from CA-4 are presented in Table 1, along with the calculated selectivity indexes SI_A_ (for HT-29 cell line), SI_B_ (for MCF-7 cell line) and SI_c_ (for A-549 cell line), obtained by dividing the IC_50_ values of the nontumor cell line (HEK-293) by those of the corresponding tumor cell line. The higher the SI index, the higher the therapeutic safety margin. By comparison, the IC_50_ values of CA-4 are also indicated in Table 1.

All derivatives indicated in Table 1 show an antiproliferative activity in the micromolar range, with values similar to those of the natural product CA-4. For the HT-29 cell line, the most active compounds are **1** (7 μM); **3.6** (l-Ser-OMe, 9 μM) and **4.1** (Gly, 5 μM), although their selectivity indexes are lower than 1. On the contrary, compounds **4.5** (l-Pro), **4.9** (l-Met), **6.2** (d-Leu), **6.3** (d-Phe), **6.24** (d-Pro) and **6.25** (d-Ser) show SI values greater than 1, although they do not reach the high SI values displayed by CA-4. In the MCF-7 cell line, compounds **3.6** (l-Ser-OMe, 7 μM); **4.1** (Gly, 6 μM); **4.2** (l-Val, 7 μM) and **5.5** (d-Ser-OMe, 7 μM) are the most active, showing **3.6** and **5.5** selectivity indexes above 1. In the A-549 cell line, IC_50_ values are, in general, comparable to those of the other cell lines, with compounds **1**, **3.3** (l-Leu-OMe), **3.4** (l-Phe-OMe), **3.9** (l-Met-OMe) and **5.1** (d-Val-OMe) exhibiting IC_50_ values less than 10 μM. In this cell line, compounds **3.3** (l-Leu-OMe) and **4.8** (l-Tyr) exhibit SI values greater than 1.

IC_50_ values and SI indexes for AmCA-4 derivatives are presented in Table 2. AmCA-4 derivatives show important differences in antiproliferative activity, which depends on the configuration of the amino acid.

Those derivatives that incorporate amino acids of the l series exhibit IC_50_ values in the nanomolar range of the same order of magnitude, in general, as AmCA-4 and are much more active than the natural product CA-4. Compounds **7.2** (Fmoc- l-Ala), **8.2** (l-Ala), **8.4** (l-Leu), **8.5** (l-Phe), **8.8** (l-Tyr) and **8.9** (l-Met) show IC_50_ values lower than 5 nM in HT-29, MCF-7 and HEK-293 cell lines. The least active of the L series are those that contain the amino acid proline (**7.6** and **8.6**). As for the selectivity indexes, the highest values in the HT-29 cell line are observed for derivatives **7.2** (Fmoc-l-Ala), **7.7** (Fmoc-l-Thr), **8.2** (l-Ala), **8.6** (l-Pro), **8.7** (l-Thr) and **8.8** (l-Tyr), with values higher than 1; thus, higher than those of AmCA-4 but lower than those of CA-4. For the A-549 cell line, the IC_50_ values are some orders of magnitude higher than those obtained in the other two tumor cell lines, with SI values similar to those of AmCA-4. On the other hand, the derivatives of d series **9.1**–**10.4** exhibit IC_50_ values in the micromolar range; therefore, with activities similar to that of CA-4, or even lower. Only **10.2** (d-Leu) exhibits antiproliferative activity in the high nanomolar range in the A-549 cell line, together with a high SI value (2.7).

### 2.3. Effect on Microtubule Assembly

The effect on the self-assembly of tubulin was determined by measuring the critical concentration (CrC). All derivatives were tested at a concentration of 27.5 μM in a glycerol (GAB) and GTP assembly buffer, to which tubulin was added at a concentration of 25 μM. Table 3 shows the results achieved with CA-4 derivatives and compares them with those obtained in the absence of ligand (control) and in the presence of CA-4 as a reference compound.

Unexpectedly, Table 3 shows that, while CA-4 is a potent microtubule destabilizer, most of the synthetic CA-4 derivatives do not exhibit significant differences in effects, as compared with the control. The highest CrC values were obtained by compounds **1**, **3.4** (l-Phe-OMe), **3.6** (l-Ser-OMe), **3.8** (l-Tyr-OMe), **5.1** (d-Val-OMe), **5.2** (d-Leu-OMe) and **5.3** (d-Phe-OMe) (values around 13 μM) very far from the CA-4 value, which is 21.5 μM. In addition, the time needed to start tubulin polymerization is similar to that of the control. Thus, it can be concluded that these CA-4 derivatives do not inhibit in vitro tubulin polymerization.

Table 4 shows the critical concentration values achieved by AmCA-4 derivatives and compares them with those obtained in the absence of ligand (control) and in the presence of CA-4 and AmCA-4 as reference compounds. Most of the AmCA-4 derivatives do not show CrC values different from the control value. It should be noted that compounds **7.1** (Fmoc-Gly), **7.7** (Fmoc-l-Thr), **8.1** (Gly), **8.2** (l-Ala), **8.7** (l-Thr), **9.4** (Fmoc-d-Pro) and **10.1** (d-Val) present CrC values around 10–14 μM, higher than those shown by the control, although much lower than those exhibited by the reference compounds CA-4 and AmCA-4.

**Figure 3:**
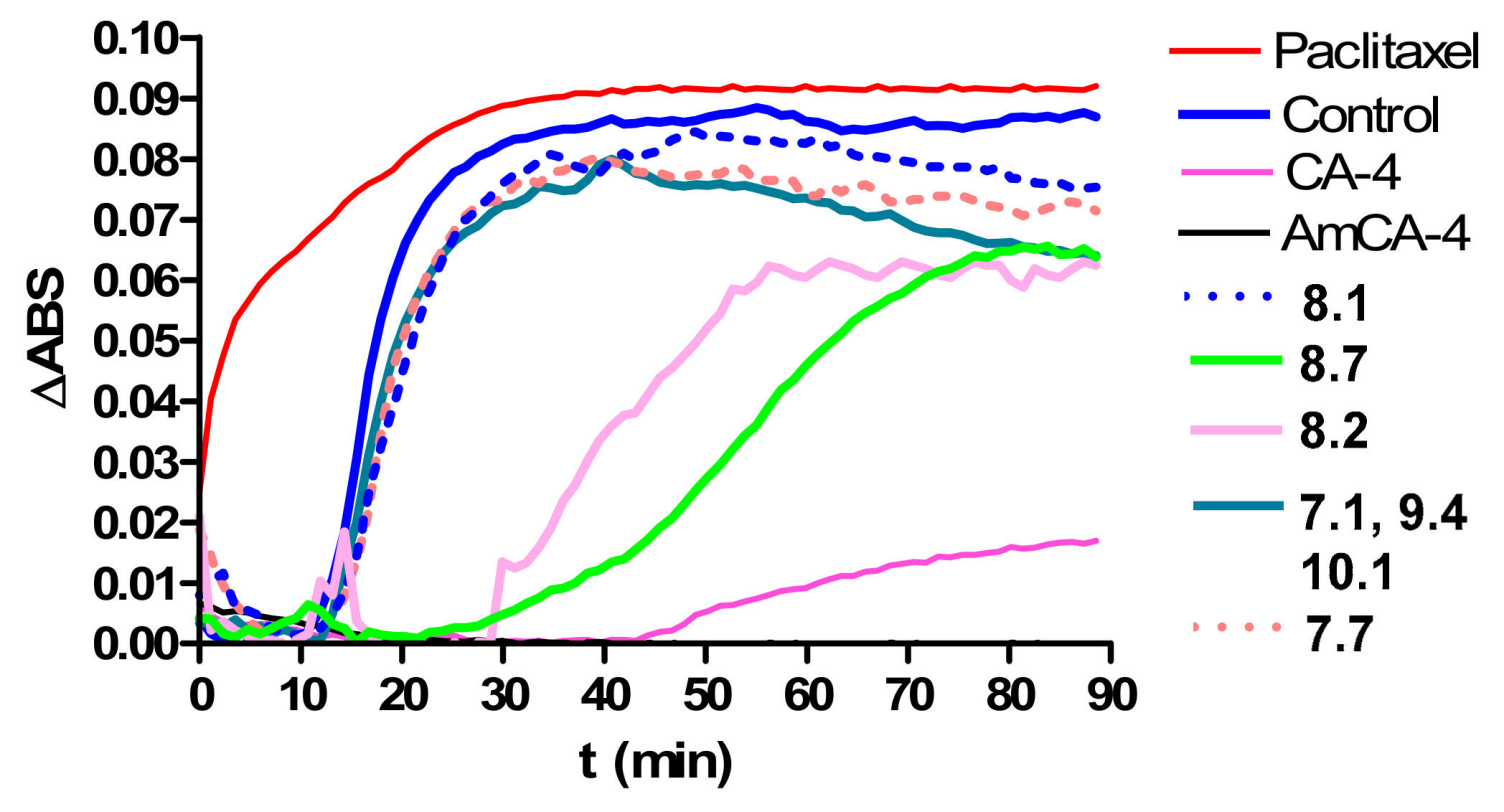
Effects of paclitaxel, CA-4, AmCA-4 and derivatives on the in vitro tubulin polymerization.

The tubulin polymerization process was studied by means of turbidimetry at 350 nm and 37^o^C and is shown in Figure 3 for compounds **7.1** (Fmoc-Gly), **7.7** (Fmoc-l-Thr), **8.1** (Gly), 8.2 (l-Ala), **8.7** (l-Thr), **9.4** (Fmoc-d-Pro) and **10.1** (d-Val), which are those having the highest CrC values. Figure 3 also includes polymerization curves in the presence of CA-4, AmCA-4 and paclitaxel. This test was also performed using paclitaxel, a microtubule stabilizing agent, as a control for the complete formation of microtubules (see Figure 3).

It can be seen in Figure 3 that almost no absorbance is detected in the presence of CA-4 and AmCA-4. In fact, the polymerization curve for AmCA-4 overlaps on the x axis. This is indicative of the inhibition of the polymerization process that these two compounds exert. On the other hand, it can be seen the high absorbance displayed when the polymerization process is carried out in the presence of paclitaxel, a compound that promotes tubulin polymerization. In this case, the plateau phase is reached 30 min after the beginning of the process. CA-4 and AmCA-4 derivatives do not exert significant effects. Only derivatives **8.2** (l-Ala) and **8.7** (l-Thr) are able to delay the polymerization process.

Values for It_50_, which indicate the time required to reach 50% of the polymerization, are given in Table 5.

With control (absence of ligand) and with the majority of derivatives, 50% of the polymerization is reached between 16–20 min. However, with derivative **8.2** (l-Ala), the process suffers a delay of almost 50 min, while for derivative **8.7** (l-Thr), a 65-min delay is measured. These two compounds therefore could be classified as partial inhibitors of the polymerization process.

### 2.4. Effect on the Cell Cycle

The effects of some CA-4 derivatives on the cell cycle were evaluated in A-549 cells. Cell cycle results for CA-4 derivatives are shown in Table 6, as well as the ligand concentrations at which the test was conducted (see Supplementary Information for cell cycle histograms). The derivatives were selected after observing the cell morphology, discarding the compounds that did not show a high concentration of cells stopped in mitosis, which are those that are characterized by having a rounded shape and being slightly detached from the lower base of the plate. It can be seen in Table 6 that the control sample shows very little accumulation of cells in the G2/M phase, while for CA-4, a majority of cells were observed in the G2/M phase (60%). This study was performed using several concentrations for the selected compounds and Table 6 collects the lowest effective concentration for each compound without causing cell death. Compounds **1**, **3.4** (l-Phe-OMe), **3.6** (l-Ser-OMe), **3.7** (l-Thr-OMe), **3.8** (l-Tyr-OMe), **4.2** (l-Val), **4.3** (l-Leu) and **5.5** (d-Pro-OMe) accumulate between 50–70% of cells in the G2/M phase, in some cases improving the results obtained for CA-4 (60%). It should be noted, however, that these compounds exert their actions at concentrations between 1000–1500 times higher than those of CA-4. Effects of **3.2** (l-Val-OMe), **3.3** (l-Leu-OMe), **3.4** (l-Phe-OMe), **3.9** (l-Met-OMe), **5.3** (d-Phe-OMe) and **5.4** (d-Pro-OMe) are similar to CA-4, but only when they were tested at concentrations between 1000-1500 times higher than those of the natural product. In turn, compounds **3.5** (l-Pro-OMe), **4.1** (Gly), **4.5** (l-Pro), **4.9** (l-Met), **6.1** (d-Val), **6.2** (d-Leu), **6.3** (d-Phe), **6.4** (d-Pro) and **6.5** (d-Ser) do not exert any significant effect on the cells.

The effects of some AmCA-4 derivatives on the cell cycle were also evaluated in A-549 cells. The derivatives were selected after observing the cell morphology, and the assay was carried out only in those compounds in which cells stopped in mitosis were detected; that is, cells rounded off and slightly detached from the lower base of the plate. Compound **3.51** (D-Val), which exerted a lower antimitotic effect, was also studied in order to compare its effect with those of the other derivatives. Cell cycle results for selected AmCA-4 derivatives are presented in Table 7, as well as the ligand concentrations at which the test was conducted. It can be seen in Table 7 that for AmCA-4 a majority of cells remain accumulated in the G2/M phase, a situation similar to that previously observed for CA-4. Derivatives **7.1** (Fmoc-Gly), **7.2** (Fmoc-L-Ala), **8.1** (Gly), **8.2** (L-Ala), **8.8** (L-Tyr), **8.9** (L-Met) and **10.1** (D-Val) were capable of accumulating more than 50% of the cells in the G2/M phase, values much higher than those of the control (14%) and similar to those of CA-4 and AmCA-4, although at concentrations higher than those of these compounds. Derivatives **7.9** (Fmoc-L-Met), **8.3** (L-Val) and **10.2** (D-Leu) do not accumulate a high percentage of cells in phase G2/M, and the rest of the derivatives are in an intermediate situation between those commented above.

### 2.5. Effect on the Cellular Microtubule Network

**Figure 4:**
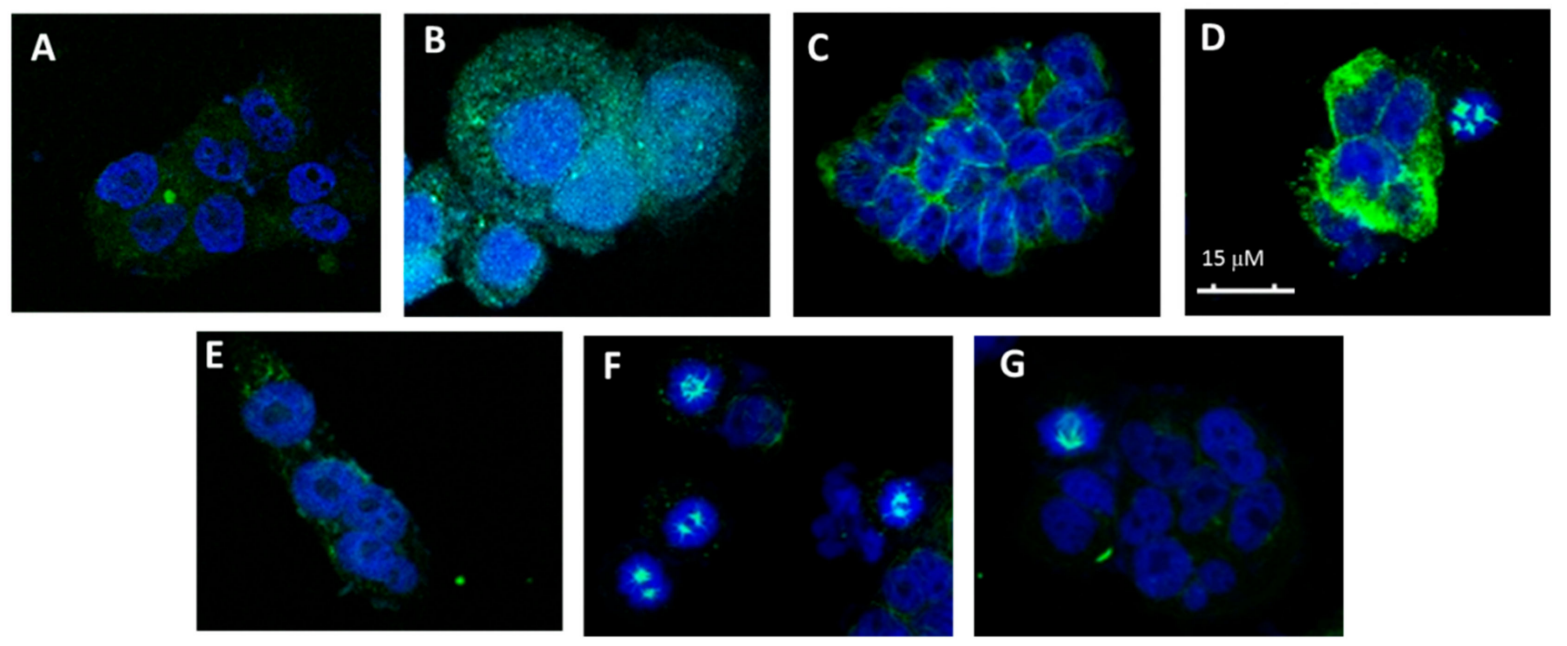
Effects on the microtubule network. (**A**) Cellular microtubules with DMSO, (**B**) 50 nM CA-4, (**C**) 50 μM **1**, (**D**) 75 μM **3.7** (l-Thr-OMe), (**E**) 75 μM **3.8** (l-Tyr-OMe), (**F**) 50 μM **4.2** (l-Val) and (**G**) 75 μM **4.3** (l-Leu).

The effect on the cellular microtubule network of CA-4 derivatives was studied for compounds **1**, **3.7** (l-Thr-OMe), **3.8** (l-Tyr-OMe), **4.2** (l-Val) and **4.3** (l-Leu), which were those accumulating more cells in the G2/M phase. Thus, A-549 cells were incubated for 24 h in the presence of the above compounds and CA-4 at the concentrations indicated in Figure 4. CA-4 (see panel B) greatly depolymerizes microtubules at a concentration of 50 nM. In addition, derivatives **1** (50 μM, panel C); **3.7** (l-Thr-OMe, 75 μM, panel D); **3.8** (l-Tyr-OMe, 75 μM, panel E); **4.4** (l-Val, 50 μM, panel F) and **4.3** (l-Leu, 50 μM, panel G) produce disorganization in the mitotic spindle, although this effect is exerted at much higher concentrations than in the case of the natural product CA-4.

The effect of some derivatives on the cellular microtubule network were also studied on A-549 cells. The selected compounds were **7.1** (Fmoc-Gly) (0.5 μM), **8.1** (Gly) (2 μM), **8.2** (l-Ala) (3 μM) and **10.1** (d-Val) (40 μM), which were those exerting a higher effect on the cell cycle. In addition, other derivatives selected were compound **8.5** (l-Phe) (0.5 μM), as it exhibited a low IC_50_, and compound **10.2** (d-Leu) (0.5 μM), as it did not stand out either for its antiproliferative activity or for its action on the assembly of microtubules or in the cell cycle.

**Figure 5:**
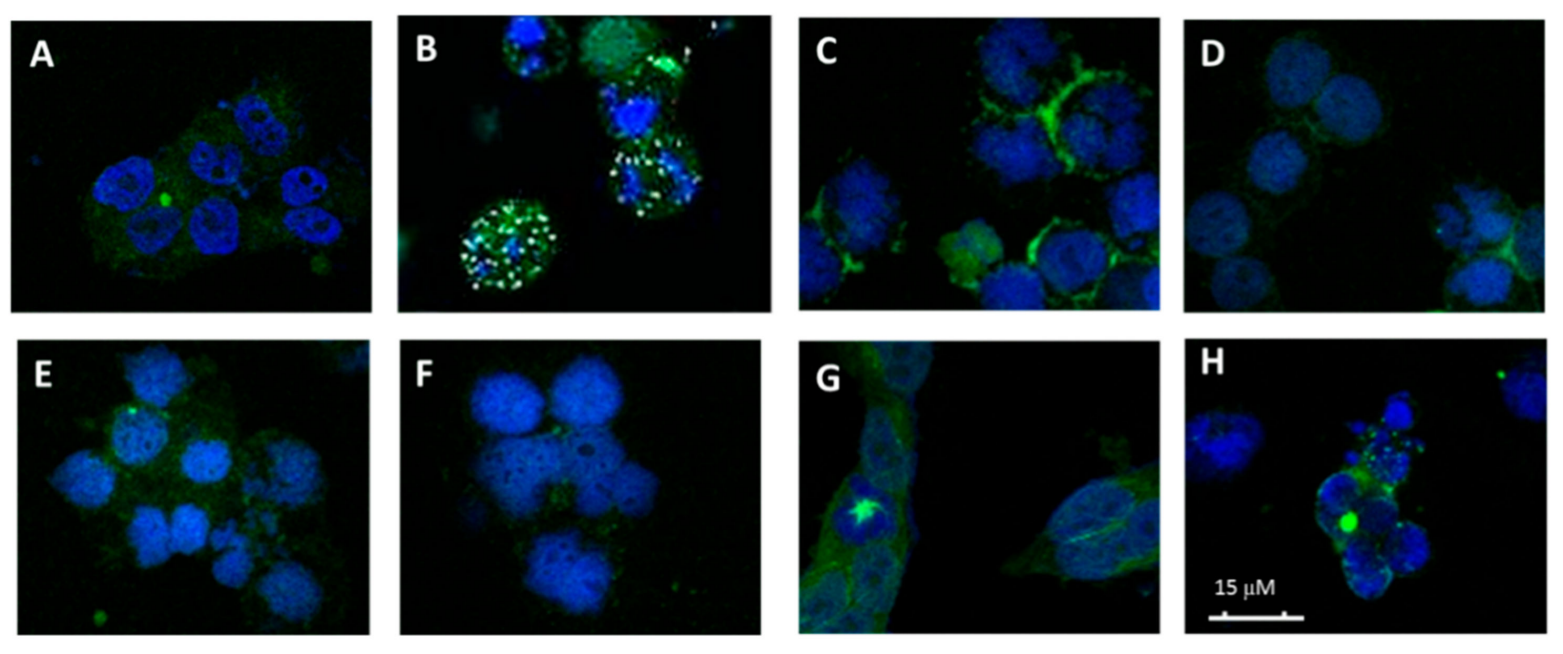
Effects on the microtubule network. (**A**) Cellular microtubules with DMSO, (**B**) 50 nM AmCA-4, (**C**) 0.5 μM **7.1** (Fmoc-Gly), (**D**) 2 µM **8.1** (Gly), (**E**) 3 µM **8.2** (l-Ala), (**F**) 0.5 µM **8.5** (l-Phe), (**G**) 40 µM de **10.1** (d-Val) and (**H**) 0.5 µM **10.2** (d-Leu).

It can be seen in Figure 5 (panel B) that AmCA-4 at a concentration of 50 nM caused a total depolymerization of microtubules. On the other hand, derivatives **7.1** (Fmoc-Gly, panel C); **8.1** (Gly, panel D); **8.2** (l-Ala, panel E) and **10.1** (d-Val, panel G) also caused the almost total depolymerization of the microtubule network, although at concentrations higher than AmCA-4. Derivatives **8.5** (L-Phe, panel F) and **10.1** (d-Leu, panel H) are much less active, although some alterations in the microtubule network can be observed.

### 2.6. Effects on VEGF, hTERT and c-Myc Genes

**Figure 6:**
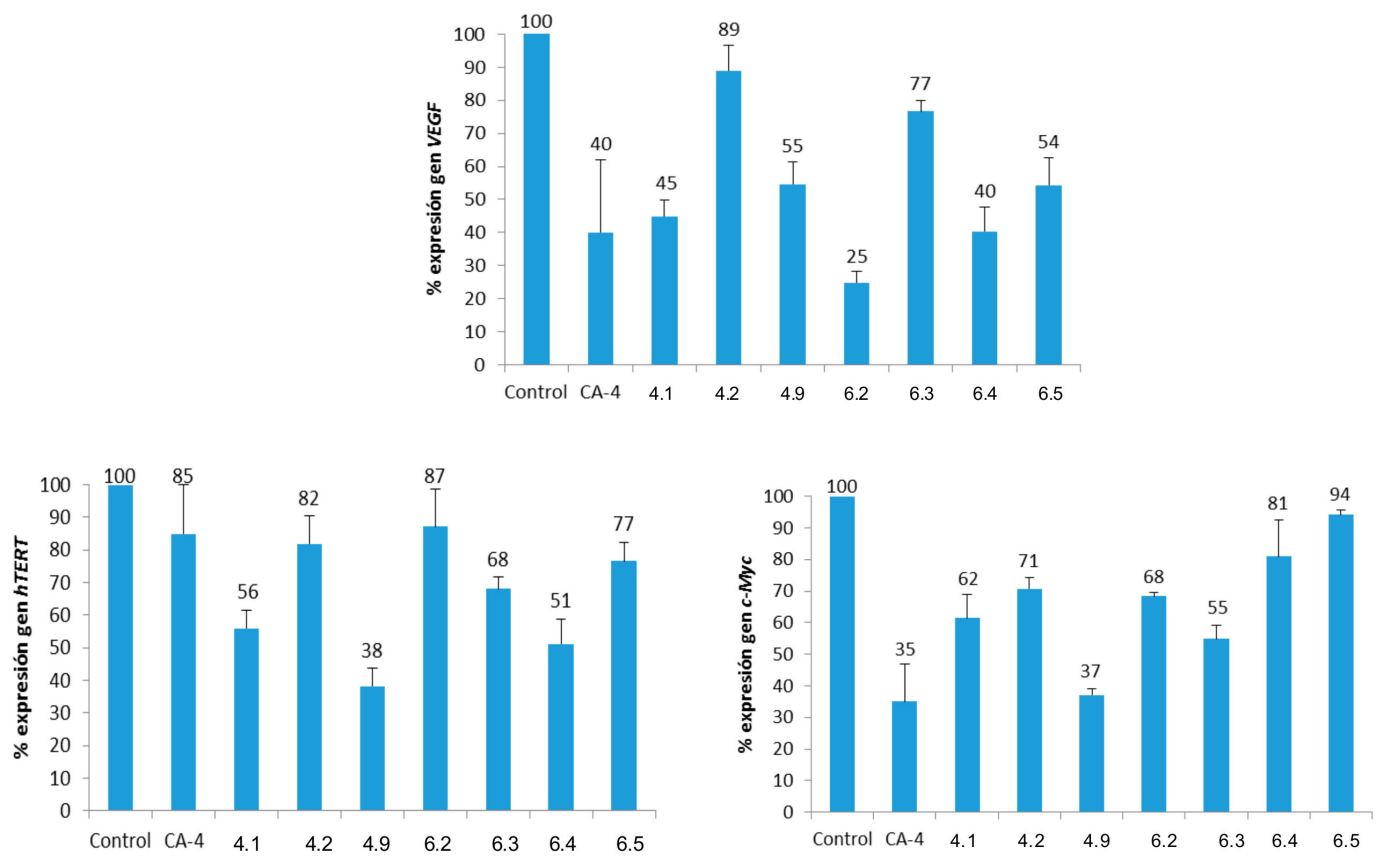
Percentages of gene expression (normalized to β-actin). Concentrations: CA-4 (15 μM); **4.1** (Gly, 5 μM); **4.5** (L-Pro, 25 μM); **4.9** (L-Met, 25 μM); **6.2** (d-Leu, 25 μM); **6.3** (d-Phe, 25 μM); **6.4** (d-Pro, 25 μM) and **6.5** (d-Ser, 10 μM).

The effect on the expression of *VEGF*, *hTERT* and *c-Myc* genes has been studied for CA-4 and its derivatives that show selectivity indexes higher than 0.9 on the HT-29 cell line. The studied derivatives, together with the concentrations to which they have been tested, are CA-4 (15 μM); **4.1** (Gly, 5 μM); **4.5** (l-Pro, 25 μM); **4.9** (l-Met, 25 μM); **6.2** (d-Leu, 25 μM); **6.3** (d-Phe, 25 μM); **6.4** (d-Pro, 25 μM) and **6.5** (d-Ser, 10 μM). The expression of the genes was determined by RT-qPCR methodology, as described in the Materials and Methods section. The results are presented in Figure 6, which shows the gene expression percentage after 48 h of incubation in the presence of DMSO (control), CA-4 and each of the selected derivatives.

Some derivatives are quite active in the inhibition of *VEGF* gene expression, such as compounds **6.2** (D-Leu), which shows 40% of gene expression and **6.4** (D-Pro), which improves (25% gene expression) the inhibition caused by CA-4 (40%). In the case of the *hTERT* gene, almost all the derivatives provoke better percentages of inhibition than CA-4, although it is worth mentioning that this compound only manages to reduce the expression of the gene up to 85%. Derivative **4.9** (l-Met) is the most active, expressing only 38% with respect to control. In the case of the c-Myc gene, none of the derivatives improves the inhibitory capacity of CA-4, which expresses only 35% of this gene. However, **4.9** (l-Met) downregulates the expression of the c-Myc gene to 37%. Thus, **4.9** (l-Met) can be considered the most active compound of these CA-4 derivatives, as it reduces the expression of the three genes studied to 37–55%.

**Figure 7:**
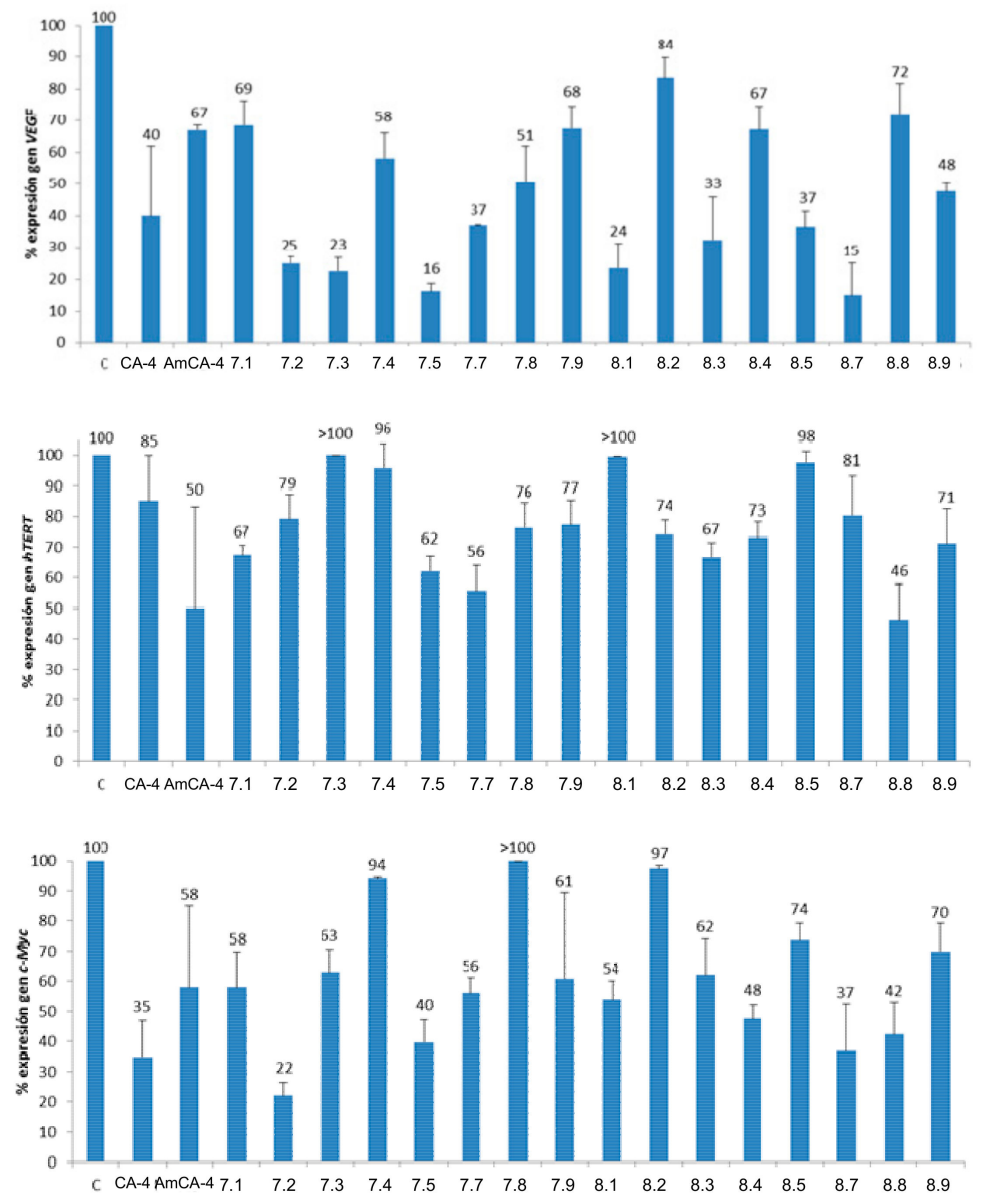
Percentages of gene expression. Concentrations: CA-4 (15 μM), AmCA-4 (15 nM), **7.1** (7 nM), **7.2** (1.5 nM), **7.3** (4 nM), **7.4** (15 nM), **7.5** (2 nM), **7.7** (7 nM), **7.8** (15 nM), **7.9** (30 nM), **8.1** (7 nM), **8.2** (3 nM), **8.3** (10 nM), **8.4** (1.5 nM), **8.5** (4 nM), **8.7** (7 nM), **8.8** (3 nM) and **8.9** (4 nM).

As regards AmCA-4 derivatives, the effect on the expression of the *VEGF*, *hTERT* and *c-Myc* genes has been studied on those that show IC_50_ in the low nanomolar range on the HT-29 cell line and/or on compounds whose selectivity indexes with respect to this cell line are higher than 1. The studied compounds, together with the concentrations to which they have been tested, are: CA-4 (15 μM), AmCA-4 (15 nM), **7.1** (7 nM), **7.2** (1.5 nM), **7.3** (4 nM), **7.4** (15 nM), **7.5** (2 nM), **7.7** (7 nM), **7.8** (15 nM), **7.9** (30 nM), **8.1** (7 nM), **8.2** (3 nM), **8.3** (10 nM), **8.4** (1.5 nM), **8.5** (4 nM), **8.7** (7 nM), **8.8** (3 nM) and **8.9** (4 nM). In all cases, the concentration chosen for a given compound is slightly lower than its IC_50_ value. The results are presented in Figure 7, which shows the gene expression percentage after 48 h of incubation in the presence of DMSO (control), CA-4, Am-CA-4 and each of the selected derivatives.

As regards the expression of the *VEGF* gene, half of the tested AmCA-4 derivatives show expression percentages lower than 40%, values lower than those of CA-4 and AmCA-4. The most outstanding percentages are those of **7.2** (Fmoc-L-Ala, 25%), **7.3** (Fmoc-L-Val, 23%), **7.5** (Fmoc-L-Phe, 16%), **8.1** (Gly, 24%) and **8.7** (L-Thr) (15%). In the expression of the *hTERT* gene, AmCA-4 derivatives are not as active as in the case of the *VEGF* gene. The most prominent compound is **8.8** (L-Tyr, 46%), which improves the percentages of both CA-4 and AmCA-4, while derivatives **7.1** (Fmoc-Gly), **7.5** (Fmoc-L-Phe), **7.7** (Fmoc-L-Thr) and **8.3** (L-Val), with expression percentages lower than 70%, are more active than CA-4 and at the same level as AmCA-4. For the c-Myc gene, most derivatives exert a prominent effect, particularly **7.2** (Fmoc-L-Ala), which reduces the expression up to 22% and is more active than the reference compounds CA-4 and AmCA-4. Compounds **7.5** (Fmoc-L-Phe, 40%), **8.4** (L-Leu, 48%), **8.7** (L-Thr, 37%) and **8.8** (L-Tyr, 42%) also deserve mention, as they are able to reduce the expression of the c-Myc gene by half. The most notable compound in the inhibition of gene expression is **7.5** (Fmoc-L-Phe), which shows very active in the inhibition of the three genes studied.

## 3. Discussion

**Figure 8:**
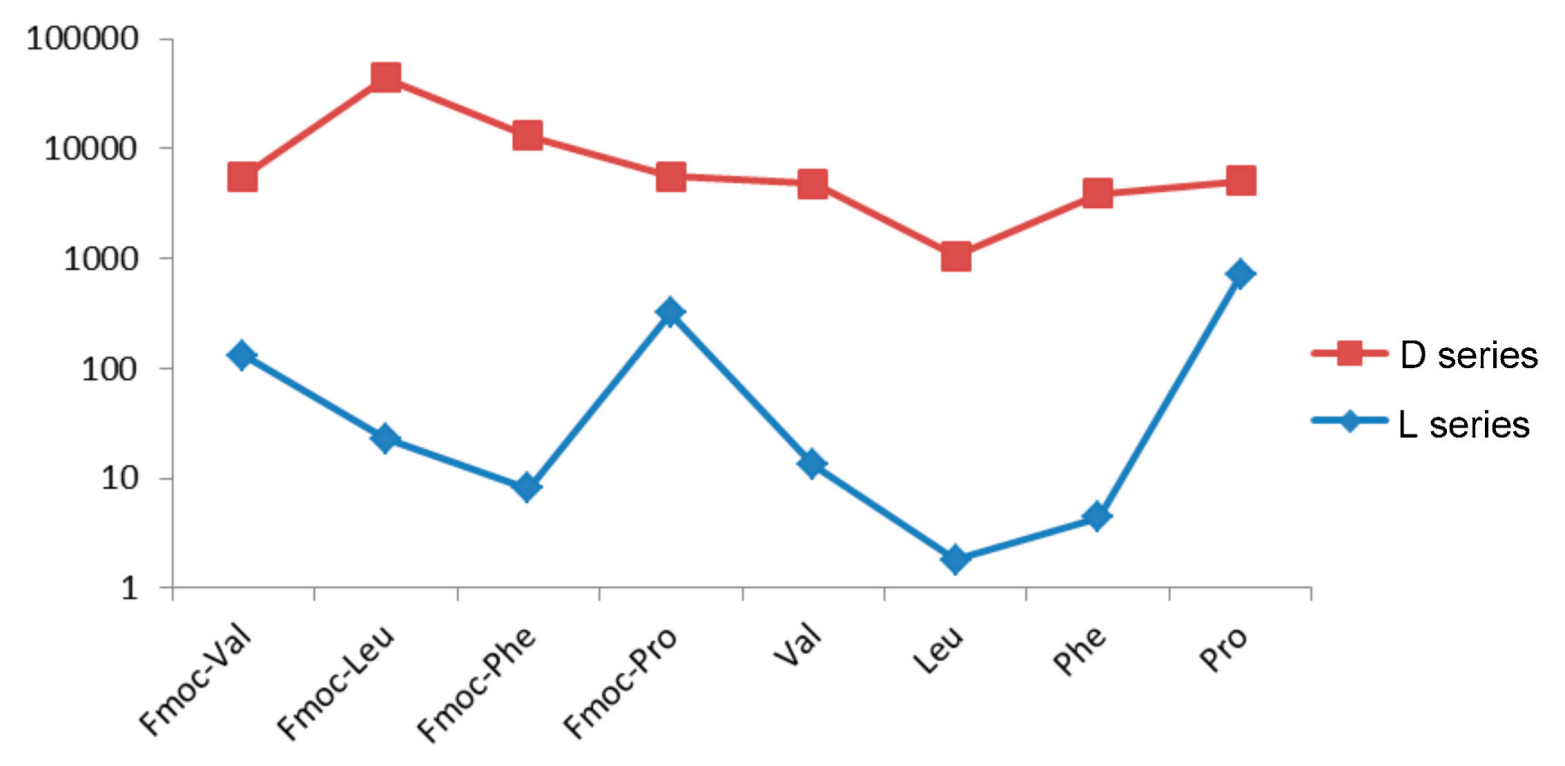
IC_50_ values (μM) in the logarithmic scale for the HT-29 cell line.

As regards antiproliferative activities, CA-4 derivatives show IC_50_ values in the low micromolar range for all the cell lines studied. Derivatives that incorporate d-amino acids, together with the derivative **4.9** (l-Met), exhibit, in general, the best therapeutic safety margins, with values higher than 1 for HT-29 and MCF-7 and close to this value on the A-549 line. AmCA-4 derivatives show antiproliferative activities ranging from low nanomolar values for l-derivatives (except for l-Pro derivatives, which show IC_50_ values in the high nanomolar range) to low micromolar values for d-derivatives. Figure 8 logarithmically compares IC_50_ values on the HT-29 line for l- and d-AmCA-4. Thus, differences of 2-4 orders of magnitude can be seen in the antiproliferative activities of l-and d-AmCA-4 derivatives, even though these differences are much more attenuated in the case of proline derivatives.

In relation to antimitotic activities, CA-4 derivatives bearing d-amino acids do not exert an appreciable effect on the cell cycle, while those with d-amino esters do exert a certain antimitotic effect, accumulating between 30–50% of cells in the G2/M phase. However, derivatives with l-amino acids exert a greater effect on the cell cycle, even improving the effect of CA-4. The most active derivatives are those bearing hydroxylated chain amino esters, such as **3.7** (l-Thr-OMe) and **3.8** (l-Tyr-OMe), as well as those bearing amino acids with short hydrophobic side chains, such as **4.2** (l-Val) and **4.3** (l-Leu). AmCA-4 derivatives tested have generally been quite active at concentrations lower than those used with CA-4 derivatives. Thus, F-moc-protected derivatives with short chains, **7.1** (Fmoc-Gly), **7.2** (Fmoc-l-Ala) and **8.2** (l-Ala), and those containing hydroxylated amino acids (**8.7** l-Thr and **8.8** l-Tyr) or sulfur-containing amino acids (**8.9** l-Met) accumulate more than 50% of cells in the G2/M phase. In addition, compounds **8.2** (l-Ala) and **8.7** (l-Thr) are partial inhibitors of the in vitro polymerization of tubulin.

In gene inhibition expression, CA-4 derivatives are generally more active against the *VEGF* gene, with **6.2** (d-Leu) being particularly active. The most active compound of that group, however, is **4.9** (l-Met), which downregulates the expression of the three genes studied more than 55%. The most active AmCA-4 derivatives on *VEGF* and *c-Myc* genes are **7.2** (Fmoc-l-Ala), **7.5** (Fmoc-l-Phe) and **8.7** (l-Thr), since they downregulate the expression of these two genes more than 60%. For the set of the three genes, the most notable derivative is **7.5** (Fmoc-l-Pro), since it is able to reduce *VEGF* expression to 16%, and *c-Myc* and *hTERT* expression to 56%.

## 4. Materials and Methods

### 4.1. Chemistry

All chemicals were supplied by Sigma-Aldrich Inc. (Darmstadt, Germany). NMR spectra were measured at 25 °C in an Avance III HD NMR spectrometer (Bruker Biospin AG, Bruker Española, Madrid, Spain). The signals of the deuterated solvent (CDCl_3_) were taken as the reference. Multiplicity assignments of ^13^C signals were made by means of the DEPT pulse sequence. Complete signal assignments in ^1^H and ^13^C NMR spectra were made with the aid of 2D homo- and heteronuclear pulse sequences (COSY, HSQC and HMBC). High-resolution mass spectra were run by the electrospray mode (ESMS). IR data were measured with oily films on NaCl plates (oils) and given only for relevant functional groups (C=O and NH). Experiments which required an inert atmosphere were carried out under dry N_2_ in flame-dried glassware. Commercially available reagents were used as received.

#### 4.1.1. Synthesis of Ethyl (*Z*)-2-(2-methoxy-5-(3,4,5-trimethoxystyryl)phenoxy)acetate **1**

K_2_CO_3_ (2 mmol) was added to a solution of combretastatin A-4 (316 mg, 1 mmol) in anhydrous DMF (20 mL), and the reaction mixture was stirred, under N_2_ atmosphere, for one hour at room temperature. Then, ethyl bromoacetate (1.5 mmol) was added, and the resulting mixture was stirred at room temperature for 24 h. The mixture was then poured into a saturated aqueous NH_4_Cl solution and extracted with Et_2_O (3 × 30 mL), and the combined organic extracts were dried over anhydrous MgSO_4_. After filtration and evaporation of the solvent under reduced pressure, the resulting residue was chromatographed on silica gel with Hexanes-AcOEt (7:3) to afford 378 mg (94%) of compound **1**.

#### 4.1.2. Synthesis of (*Z*)-2-(2-Methoxy-5-(3,4,5-trimethoxystyryl)phenoxy)acetic acid **2**

NaOH (10 mmol) in H_2_O (2.6 mL) was added to a solution of ester 1 (201 mg, 0.5 mmol) in EtOH (25 mL). The mixture was stirred for one hour at room temperature, and then EtOH was removed under reduced pressure. The residue was acidified with 2 M HCl and extracted with CH_2_Cl_2_ (3 × 30 mL), and the combined organic extracts were dried over anhydrous MgSO_4_. After filtration and evaporation of the solvent under reduced pressure, the resulting residue was recrystallized from the Hexanes-AcOEt mixture (8:2) to afford 170 mg (91%) of compound **2**.

#### 4.1.3. General Procedure for the Synthesis of *N*-acyl-amino Ester Derivatives **3** and **5**

DMAP (1.14 mmol, 2.28 eq.) and the corresponding amino ester hydrochloride (0.57 mmol, 1.14 eq.) were added to a solution of acid **2** (0.5 mmol, 1 eq) in anhydrous DMF (10 mL). The reaction mixture was cooled at 0 °C, and then EDCI·HCl (0.55 mmol, 1.1 eq.) was added. The resulting mixture was stirred for 2 h at 0 °C and then brought to room temperature and left to react for 2 h. The mixture was then poured into a saturated aqueous NH_4_Cl solution and extracted with AcOEt (3 × 20 mL). The combined organic extracts were washed with H_2_O and brine and dried over anhydrous MgSO4. After filtration and evaporation of the solvent under reduced pressure, the resulting residue was chromatographed on silica gel with Hexanes-AcOEt (6:4), affording the corresponding *N-*acyl-aminoester derivatives **3** and **5**.

#### 4.1.4. General Procedure for the Synthesis of N-acyl-amino Acid Derivatives **4** and **6**

LiOH·H_2_O (0.45 mmol, 3 eq.) in H_2_O (2.2 mL) was added to a solution of amino ester **3** or **5** (0.15 mmol, 1 eq.) in MeOH (2.2 mL). The mixture was stirred for one hour at room temperature and then poured into H_2_O and washed with Et_2_O. The resulting aqueous phase was acidified with 2M HCl and extracted with AcOEt (3 × 30 mL). The combined organic extracts were dried over anhydrous MgSO_4_. Filtration and evaporation of the solvent under reduced pressure afforded the corresponding amino acid derivatives **4** and **6**.

#### 4.1.5. General Procedure for the Synthesis of *N*-Fmoc-Protected Derivatives **7** and **9**

DCC (0.47 mmol, 1.2 eq.); the *N*-Fmoc-protected amino acid (0.472 mmol, 1.2 eq) and HOBt·H_2_O (0.47 mmol, 1.2 eq) were added to a solution of the 3′-aminocombretastatin (0.39 mmol, 1 eq) in anhydrous DMF (1.3 mL). The reaction mixture was stirred under N_2_ atmosphere for 16 h. Then, it was filtered, and the filtrate was poured into a saturated aqueous NH_4_Cl solution and then extracted with AcOEt (3 × 15 mL). The combined organic extracts were washed 2 times with water and brine and dried over anhydrous MgSO_4_. After filtration and evaporation of the solvent under reduced pressure, the resulting residue was chromatographed on silica gel with Hexanes-AcOEt (6:4) to afford the corresponding *N*-Fmoc-protected derivatives **7** and **9**.

#### 4.1.6. General Procedure for the Synthesis of **8** and **10** Derivatives by *N*-Fmoc Deprotection

Anhydrous KF (1.5 mmol, 10 eq) and 18-crown-6 (0.015 mmol, 0.1 eq.) were added to a solution of the *N*-Fmoc-protected derivative (0.15 mmol, 1 eq.) in anhydrous DMF (5.5 mL). The resulting mixture was stirred at room temperature under N_2_ atmosphere for 4 h. Then, the reaction mixture was poured into a saturated aqueous NH_4_Cl solution and extracted with AcOEt (3 × 25 mL). The combined organic extracts were washed sequentially with water (×2) and with brine and dried over anhydrous MgSO_4_. After filtration and evaporation of the solvent under reduced pressure, the resulting residue was chromatographed on silica gel with AcOEt to afford derivatives **8** and **10**.

### 4.2. Biological Assays

#### 4.2.1. Cell Culture

Cell culture media were purchased from Gibco (Grand Island, NY, USA). Fetal bovine serum (FBS) was a product of Harlan-Seralab (Belton, U.K.). Supplements and other chemicals not listed in this section were obtained from Sigma Chemical Co. (St. Louis, MO, USA). Plastics for cell culture were supplied by Thermo Fischer Scientific Inc., Madrid, Spain. All tested compounds were dissolved in DMSO at a concentration of 10 μg/mL and stored at −20 °C until use. HT-29, MCF-7, A-549 and HEK-293 cell lines were maintained in a Dulbecco’s modified Eagle’s medium (DMEM) containing glucose (1 g/L), glutamine (2 mM), penicillin (50 IU/mL), streptomycin (50 μg/mL) and amphotericin B (1.25 μg/mL), supplemented with 10% FBS. Cells from human non-small lung carcinoma (clone A549; ATCC n°: CCL2, MD, USA) were routinely grown at 37 °C in a humidified atmosphere of 5% CO_2_. Cells were maintained in a standard medium composed of RPMI 1640 (Lonza, Group Ltd., Basel, Switzerland), supplemented with 10% fetal bovine serum (FBS), 2 mM l-glutamine and 1% penicillin and streptomycin (Invitrogen). Cells were free from mycoplasma as determined by MycoAlert tests (Lonza, Group Ltd., Basel, Switzerland).

#### 4.2.2. Cell Proliferation

For cell viability assay on the cell line A549, cells harvested from subconfluent monolayers were seeded at 25,000/mL in a 96-well microtiter plate (Cambridge Technology, France) and cultured 24 h under standard conditions. Standard medium was then replaced by fresh medium containing no drugs (control or “0”) or compounds at different concentrations. The surviving cells were quantified after 72 h by the MTT assay, according to the manufacturer’s instructions. Briefly, 20 µL of 3-(4,5-dimethylthiazol-2-yl)-2,5-diphenyltetrazolium bromide (MTT) solution at 5 mg·mL^−1^ were added to cells for 2 h at 37 °C. The supernatant was discarded and replaced by 200 µL of DMSO to dissolve formazan crystals. The absorbance was then read at 540 nm by spectrophotometry. For all concentrations of the compound, cell viability was expressed as the percentage of the ratio between the mean absorbance of treated cells and the mean absorbance of untreated cells. Three independent experiments were performed, and the IC_50_ values (i.e., concentration half inhibiting cell proliferation) were graphically determined.

A total of 5 × 10^3^ HT-29, MCF-7 or HEK-293 cells in a total volume of 100 μL of their respective growth media were incubated with serial dilutions of the tested compounds. The 3-(4,5-dimethylthiazol-2-yl)-2,5-diphenyltetrazolium bromide (MTT; Sigma Chemical Co.) dye reduction assay in a 96-well microplate was used, as previously described [33]. After 3 days of incubation (37 °C, 5% CO_2_ in a humid atmosphere), 10 μL of MTT (5 mg/mL in phosphate-buffered saline; PBS) was added to each well, and the plate was incubated for a further 4 h (37 °C). The resulting formazan was dissolved in 150 μL of 0.04 N HCl/2-propanol and read at 550 nm. All determinations were carried out in triplicate.

#### 4.2.3. Fluorescence Microscopy

For immunofluorescence microscopy of the microtubule network, 10^6^ cells were plated on cover glass and incubated with the different drugs. Cells were then fixed in 3.7% formaldehyde (in PBS pH 7.4) for 20 min at room temperature and permeabilized with PBS-Triton X-100 0.5% for 10 min at room temperature. Direct immunostaining was carried out for 2 h at room temperature with a primary FITC-conjugated anti-α-tubulin antibody (dilution 1:400 in PBS-BSA 1% from a 1 mg/mL solution; monoclonal antibody, clone DM1A; Sigma-Aldrich, Darmstadt, Germany.). Next, cells were incubated for 45 min at room temperature in darkness with secondary Alexa Fluor 488-conjugated antibodies (dilution 1:200 each from 1.5 mg/mL; Molecular Probe, France). Then, cells were washed in PBS and cover glasses were mounted with a drop of ProLong^®^ antifade solution (Invitrogen). The cytoskeleton was imaged by a confocal laser scanning microscope (CLSM) Leica SP5 with a Leica inverted microscope, equipped with a Plan-Apochromat 63× oil immersion objective (NA = 1.4). Each image was recorded with the CLSM’s spectral mode selecting specific domains of the emission spectrum. The FITC fluorophore was excited at 488 nm with an argon laser, and its fluorescence emission was collected between 496 nm and 535 nm.

#### 4.2.4. Cell Cycle

For flow cytometric analysis of DNA content, 4 × 10^5^ cells in exponential growth were treated with the IC_50_ value and ten times this value of each compound; thus, 14 nM and 140 nM for CLC, 18 nM and 180 nM compound **2**, 133 nM and 1.33 µM compound **3**, 25 nM and 250 nM compound **4**, 62 nM and 620 nM compound **5** and 72 nM and 720 nM compound **6** for 24 h. The supernatant and trypsinated cells were harvested and then centrifuged (1200 rpm, 5 min, 4 °C). The cell pellet was resuspended in 1 mL of cold 70% ethanol for 30 min at −20 °C. Cells were centrifuged (2000 rpm, 5 min, 4 °C) to remove ethanol, and then the cell pellet was resuspended in the staining mix containing 50 µg/mL propidium iodide (Molecular Probes, France) and 100 µg/mL RNAse A (Sigma, France) in PBS for 20 min at room temperature in darkness. Samples were analyzed on a Becton Dickinson FACScan flow cytometer using the CellQuest software, which was also used to determine the percentage of cells in the G2/M phases of the cell cycle. PI was excited at 488 nm, and fluorescence analyzed at 620 nm on channel Fl-2.

#### 4.2.5. Gene Expression Evaluation by RT-qPCR-assay

HT-29 cells at 70–80% confluence were collected, and 1.5 × 105 cells were placed in a six-well plate in 1.5 mL of the medium. After 24 h, cells were incubated with the corresponding compounds for 48 h. Cells were collected, and the total cellular RNA from the HT-29 cells was isolated using an Ambion RNA Extraction Kit, according to the manufacturer’s instructions. The cDNA was synthesized by MMLV-RT with 1–21 μg of extracted RNA and oligo(dT)15, according to the manufacturer’s instructions. Genes were amplified by a thermal cycler and StepOnePlus™ TaqMan^®^ probes. TaqMan^®^ Gene Expression Master Mix Fast, containing the appropriate buffer for the amplification conditions, dNTPs, thermostable DNA polymerase enzyme and a passive reference probe, was used. To amplify each of the genes, the predesigned primers sold by Life Technologies TaqMan^®^ Gene Expression Assays Hs99999903-m1 (β-actin), Hs00900055-m1 (VEGF), Hs00972646-m1 (*hTERT*) and Hs00153408-m1 (*c-Myc*) were used.

## Figures and Tables

**Table 1:** IC_50_ values (μM) and SI indexes for CA-4 derivatives ^a^.

Comp.	HT-29	MCF-7	A-549	HEK-293	SI_A_^b^	SI_B_^c^	SI_c_^d^
**CA-4**	4.2 ± 0.5	1.0 ± 0.2	0.428 ± 0.004	25 ± 3	5.9	25	58
**1**	7 ± 4	8 ± 4	9 ± 1	3.6 ± 0.5	0.5	0.4	0.4
**2**	33 ± 6	11 ± 5	38 ± 5	12 ± 5	0.4	1.1	0.3
**3.1**	12 ± 2	8 ± 2	26 ± 2	9 ± 1	0.7	1.1	0.3
**3.2**	24 ± 4	12 ± 6	24 ± 1	5 ± 2	0.2	0.4	0.2
**3.3**	29 ± 6	19 ± 6	8 ± 1	9 ± 2	0.3	0.5	1.1
**3.4**	28 ± 7	8 ± 2	9 ± 2	4 ± 1	0.14	0.5	0.4
**3.5**	48 ± 5	24 ± 2	50 ± 10	20 ± 1	0.4	0.8	0.4
**3.6**	9 ± 3	7 ± 3	18 ± 4	7 ± 2	0.8	1.0	0.4
**3.7**	18 ± 7	12 ± 4	19 ± 1	14 ± 2	0.8	1.2	0.7
**3.8**	16 ± 5	12 ± 4	18 ± 1	10 ± 2	0.6	0.8	0.6
**3.9**	16 ± 7	10 ± 3	10 ± 2	8 ± 1	0.5	0.8	0.9
**4.1**	5 ± 1	6 ± 1	24 ± 4	5 ± 1	0.9	0.8	0.2
**4.2**	12 ± 5	7 ± 1	17 ± 2	4 ± 1	0.4	0.6	0.3
**4.3**	15 ± 5	16 ± 6	14 ± 2	9 ± 2	0.6	0.6	0.6
**4.4**	54 ± 9	45 ± 4	100 ± 20	47 ± 3	0.9	1.0	0.5
**4.5**	26 ± 13	83 ± 16	110 ± 10	45 ± 16	1.7	0.5	0.4
**4.6**	39 ± 16	27 ± 9	36 ± 4	17 ± 1	0.4	0.6	0.5
**4.7**	34 ± 11	40 ± 9	41 ± 3	23 ± 9	0.7	0.6	0.6
**4.8**	55 ± 5	43 ± 7	30 ± 7	33 ± 1	0.6	0.8	1.1
**4.9**	32 ± 6	13 ± 2	>300	40 ± 4	1.3	3.1	<0.1
**5.1**	27 ± 7	9 ± 3	5 ± 1	3.9 ± 0.6	0.1	0.4	0.8
**5.2**	26 ± 1	16 ± 3	22 ± 3	13 ± 3	0.5	0.8	0.6
**5.3**	12 ± 4	6 ± 1	10 ± 3	3.7 ± 1.6	0.3	0.7	0.4
**5.4**	50	10 ± 4	15 ± 1	5.7 ± 0.8	0.1	0.6	0.4
**5.5**	11 ± 2	7 ± 2	28 ± 1	9 ± 3	0.8	1.3	0.3
**6.1**	45 ± 5	46 ± 3	54 ± 7	39 ± 6	0.9	0.8	0.7
**6.2**	47 ± 9	25 ± 2	68 ± 1	49 ± 12	1.0	2.0	0.7
**6.3**	27 ± 5	43 ± 3	60 ± 4	46 ± 6	1.7	1.1	0.8
**6.4**	50 ± 11	55 ± 13	95 ± 14	53 ± 9	1.1	1.0	0.6
**6.5**	13 ± 1	12 ± 1	>150	14 ± 4	1.1	1.2	<0.09

^a^ IC_50_ values are expressed as the compound concentration (μM) that inhibits the cell growth by 50% after 48 h of treatment. Data are the average (±SD) of three experiments: ^b^ SI_A_ = IC_50_ (HEK-293)/IC_50_ (HT-29). ^c^ SI_B_ = IC_50_ (HEK-293)/IC_50_ (MCF-7). ^d^ SI_B_ = IC_50_ (HEK-293)/IC_50_ (A-549).

**Table 2:** IC_50_ values (nM) and SI indexes for AmCA-4 derivatives ^a^.

Comp.	HT-29	MCF-7	A-549	HEK-293	SI_A_^b^	SI_B_^c^	SI_c_^d^
**CA-4**	4200 ± 500	1000 ± 200	428 ± 4	25000 ± 3000	5.9	25	60
**AmCA-4**	22 ± 1	8 ± 1	117 ± 7	7 ± 1	0.3	0.9	0.06
**7.1**	7 ± 4	4.8 ± 0.5	186 ± 11	3.5 ± 1.9	0.5	0.7	0.02
**7.2**	1.1 ± 0.4	1.9 ± 0.8	242 ± 13	1.5 ± 0.6	1.4	0.8	0.01
**7.3**	130 ± 40	100 ± 50	870 ± 60	60 ± 20	0.5	0.6	0.07
**7.4**	23 ± 10	9 ± 2	490 ± 13	14 ± 3	0.6	1.6	0.03
**7.5**	8 ± 3	14 ± 4	270 ± 50	2.3 ± 2	0.3	0.2	0.01
**7.6**	320 ± 140	58 ± 12	1330 ± 90	130 ± 30	0.4	2.2	0.1
**7.7**	9 ± 3	25 ± 12	422 ± 8	13 ± 2	1.4	0.5	0.03
**7.8**	18 ± 5	12 ± 2	8900 ± 1200	16 ± 2	0.9	1.3	0.01
**7.9**	33 ± 20	5.1 ± 0.3	407 ± 23	8 ± 4	0.2	1.6	0.02
**8.1**	10 ± 2	9 ± 4	400 ± 70	10 ± 2	1.0	1.1	0.02
**8.2**	3.0 ± 0.3	4.8 ± 0.5	611 ± 16	4.0 ± 0.1	1.3	0.8	0.01
**8.3**	13.3 ± 0.5	13 ± 2	490 ± 100	7.6 ± 0.3	0.6	0.6	0.02
**8.4**	1.8 ± 0.4	1.8 ± 1.2	167 ± 23	1.7 ± 0.8	0.9	0.9	0.01
**8.5**	4.4 ± 2.5	2.3 ± 1.5	141 ± 3	3.5 ± 1.9	0.8	1.5	0.02
**8.6**	700 ± 200	3100 ± 600	1200 ± 300	2400 ± 1400	3.4	0.8	2.0
**8.7**	8 ± 2	12 ± 1	410 ± 40	14 ± 3	1.8	1.1	0.03
**8.8**	2.2 ± 1.2	4.9 ± 0.2	206 ± 20	2.7 ± 1.4	1.2	0.5	0.01
**8.9**	4.9 ± 0.1	3.4 ± 1.2	90 ± 30	3.7 ± 0.3	0.7	1.1	0.04
**9.1**	5500 ± 700	4800 ± 500	7340 ± 230	5100 ± 700	0.9	1.1	0.7
**9.2**	44000 ± 6000	3100 ± 500	48000 ± 3000	2400 ± 400	0.05	0.8	0.05
**9.3**	13000 ± 5000	4500 ± 1700	18500 ± 1700	11200 ± 1500	0.9	2.5	0.6
**9.4**	5300 ± 300	5300 ± 1100	12800 ± 800	3100 ± 1100	0.6	0.6	0.2
**10.1**	4800 ± 600	4700 ± 700	9900 ± 1300	2400 ± 300	0.5	0.5	0.2
**10.2**	1050 ± 140	2000 ± 900	157 ± 7	430 ± 40	0.4	0.2	2.7
**10.3**	3900 ± 800	2000 ± 800	2900 ± 500	3500 ± 900	0.9	1.8	1.2
**10.4**	4400 ± 1900	600 ± 140	7030 ± 220	3000 ± 600	0.7	5.0	0.4

^a^ IC_50_ values are expressed as the compound concentration (nM) that inhibits the cell growth by 50% after 48 h of treatment. Data are the average (±SD) of three experiments: ^b^ SI_A_ = IC_50_ (HEK-293)/IC_50_ (HT-29). ^c^ SI_B_ = IC_50_ (HEK-293)/IC_50_ (MCF-7). ^d^ SI_B_ = IC_50_ (HEK-293)/IC_50_ (A-549).

**Table 3:** Critical concentration (CrC) values for the assembly of purified tubulin in GAB in the presence of CA-4 derivatives.

Comp.	CrC (µM)	Comp.	CrC (µM)	Comp.	CrC (µM)
**Control**	7.9 ± 1.2	**3.8**	13.26 ± 0.19	**5.1**	13.31 ± 0.01
**CA-4**	21.5 ± 0.8	**3.9**	8.06 ± 0.04	**5.2**	13.2 ± 0.5
**1**	13.6 ± 0.3	**4.1**	9.3 ± 0.4	**5.3**	13.1 ± 0.8
**2**	12.0 ± 0.7	**4.2**	9.3 ± 0.3	**5.4**	9 ± 3
**3.1**	9.52 ± 0.05	**4.3**	8 ± 3	**5.5**	9.2 ± 0.9
**3.2**	11.7 ± 2.3	**4.4**	11.4 ± 0.7	**6.1**	9.4 ± 1.3
**3.3**	9.4 ± 1.4	**4.5**	9.2 ± 0.5	**6.2**	11 ± 3
**3.4**	13.20 ± 0.06	**4.6**	7.9 ± 1.6	**6.3**	9.2 ± 0.4
**3.5**	10.4 ± 2.0	**4.7**	10.1 ± 1.8	**6.4**	10 ± 3
**3.6**	13.00 ± 0.10	**4.8**	8.5 ± 2.0	**6.5**	9.25 ± 0.16
**3.7**	10 ± 3	**4.9**	8.6 ± 0.4		

**Table 4:** Critical Concentration (CrC) values for the assembly of purified tubulin in GAB in the presence of AmCA-4 derivatives.

Comp.	CrC (µM)	Comp.	CrC (µM)	Comp.	CrC (µM)
**Control**	7.9 ± 1.2	**7.8**	10.1 ± 1.1	**8.9**	8.5 ± 1.2
**CA-4**	21.5 ± 0.8	**7.9**	7.1 ± 0.4	**9.1**	8.9 ± 1.5
**AmCA-4**	23 ± 3	**8.1**	10.2 ± 1.8	**9.2**	6.5 ± 0.7
**7.1**	11 ± 4	**8.2**	13.9 ± 1.5	**9.3**	6.6 ± 0.2
**7.2**	9.4 ± 0.3	**8.3**	9.3 ± 0.5	**9.4**	11.4 ± 0.2
**7.3**	6.9 ± 0.3	**8.4**	8.3 ± 0.3	**10.1**	12.8 ± 0.3
**7.4**	9.9 ± 0.8	**8.5**	9.1 ± 0.9	**10.2**	7.0 ± 0.5
**7.5**	8.1 ± 1.6	**8.6**	8.4 ± 0.1	**10.3**	7.7 ± 2.1
**7.6**	6.0 ± 0.5	**8.7**	12.3 ± 0.6	**10.4**	8.7 ± 1.2
**7.7**	12.0 ± 0.3	**8.8**	7.7 ± 0.8		

**Table 5:** It_50_ values.

Compound	It_50_ (min)
**Control**	16.6 ± 1.0
**Paclitaxel**	3.2 ± 0.4
**CA-4**	21.5 ± 0.8
**AmCA-4**	23 ± 3
**7.1**	16 ± 3
**7.7**	19.2 ± 0.3
**8.1**	18.5 ± 2.1
**8.2**	48 ± 5
**8.7**	65 ± 7
**9.4**	16.5 ± 0.3
**10.1**	17.1 ± 1.3

**Table 6:** Cell cycle distribution for CA-4 and selected compounds after 24 h of treatment.

Comp.	Conc. (μM)	G0/G1	S	G2/M
**Control**	-	75 ± 4	11 ± 2	14 ± 2
**CA-4**	0.05	25 ± 1	15 ± 1	60 ± 2
**1**	50	23 ± 5	22 ± 4	55 ± 8
**3.2**	75	42 ± 3	14 ± 4	44 ± 1
**3.3**	75	45 ± 6	19 ± 2	36 ± 4
**3.4**	50	33 ± 4	16 ± 5	51 ± 2
**3.5**	100	77 ± 2	16 ± 2	7 ± 1
**3.6**	75	30 ± 9	17 ± 6	53 ± 3
**3.7**	75	25 ± 5	18 ± 1	57 ± 6
**3.8**	75	17 ± 2	18 ± 1	65 ± 1
**3.9**	75	47 ± 4	17 ± 3	36 ± 1
**4.1**	75	68	12	20
**4.2**	50	17 ± 2	16 ± 3	67 ± 4
**4.3**	75	22 ± 6	15 ± 2	63 ± 5
**4.5**	75	72	12	16
**4.9**	75	76	11	13
**5.1**	25	53 ± 8	16 ± 2	31 ± 7
**5.2**	100	43 ± 4	23 ± 4	34 ± 1
**5.3**	50	38 ± 2	19 ± 2	43 ± 1
**5.4**	75	36 ± 4	14 ± 3	50 ± 1
**6.1**	100	67 ± 2	20 ± 2	13 ± 1
**6.2**	75	85 ± 1	3 ± 2	12 ± 1
**6.3**	75	74 ± 3	11 ± 6	14 ± 2
**6.4**	75	71	14	15
**6.5**	75	64	18	18

**Table 7:** Cell cycle distribution for CA-4, AmCA-4 and selected compounds after 24 h of treatment.

Compound	Conc. (µM)	G0/G1	S	G2/M
**Control**	-	75 ± 4	11 ± 2	14 ± 2
**CA-4**	0.05	25 ± 1	15 ± 1	60 ± 2
**AmCA-4**	0.125	26 ± 1	15 ± 2	59 ± 2
**7.1**	0.5	28 ± 1	18 ± 2	54 ± 1
**7.2**	0.5	28 ± 1	19 ± 2	53 ± 2
**7.5**	2	34 ± 2	20 ± 1	46 ± 3
**7.9**	2	72 ± 2	18 ± 1	10 ± 3
**8.1**	2	32 ± 5	22 ± 2	46 ± 4
**8.2**	3	28 ± 1	14 ± 4	58 ± 5
**8.3**	2	65 ± 2	20 ± 1	15 ± 2
**8.4**	0.5	35 ± 1	17 ± 2	48 ± 1
**8.5**	0.5	46 ± 2	28 ± 11	26 ± 10
**8.7**	2	31 ± 1	18 ± 1	51 ± 2
**8.8**	0.5	30 ± 2	18 ± 1	52 ± 3
**8.9**	0.2	30 ± 1	19 ± 1	51 ± 2
**10.1**	40	26 ± 2	14 ± 1	60 ± 1
**10.2**	0.5	63 ± 4	16 ± 3	22 ± 2

## Supplementary Materials

The following are available online at https://www.mdpi.com/1420-3049/25/3/660/s1: cell cycle histograms; spectroscopic data and ^1^H-NMR, ^13^C-NMR and HR-MS spectra of compounds **1**, **2**, **3.1**–**3.9**, **4.1**–**4.9**, **5.1**–**5.5**, **6.1**–**6.5**, **7.1**–**7.9**, **8.1**–**8.9**, **9.1**–**9.4** and **10.1**–**10.4**.
