# Supplementary material for: Synthesis of Combretastatin A-4 and 3′-Aminocombretastatin A-4 derivatives with Aminoacid Containing Pendants and Study of their Interaction with Tubulin and as Downregulators of the VEGF, hTERT and c-Myc Gene Expression
Source: Molecules. 2020 Feb 4;25(3):660. doi: 10.3390/molecules25030660 (PMC7037732; doi:10.3390/molecules25030660)
Supplement: Supplementary file 1 [file molecules-25-00660-s001.pdf]

Synthesis of combretastatin A-4 and 3´-aminocombretastatin A-4 derivatives with aminoacid containing pendants and study of their interaction with tubulin and as downregulators of the *VEGF, hTERT* and *c-Myc* gene expressions

Raül Agut [1], Eva Falomir [1]*, Juan Murga[1], Celia Martín-Beltrán[1], Raquel Gil-Edo[1], Alberto Pla[1], Miguel Carda[1]*, and J. Alberto Marco[2]

[1]Departamento de Química Inorgánica y Orgánica, Universidad Jaume I, E-12071 Castellón, Spain.

[2]Departamento de Química Orgánica, Universidad de Valencia, E-46100 Burjassot, Valencia, Spain.

Supporting Information

Contents:

S-2: ***Cell cycle histograms***

S-3: ***Analytical data***

S-18: ***Graphical NMR spectra***

Cell cycle histograms

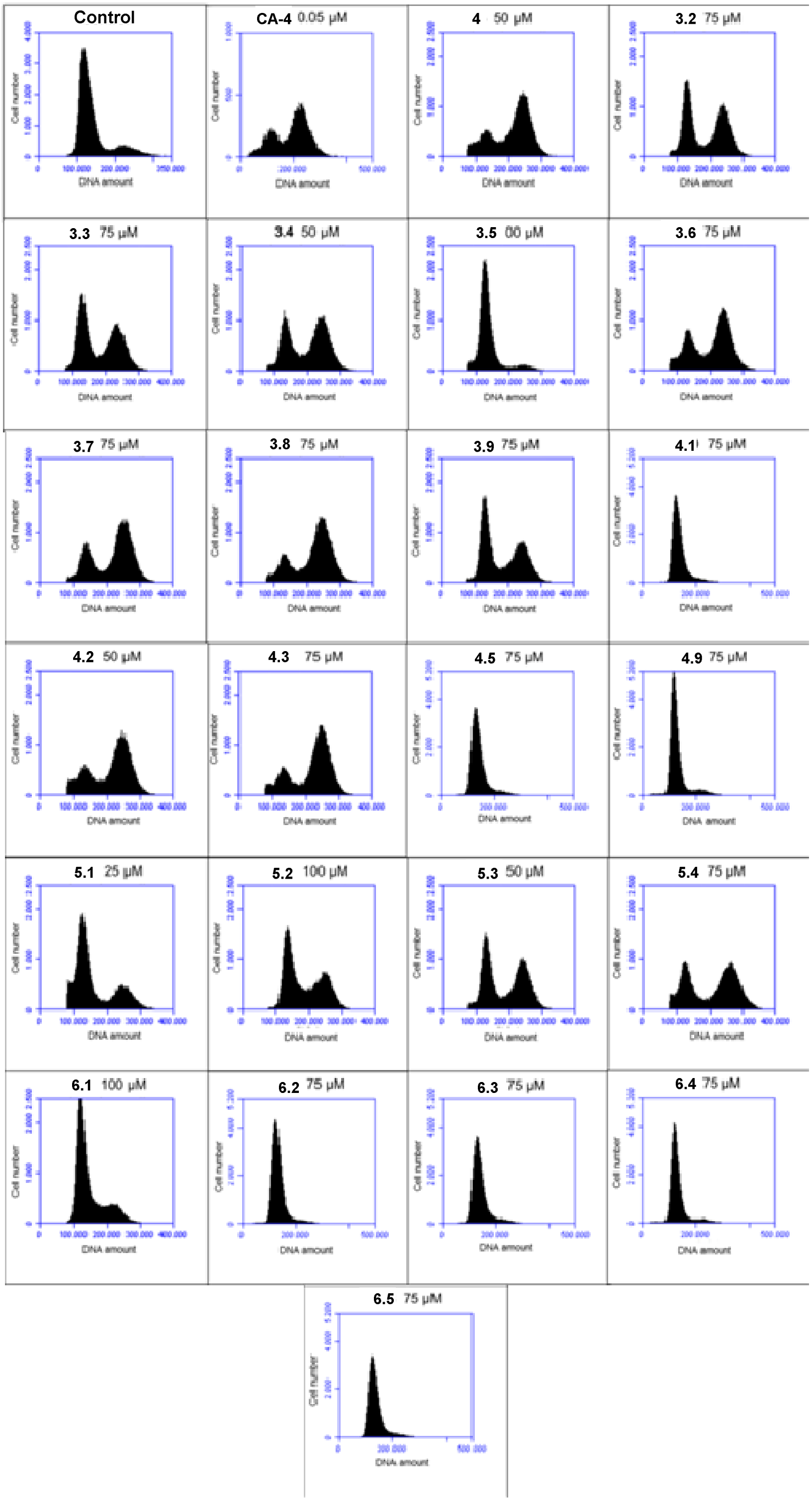

Figure 11. Cell cycle histograms for CA-4 and selected derivatives.

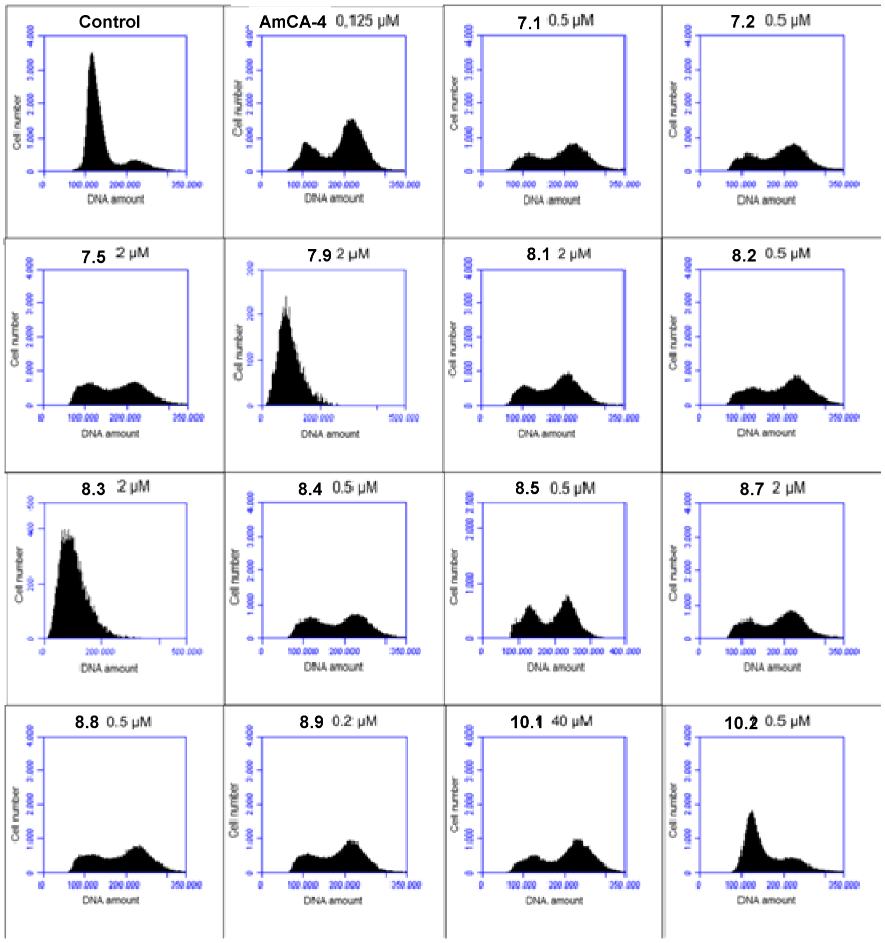

Figure 12. Cell cycle histograms for AmCA-4 and selected derivatives.

Analytical data of synthetic compounds

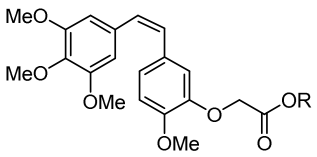

1 R=Et
2 R=H

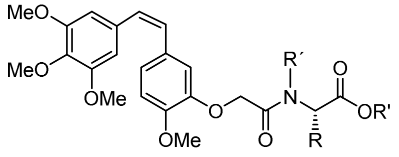

3.1 R=R'=H, R´´=Me
3.2 R=CH(CH$_3$)$_2$, R'=H, R´´=Me
3.3 R=CH$_2$CH(CH$_3$)$_2$, R'=H, R´´=Me
3.4 R=CH$_2$C$_6$H$_5$, R'=H, R´´=Me
3.5 R=R'=(CH$_2$)$_3$, R´´=Me
3.6 R=CH$_2$(OH), R'=H, R´´=Me
3.7 R=CH(OH)CH$_3$, R'=H, R´´=Me
3.8 R=CH$_2$pC$_6$H$_4$-OH, R'=H, R´´=Me
3.9 R=(CH$_2$)$_2$SCH$_3$, R'=H, R´´=Me
4.1 R=R'=R´´=H (71%)
4.2 R=CH(CH$_3$)$_2$, R'=R´´=H
4.3 R=CH$_2$CH(CH$_3$)$_2$, R'=R´´=H
4.4 R=CH$_2$C$_6$H$_5$, R'= R´´=H
4.5 R=R'=(CH$_2$)$_3$, R´´=H
4.6 R=CH$_2$(OH), R'=R´´=H
4.7 R=CH(OH)CH$_3$, R'=R´´=H
4.8 R=CH$_2$pC$_6$H$_4$-OH, R'=R´´=H
4.9 R=(CH$_2$)$_2$SCH$_3$, R'=R´´=H

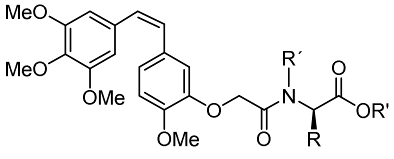

5.1 R=CH(CH$_3$)$_2$, R'=H, R´´=Me
5.2 R=CH$_2$CH(CH$_3$)$_2$, R'=H, R´´=Me
5.3 R=CH$_2$C$_6$H$_5$, R'=H, R´´=Me
5.4 R=R'=(CH$_2$)$_3$, R´´=Me
5.5 R=CH$_2$(OH), R'=H, R´´=Me
6.1 R=CH(CH$_3$)$_2$, R'=R´´=H
6.2 R=CH$_2$CH(CH$_3$)$_2$, R'=R´´=H
6.3 R=CH$_2$C$_6$H$_5$, R'=R´´=H
6.4 R=R'=(CH$_2$)$_3$, R´´=H
6.5 R=CH$_2$(OH), R'= R´´=H

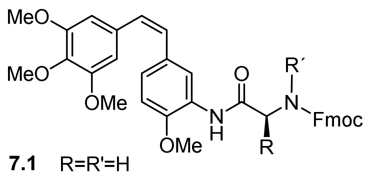

7.1 R=R'=H
7.2 R=CH$_3$, R'=H
7.3 R=CH(CH$_3$)$_2$, R'=H
7.4 R=CH$_2$CH(CH$_3$)$_2$, R'=H
7.5 R=CH$_2$C$_6$H$_5$, R'=H
7.6 R=R'=(CH$_2$)$_3$
7.7 R=CH(OH)CH$_3$, R'=H
7.8 R=CH$_2$p-C$_6$H$_4$-OH, R'=H
7.9 R=(CH$_2$)$_2$SCH$_3$, R'=H

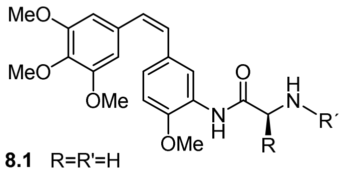

8.1 R=R'=H
8.2 R=CH$_3$, R'=H
8.3 R=CH(CH$_3$)$_2$, R'=H
8.4 R=CH$_2$CH(CH$_3$)$_2$, R'=H
8.5 R=CH$_2$C$_6$H$_5$, R'=H
8.6 R=R'=(CH$_2$)$_3$
8.7 R=CH(OH)CH$_3$, R'=H
8.8 R=CH$_2$p-C$_6$H$_4$-OH, R'=H
8.9 R=(CH$_2$)$_2$SCH$_3$, R'=H

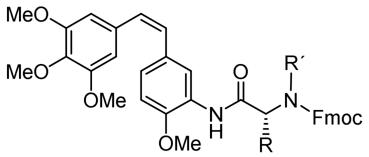

9.1 R=CH(CH$_3$)$_2$, R'=H
9.2 R=CH$_2$CH(CH$_3$)$_2$, R'=H
9.3 R=CH$_2$C$_6$H$_5$, R'=H
9.4 R=R'=(CH$_2$)$_3$

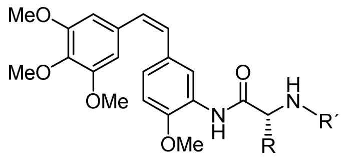

10.1 R=CH(CH$_3$)$_2$, R'=H
10.2 R=CH$_2$CH(CH$_3$)$_2$, R'=H
10.3 R=CH$_2$C$_6$H$_5$, R'=H
10.4 R=R'=(CH$_2$)$_3$

Signal assignments in the NMR spectra of molecules **1-2**, **3.1-3.9**, **4.1-4.9**, **5.1-5.5** and **6.1-6.5** having the general structures indicated below are based on the following, non-systematic numbering system:

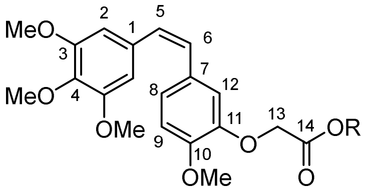

1-2

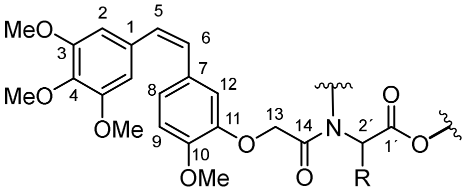

3.1-3.9, 4.1-4.9, 5.1-5.5 and 6.1-6.5

Ethyl (*Z*)-2-(2-methoxy-5-(3,4,5-trimethoxystyryl)phenoxy)acetate (1): yield, 94%, off-white solid m. p. 70-72 ºC; IR: ν_{max} 1755 cm^{-1} (br, C=O); ^{1}H NMR (500 MHz, $CDCl_3$) δ 6.90 (1H, dd, *J* = 8.3, 2 Hz; H-8), 6.78 (1H, d, *J* = 8.3 Hz; H-9), 6.74 (1H, d, *J* = 2 Hz; H-12), 6.47 (2H, s; H-2), 6.46 (1H, d, *J* = 12.2 Hz; H-5), 6.42 (1H, d, *J* = 12.2 Hz; H-6), 4.48 (2H, s; H-13), 4.18 (2H, q, *J* = 7.3 Hz; ethyl ester CH_2), 3.84 (3H, s; OMe), 3.82 (3H, s; OMe), 3.69 (6H, s; 2 OMe), 1.23 (3H, t, *J* = 7.3 Hz; ethyl ester CH_3); ^{13}C NMR (125 MHz, $CDCl_3$) δ 168.5 (C-14), 152.9 (x 2, C-3), 148.7 (C-11), 146.8 (C-10), 137.1 (C-4), 132.6 (C-1), 129.8 (C-7); 129.3, 129.1 (C-5, C-6), 123.2 (C-8), 114.6 (C-9), 111.6 (C-12), 106.0 (x 2, C-2); 66.1 (C-13), 61.1 (ethyl ester CH_2); 60.7 (OMe), 55.9 (OMe), 55.8 (2 x OMe), 14.0 (ethyl ester CH_3); HR ESMS *m/z* 425.1576 (M+Na)$^+$. Calc. for $C_{22}H_{26}NaO_7$, 425.1576.

(*Z*)-2-(2-Methoxy-5-(3,4,5-trimethoxystyryl)phenoxy)acetic acid (2): yield, 91%, off-white solid m. p. 109-111 ºC; IR: ν_{max} 3500-3200 (br, OH), 1742 (br, C=O) cm^{-1}; ^{1}H NMR (500 MHz, $CDCl_3$) δ 8.10 (1H, br s, OH), 6.93 (1H, dd, *J* = 8.3, 2 Hz; H-8), 6.79 (1H, d, *J* = 8.3 Hz; H-9), 6.78 (1H, d, *J* = 2 Hz; H-12), 6.47 (2H, s; H-2), 6.45 (2H, s; H-5, H-6), 4.52 (2H, s; H-13), 3.85 (3H, s; OMe), 3.83 (3H, s; OMe), 3.69 (6H, s; 2 x OMe); ^{13}C NMR (125 MHz, $CDCl_3$) δ 172.0 (C-14), 152.9 (x 2, C-3), 148.7 (C-11), 146.6 (C-10), 137.1 (C-4), 132.7 (C-1), 130.0 (C-7); 129.2, 129.1 (C-5, C-6), 123.8 (C-8), 115.2 (C-9), 111.7 (C-12), 106.0 (x 2, C-2); 66.3 (C-13); 60.8 (OMe), 55.9 (2 x OMe), 55.8 (OMe); HR ESMS *m/z* 373.1284 (M−H)$^-$. Calc. for $C_{20}H_{21}O_7$, 373.1287.

Methyl (*Z*)-(2-(2-methoxy-5-(3,4,5-trimethoxystyryl)phenoxy)acetyl)glycinate (3.1): yield, 68% off-white solid m. p. 103-105 ºC; IR: ν_{max} 3400 (br, NH), 1751, 1683 (C=O) cm^{-1}; ^{1}H NMR (500 MHz, $CDCl_3$) δ 7.45 (1H, br s, NH), 6.96 (1H, dd, *J* = 8.3, 2 Hz; H-8), 6.85 (1H, d, *J* = 2 Hz; H-12), 6.80 (1H, d, *J* = 8.3 Hz; H-9), 6.48 (2H, s; H-5, H-6), 6.46 (2H, s; H-2), 4.43 (2H, s; H-13), 4.11 (2H, d, *J* = 6 Hz; H-2´), 3.87 (3H, s; OMe), 3.84 (3H, s; OMe), 3.76 (3H, s; OMe), 3.69 (6H, s; 2 x OMe); ^{13}C NMR (125 MHz, $CDCl_3$) δ 169.7, 168.7 (C-14, C-1´), 153.0 (x 2, C-3), 148.9 (C-11), 146.7 (C-10), 137.4 (C-4), 132.5 (C-1), 130.3 (C-7); 129.5, 128.9 (C-5, C-6), 124.0 (C-8), 116.5 (C-9), 111.7 (C-12), 106.0 (x 2, C-2); 69.4 (C-13), 40.7 (C-2´); 60.9 (OMe), 55.9 (2 x OMe), 55.8 (OMe), 52.3 (OMe); HR ESMS *m/z* 468.1634 (M+Na)$^+$. Calc. for $C_{23}H_{27}NNaO_8$, 468.1634.

Methyl (*Z*)-(2-(2-methoxy-5-(3,4,5-trimethoxystyryl)phenoxy)acetyl)-L-valinate (3.2): yield, 72%, yellowish oil; $[\alpha]_D$ +11.8 (*c* 1.1, $CHCl_3$); IR: ν_{max} 3350 (br, NH), 1742, 1686 (C=O) cm^{-1}; ^{1}H NMR (500 MHz, $CDCl_3$) δ 7.50 (1H, br d, *J* ~ 9 Hz, NH), 6.98 (1H, dd, *J* = 8.3, 2 Hz; H-8), 6.87 (1H, d, *J* = 2 Hz; H-12), 6.80 (1H, d, *J* = 8.3 Hz; H-9), 6.49 (2H, s; H-2), 6.47 (2H, app s; H-5, H-6), 4.59 (1H, dd, *J* = 9, 5 Hz; H-2´), 4.46 (1H, d, *J* = 15 Hz; H-13a), 4.43 (1H, d, *J* = 15 Hz; H-13b), 3.87 (3H, s; OMe), 3.85

(3H, s; OMe), 3.73 (3H, s; OMe), 3.70 (6H, s; 2 x OMe), 2.20 (1H, m; valine isopropyl CH), 0.94 (3H, d, *J* = 7 Hz; valine isopropyl Me), 0.91 (3H, d, *J* = 7 Hz; valine isopropyl Me); ^{13}C NMR (125 MHz, $CDCl_3$) δ 171.8, 168.5 (C-14, C-1´), 153.0 (x 2, C-3), 149.0 (C-11), 146.9 (C-10), 137.4 (C-4), 132.5 (C-1), 130.4 (C-7); 129.5, 128.9 (C-5, C-6), 124.0 (C-8), 116.8 (C-9), 111.6 (C-12), 106.0 (x 2, C-2), 56.6 (C-2´), 31.2 (valine isopropyl CH); 69.8 (C-13); 60.9 (OMe), 55.9 (2 x OMe), 55.8 (OMe), 52.0 (OMe), 18.8, 17.6 (valine isopropyl Me); HR ESMS *m/z* 510.2099 $(M+Na)^+$. Calc. for $C_{26}H_{33}NNaO_8$, 510.2104.

Methyl (*Z*)-(2-(2-methoxy-5-(3,4,5-trimethoxystyryl)phenoxy)acetyl)-L-leucinate (3.3): yield, 79%, off-white solid m. p. 85-87 ºC; $[\alpha]_D$ +1.7 (*c* 1.2, $CHCl_3$); IR: ν_{max} 3350 (br, NH), 1744, 1684 (C=O) cm^{-1}; ^{1}H NMR (500 MHz, $CDCl_3$) δ 7.40 (1H, br d, *J* ~ 8.5 Hz, NH), 6.98 (1H, dd, *J* = 8.3, 2 Hz; H-8), 6.86 (1H, d, *J* = 2 Hz; H-12), 6.80 (1H, d, *J* = 8.3 Hz; H-9), 6.49 (2H, s; H-2), 6.47 (2H, app s; H-5, H-6), 4.68 (1H, m; H-2´), 4.43 (1H, d, *J* = 15 Hz; H-13a), 4.41 (1H, d, *J* = 15 Hz; H-13b), 3.87 (3H, s; OMe), 3.85 (3H, s; OMe), 3.73 (3H, s; OMe), 3.70 (6H, s; 2 x OMe), 1.75-1.55 (3H, br m; leucine $CHCH_2$), 0.94 (3H, d, *J* = 7 Hz; leucine isopropyl Me), 0.93 (3H, d, *J* = 7 Hz; leucine isopropyl Me); ^{13}C NMR (125 MHz, $CDCl_3$) δ 172.8, 168.4 (C-14, C-1´), 153.0 (x 2, C-3), 149.0 (C-11), 146.9 (C-10), 137.4 (C-4), 132.5 (C-1), 130.4 (C-7); 129.6, 128.9 (C-5, C-6), 124.0 (C-8), 117.0 (C-9), 111.6 (C-12), 106.0 (x 2, C-2), 50.3 (C-2´), 24.8 (leucine isopropyl CH); 69.8 (C-13), 41.5 (leucine CH_2); 60.9 (OMe), 55.9 (2 x OMe), 55.8 (OMe), 52.2 (OMe), 22.8, 21.8 (leucine isopropyl Me); HR ESMS *m/z* 524.2250 $(M+Na)^+$. Calc. for $C_{27}H_{35}NNaO_8$, 524.2260.

Methyl (*Z*)-(2-(2-methoxy-5-(3,4,5-trimethoxystyryl)phenoxy)acetyl)-L-phenylalaninate (3.4): yield, 80%, yellowish oil; $[\alpha]_D$ +24.7 (*c* 1.0, $CHCl_3$); IR: ν_{max} 3400, 3340 (br, NH), 1745, 1682 (C=O) cm^{-1}; ^{1}H NMR (500 MHz, $CDCl_3$) δ 7.55 (1H, br d, *J* ~ 8 Hz, NH), 7.25-7.20 (3H, m; phenylalanine aromatic protons), 7.08 (2H, m; phenylalanine aromatic protons), 6.98 (1H, dd, *J* = 8.3, 2 Hz; H-8), 6.83 (1H, d, *J* = 2 Hz; H-12), 6.77 (1H, d, *J* = 8.3 Hz; H-9), 6.49 (2H, s; H-2), 6.47 (2H, degenerate AB system, *J* ~ 12 Hz; H-5, H-6), 4.94 (1H, dt, *J* = 8, 6.5 Hz; H-2´), 4.42 (1H, d, *J* = 15 Hz; H-13a), 4.38 (1H, d, *J* = 15 Hz; H-13b), 3.85 (3H, s; OMe), 3.73 (3H, s; OMe), 3.71 (3H, s; OMe), 3.69 (6H, s; 2 x OMe), 3.12 (2H, m; phenylalanine CH_2); ^{13}C NMR (125 MHz, $CDCl_3$) δ 171.4, 168.3 (C-14, C-1´), 153.0 (x 2, C-3), 149.1 (C-11), 146.9 (C-10), 137.4 (C-4), 135.8 (phenylalanine aromatic), 132.6 (C-1), 130.3 (C-7); 129.5 (C-5 or C-6), 129.2 (x 2, phenylalanine aromatic), 128.9 (C-6 or C-5), 128.5 (x 2, phenylalanine aromatic), 127.0 (phenylalanine aromatic), 124.1 (C-8), 116.9 (C-9), 111.7 (C-12), 106.0 (x 2, C-2), 52.8 (C-2´); 69.8 (C-13), 38.1 (phenylalanine CH_2); 60.9 (OMe), 56.0 (2 x OMe), 55.7 (OMe), 52.2 (OMe); HR ESMS *m/z* 558.2100 $(M+Na)^+$. Calc. for $C_{30}H_{33}NNaO_8$, 558.2104.

Methyl (*Z*)-(2-(2-methoxy-5-(3,4,5-trimethoxystyryl)phenoxy)acetyl)-L-prolinate (3.5): yield, 74%, yellowish oil; $[\alpha]_D$ –41.9 (*c* 1.1, $CHCl_3$); IR: ν_{max} 1742, 1671 (C=O) cm^{-1}; ^{1}H NMR (500 MHz, $CDCl_3$) δ 6.90 (1H, dd, *J* = 8.3, 2 Hz; H-8), 6.80 (1H, d, *J* = 2 Hz; H-12), 6.76 (1H, d, *J* = 8.3 Hz; H-9), 6.50 (2H, s; H-2), 6.48 (1H, d, *J* = 12 Hz; H-5 or H-6), 6.43 (1H, d, *J* = 12 Hz; H-6 or H-5), 4.52 (2H, s; H-13), 4.49 (1H, m; H-2´), 3.83 (3H, s; OMe), 3.82 (3H, s; OMe), 3.71 (6H, s; 2 x OMe), 3.68 (3H, s; OMe), 3.60-3.50 (2H, m; proline CH_2N), 2.20-1.90 (4H, br m; proline 2 x CH_2); ^{13}C NMR (125 MHz, $CDCl_3$) δ 172.3, 166.4 (C-14, C-1´), 152.9 (x 2, C-3), 148.7 (C-11), 147.0 (C-10), 137.1 (C-4), 132.9 (C-1), 129.8 (C-7); 129.5, 128.8 (C-5, C-6), 123.1 (C-8), 114.7 (C-9), 111.6 (C-12), 106.0 (x 2, C-2), 59.1 (C-2´); 67.9 (C-13), 46.1, 28.6, 24.9 (proline 3 x CH_2); 60.8 (OMe), 55.9 (2 x OMe), 55.8 (OMe), 52.1 (OMe); HR ESMS *m/z* 508.1945 $(M+Na)^+$. Calc. for $C_{26}H_{31}NNaO_8$, 508.1947.

Methyl (*Z*)-(2-(2-methoxy-5-(3,4,5-trimethoxystyryl)phenoxy)acetyl)-L-serinate (3.6): yield, 97%, off-white solid, m. p. 65-67 ºC; $[\alpha]_D$ +14.4 (*c* 1.0, $CHCl_3$); IR: ν_{max} 3500-3300 (br, OH, NH), 1748, 1676 (C=O) cm^{-1}; ^1H NMR (500 MHz, $CDCl_3$) δ 7.90 (1H, br d, *J* ~ 7.5 Hz, NH), 6.96 (1H, dd, *J* = 8.3, 2 Hz, H-8), 6.86 (1H, d, *J* = 2 Hz; H-12), 6.80 (1H, d, *J* = 8.3 Hz, H-9), 6.48 (2H, s; H-2), 6.46 (2H, app s; H-5, H-6), 4.71 (1H, m, H-2´), 4.43 (2H, s; H-13), 4.00 (1H, dd, *J* = 11.2, 4 Hz; serine CH_2), 3.91 (1H, dd, *J* = 11.2, 3.5 Hz; serine CH_2), 3.86 (3H, s; OMe), 3.84 (3H, s; OMe), 3.79 (3H, s; OMe), 3.70 (6H, s; 2 x OMe), 2.70 (1H, br s, OH); ^{13}C NMR (125 MHz, $CDCl_3$) δ 170.4, 169.0 (C-14, C-1´), 153.0 (x 2, C-3), 149.1 (C-11), 146.9 (C-10), 137.3 (C-4), 132.6 (C-1), 130.4 (C-7); 129.6, 128.9 (C-5, C-6), 124.2 (C-8), 117.0 (C-9), 111.8 (C-12), 106.0 (x 2, C-2), 54.3 (C-2´), 69.8 (C-13), 63.2 (serine CH_2); 60.9 (OMe), 55.9 (3 x OMe), 52.6 (OMe); HR ESMS *m/z* 498.1743 $(M+Na)^+$. Calc. for $C_{24}H_{29}NNaO_9$, 498.1740.

Methyl (2-(2-methoxy-5-((*Z*)-3,4,5-trimethoxystyryl)phenoxy)acetyl)-L-threoninate (3.7): yield, 70%, yellowish oil; $[\alpha]_D$ +2.2 (*c* 1.0, $CHCl_3$); IR: ν_{max} 3500-3300 (br, OH, NH), 1748, 1671 (C=O) cm^{-1}; ^1H NMR (500 MHz, $CDCl_3$) δ 7.75 (1H, br d, *J* ~ 9 Hz, NH), 6.98 (1H, dd, *J* = 8.3, 2 Hz; H-8), 6.89 (1H, d, *J* = 2 Hz; H-12), 6.80 (1H, d, *J* = 8.3 Hz; H-9), 6.49 (2H, s; H-2), 6.46 (2H, app s; H-5, H-6), 4.64 (1H, dd, *J* = 9, 2.5 Hz; H-2´), 4.49 (2H, s; H-13), 4.37 (1H, br qd, *J* ~ 6.5, 2.5 Hz; threonine β-H), 3.86 (3H, s; OMe), 3.84 (3H, s; OMe), 3.75 (3H, s; OMe), 3.70 (6H, s; 2 x OMe), 2.30 (1H, br s, OH), 1.18 (3H, d, *J* = 6.5 Hz; threonine Me); ^{13}C NMR (125 MHz, $CDCl_3$) δ 170.9, 169.3 (C-14, C-1´), 153.0 (x 2; C-3), 149.2 (C-11), 147.1 (C-10), 137.3 (C-4), 132.6 (C-1), 130.4 (C-7), 129.6, 128.9 (C-5, C-6), 124.2 (C-8), 117.3 (C-9), 111.8 (C-12) 106.0 (x 2, C-2), 67.9 (C-2´), 56.8 (threonine β-C), 70.1 (C-13); 60.9 (OMe), 56.0 (3 x OMe), 52.5 (OMe), 19.8* (threonine Me) (the signal marked with * appears low and broad); HR ESMS *m/z* 512.1895 $(M+Na)^+$. Calc. for $C_{25}H_{31}NNaO_9$, 512.1897.

Methyl (*Z*)-(2-(2-methoxy-5-(3,4,5-trimethoxystyryl)phenoxy)acetyl)-L-tyrosinate (3.8): yield, 84%, yellowish oil; $[\alpha]_D$ +40.0 (*c* 1.0, $CHCl_3$); IR: ν_{max} 3400, 3340 (br, OH, NH), 1746, 1666 (C=O) cm^{-1}; ^1H NMR (500 MHz, $CDCl_3$) δ 7.45 (1H, br d, *J* ~ 8 Hz, NH), 6.98 (1H, dd, *J* = 8.3, 2 Hz; H-8), 6.90 (2H, app d, *J* = 8.3 Hz; tyrosine aromatic protons), 6.86 (1H, d, *J* = 2 Hz; H-12), 6.79 (1H, d, *J* = 8.3 Hz; H-9), 6.65 (2H, app d, *J* = 8.3 Hz; tyrosine aromatic protons), 6.50 (2H, s; H-2), 6.47 (2H, app s; H-5, H-6), 5.80 (1H, br s, OH), 4.90 (1H, dt, *J* = 8, 6 Hz; H-2´), 4.43 (1H, d, *J* = 15 Hz; H-13a), 4.35 (1H, d, *J* = 15 Hz; H-13b), 3.86 (3H, s; OMe), 3.77 (3H, s; OMe), 3.71 (3H, s; OMe), 3.69 (6H, s; 2 x OMe), 3.08 (1H, dd, *J* = 14, 6 Hz; tyrosine CH_2), 3.02 (1H, dd, *J* = 14, 6 Hz; tyrosine CH_2); ^{13}C NMR (125 MHz, $CDCl_3$) δ 171.5, 168.6 (C-14, C-1´), 155.4 (tyrosine aromatic carbon), 153.0 (x 2, C-3), 149.2 (C-11), 146.7 (C-10), 137.2 (C-4), 132.7 (x 2; C-1, C-7), 126.8 (tyrosine aromatic carbon), 130.2 (x 2, tyrosine aromatic carbons), 129.4 (C-5 or C-6), 128.9 (C-6 or C-5), 124.2 (C-8), 117.4 (C-9), 115.4 (x 2, tyrosine aromatic carbons), 111.7 (C-12), 106.1 (x 2, C-2), 53.0 (C-2´), 69.8 (C-13), 37.3 (tyrosine β-C), 60.9 (OMe), 56.0 (2 x OMe), 55.7 (OMe), 52.2 (OMe); HR ESMS *m/z* 574.2058 $(M+Na)^+$. Calc. for $C_{30}H_{33}NNaO_9$, 574.2053.

Methyl (*Z*)-(2-(2-methoxy-5-(3,4,5-trimethoxystyryl)phenoxy)acetyl)-L-methioninate (3.9): yield, 66%, off-white solid, m. p. 77-79 ºC; $[\alpha]_D$ +24.9 (*c* 1.0, $CHCl_3$); IR: ν_{max} 3350 (br, NH), 1741, 1683 (C=O) cm^{-1}; ^1H NMR (500 MHz, $CDCl_3$) δ 7.60 (1H, br d, *J* ~ 8 Hz, NH), 6.98 (1H, dd, *J* = 8.3, 2 Hz; H-8), 6.87 (1H, d, *J* = 2 Hz; H-12), 6.81 (1H, d, *J* = 8.3 Hz; H-9), 6.49 (2H, s; H-2), 6.47 (2H, degenerate AB system, *J* ~ 12 Hz; H-5, H-6), 4.78 (1H, dt, *J* = 8, 6 Hz; H-2´), 4.45 (1H, d, *J* = 15 Hz; H-13a), 4.43 (1H, d, *J* = 15 Hz; H-13b), 3.88 (3H, s; OMe), 3.85 (3H, s; OMe), 3.76 (3H, s; OMe), 3.71 (6H, s; 2 x

OMe), 2.50-2.45 (2H, m; CH_2S), 2.20 (1H, m; methionine β-H), 2.08 (3H, s; SMe), 2.02 (1H, m; methionine β-H); ^{13}C NMR (125 MHz, $CDCl_3$) δ 171.7, 168.4 (C-14, C-1´); 153.0 (x 2, C-3), 149.0 (C-11), 146.8 (C-10), 137.3 (C-4), 132.5 (C-1), 130.3 (C-7), 129.5, 128.8 (C-5, C-6), 124.1 (C-8), 116.9 (C-9), 111.6 (C-12), 106.0 (x 2, C-2), 50.9 (C-2´), 69.7 (C-13), 31.7 (methionine β-C), 29.7 (CH_2S); 60.9 (OMe), 55.9 (2 x OMe), 55.8 (OMe), 52.4 (OMe), 15.3 (MeS); HR ESMS *m/z* 542.1832 $(M+Na)^+$. Calc. for $C_{26}H_{33}NNaO_8S$, 542.1825.

(*Z*)-(2-(2-Methoxy-5-(3,4,5-trimethoxystyryl)phenoxy)acetyl)glycine (4.1): yield, 71%, off-white solid, m. p. 137-139 ºC; IR: ν_{max} 3500-3200 (br, OH, NH), 1735, 1655 (C=O) cm^{-1}; ^{1}H NMR (500 MHz, CD_3OD) δ 6.94 (1H, dd, *J* = 8.3, 2 Hz; H-8), 6.92 (1H, d, *J* = 8.3 Hz; H-9), 6.90 (1H, d, *J* = 2 Hz; H-12), 6.52 (2H, s; H-2), 6.51 (1H, d, *J* = 12.2 Hz; H-5 or H-6), 6.48 (1H, d, *J* = 12.2 Hz; H-6 or H-5), 4.38 (2H, s; H-13), 3.99 (2H, s; H-2´), 3.85 (3H, s; OMe), 3.75 (3H, s; OMe), 3.65 (6H, s; 2 x OMe) (NH and OH signals were not detected as they may be overlapping with the water signal at 4.80 ppm); ^{13}C NMR (125 MHz, CD_3OD) δ 172.5, 171.5 (C-14, C-1´); 154.3 (x 2; C-3), 150.6 (C-11), 148.4 (C-10), 138.4 (C-4), 134.5 (C-1), 131.8 (C-7), 130.5, 130.4 (C-5, C-6), 125.1 (C-8), 118.0 (C-9), 113.3 (C-12), 107.5 (x 2, C-2), 70.4 (C-13), 41.5 (C-2´), 61.2 (OMe), 56.5 (x 2, OMe), 55.8 (OMe); HR ESMS *m/z* 454.1477 $(M+Na)^+$. Calc. for $C_{22}H_{25}NNaO_8$, 454.1478.

(*Z*)-(2-(2-Methoxy-5-(3,4,5-trimethoxystyryl)phenoxy)acetyl)-L-valine (4.2): yield, 46%, off-white solid, m. p. 71-73 ºC; $[\alpha]_D$ +14.0 (*c* 1.0, $CHCl_3$); IR: ν_{max} 3500-3200 (br, OH, NH), 1736, 1677 (C=O) cm^{-1}; ^{1}H NMR (500 MHz, CD_3OD) δ 6.95-6.90 (3H, m; H-8, H-9, H-12), 6.52 (2H, s; H-2), 6.50 (1H, d, *J* = 12.2 Hz; H-5 or H-6), 6.47 (1H, d, *J* = 12.2 Hz; H-6 or H-5), 4.40 (3H, br s; H-13a, H-13b, H-2´), 3.84 (3H, s; OMe), 3.74 (3H, s; OMe), 3.64 (6H, s; 2 x OMe), 2.20 (1H, m; valine isopropyl CH), 0.94 (3H, d, *J* = 6.5 Hz; valine isopropyl Me), 0.92 (3H, d, *J* = 6.5 Hz; valine isopropyl Me) (NH and OH signals were not detected as they may be overlapping with the water signal at 4.80 ppm); ^{13}C NMR (125 MHz, CD_3OD) δ 174.5*, 170.9 (C-14, C-1´); 154.3 (x 2, C-3), 150.5 (C-11), 148.5 (C-10), 138.5 (C-4), 134.4 (C-1), 131.9 (C-7), 130.6, 130.3 (C-5, C-6), 125.2 (C-8), 118.1 (C-9), 113.2 (C-12), 107.5 (x 2, C-2), 58.5 (C-2´), 32.1 (valine isopropyl CH), 70.6 (C-13), 61.2 (OMe), 56.5 (OMe), 56.4 (2 x OMe), 19.5, 18.0 (valine isopropyl Me) (the signal marked with * appears low and broad); HR ESMS *m/z* 496.1945 $(M+Na)^+$. Calc. for $C_{25}H_{31}NNaO_8$, 496.1945.

(*Z*)-(2-(2-Methoxy-5-(3,4,5-trimethoxystyryl)phenoxy)acetyl)-L-leucine (4.3): yield, 52%; off-white solid, m. p. 108-110 ºC; $[\alpha]_D$ +1.0 (*c* 1.0, $CHCl_3$); IR: ν_{max} 3500-3200 (br, OH, NH), 1735, 1672 (C=O) cm^{-1}; ^{1}H NMR (500 MHz, CD_3OD) 6.95 (1H, dd, *J* = 8.3, 2 Hz; H-8), 6.92 (1H, d, *J* = 8.3 Hz; H-9), 6.90 (1H, d, *J* = 2 Hz; H-12), 6.52 (2H, s; H-2), 6.50 (1H, d, *J* = 12.2 Hz; H-5 or H-6), 6.47 (1H, d, *J* = 12.2 Hz; H-6 or H-5), 4.53 (1H, dd, *J* = 9, 5 Hz; H-2´), 4.41 (1H, d, *J* = 15.5 Hz; H-13a), 4.37 (1H, d, *J* = 15.5 Hz; H-13b), 3.86 (3H, s; OMe), 3.75 (3H, s; OMe), 3.66 (6H, s; 2 x OMe), 1.75-1.60 (3H, br m; leucine $CHCH_2$), 0.93 (3H, d, *J* = 6.5 Hz; leucine isopropyl Me), 0.91 (3H, d, *J* = 6.5 Hz; leucine isopropyl Me) (NH and OH signals are not detected as they may be overlapping with the water signal at 4.80 ppm): ^{13}C NMR (125 MHz, CD_3OD) δ 175.3, 171.0 (C-14, C-1´), 154.3 (x 2, C-3), 150.5 (C-11), 148.5 (C-10), 138.5 (C-4), 134.4 (C-1), 132.0 (C-7), 130.6, 130.3 (C-5, C-6), 125.1 (C-8), 118.1 (C-9), 113.3 (C-12), 107.5 (x 2, C-2), 51.6 (C-2´), 26.0 (leucine isopropyl CH), 70.6 (C-13), 41.9 (leucine isopropyl CH_2), 61.2 (OMe), 56.5 (OMe), 56.4 (2 x OMe), 23.3, 22.0 (leucine isopropyl Me); HR ESMS *m/z* 510.2103 $(M+Na)^+$. Calc. for $C_{26}H_{33}NNaO_8$, 510.2104.

(*Z*)-(2-(2-Methoxy-5-(3,4,5-trimethoxystyryl)phenoxy)acetyl)-L-phenylalanine (4.4): yield, 87%, yellowish oil; $[\alpha]_D$ +22.6 (*c* 1.1, $CHCl_3$); IR: ν_{max} 3500-3200 (br, OH, NH), 1741, 1675 (C=O) cm^{-1}; ^1H NMR (500 MHz, CD_3OD) δ 7.20-7.16 (3H, m; phenylalanine aromatic protons), 7.14-7.10 (2H, m; phenylalanine aromatic protons), 6.93 (1H, dd, *J* = 8.3, 2 Hz; H-8), 6.85 (1H, d, *J* = 8.3 Hz; H-9), 6.83 (1H, d, *J* = 2 Hz; H-12), 6.51 (2H, s; H-2), 6.47 (2H, app s; H-5, H-6), 4.76 (1H, dd, *J* = 7.3, 5.3 Hz; H-2´), 4.33 (1H, d, *J* = 15 Hz; H-13a), 4.26 (1H, d, *J* = 15 Hz; H-13b), 3.73 (3H, s; OMe), 3.69 (3H, s; OMe), 3.61 (6H, s; 2 x OMe), 3.18 (1H, dd, *J* = 14, 5.3 Hz; phenylalanine CH_2), 3.04 (1H, dd, *J* = 14, 7.3 Hz; phenylalanine CH_2) (NH and OH signals are not detected as they may be overlapping with the water signal at 4.80 ppm); ^{13}C NMR (125 MHz, CD_3OD) δ 173.7, 170.7 (C-14, C-1´), 154.3 (x 2, C-3), 150.5 (C-11), 148.5 (C-10), 138.4 (C-4), 137.7 (phenylalanine aromatic), 134.4 (C-1), 131.8 (C-7); 130.5 (C-5 or C-6), 130.4 (x 2, phenylalanine aromatic), 130.3 (C-6 or C-5), 129.5 (x 2, phenylalanine aromatic), 127.9 (phenylalanine aromatic), 125.3 (C-8), 118.2 (C-9), 113.2 (C-12), 107.5 (x 2, C-2), 54.3 (C-2´), 70.6 (C-13), 38.4 (phenylalanine CH_2); 61.2 (OMe), 56.5 (2 x OMe), 56.4 (OMe); HR ESMS *m/z* 520.1973 $(M-H)^-$. Calc. for $C_{29}H_{30}NO_8$, 520.1971.

(*Z*)-(2-(2-Methoxy-5-(3,4,5-trimethoxystyryl)phenoxy)acetyl)-L-proline (4.5): yield, 89%, off-white solid, m. p. 66-68 ºC; $[\alpha]_D$ −39.8 (*c* 1.1, $CHCl_3$); IR: ν_{max} 3500-3200 (br, OH), 1749, 1669 (C=O) cm^{-1}; ^1H NMR (500 MHz, CD_3OD) δ 6.90-6.80 (3H, m; H-8, H-9, H-12), 6.53 (2H, s; H-2), 6.48 (1H, d, *J* = 12.2 Hz; H-5 or H-6), 6.44 (1H, d, *J* = 12.2 Hz; H-6 or H-5), 4.51 (2H, s; H-13), 4.45 (1H, m; H-2´), 3.80 (3H, s; OMe), 3.74 (3H, s; OMe), 3.65 (6H, s; 2 x OMe), 3.50-3.40 (2H, br m; proline CH_2N), 2.25-2.15 (1H, m; proline CH_2), 2.00-1.85 (3H, br m; proline CH_2) (OH signal not detected as it may be overlapping with the water signal at 4.80 ppm); ^{13}C NMR (125 MHz, CD_3OD) δ 175.2, 169.0 (C-14, C-1´); 154.2 (x 2, C-3), 150.5 (C-11), 148.5 (C-10), 138.3 (C-4), 134.6 (C-1), 131.4 (C-7), 130.6, 130.1 (C-5, C-6), 124.5 (C-8), 116.3 (C-9), 113.3 (C-12), 107.4 (x 2, C-2), 60.4 (C-2´), 68.6 (C-13), 47.1, 29.8, 25.7 (proline 3 x CH_2); 61.2 (OMe), 56.5 (OMe), 56.4 (2 x OMe); HR ESMS *m/z* 470.1818 $(M-H)^-$. Calc. for $C_{25}H_{28}NO_8$, 470.1815.

(*Z*)-(2-(2-Methoxy-5-(3,4,5-trimethoxystyryl)phenoxy)acetyl)-L-serine (4.6): yield, 65%, off-white solid, m. p. 125-128 ºC; $[\alpha]_D$ −9.8 (*c* 1.0, $CHCl_3$); IR: ν_{max} 3500-3200 (br, OH, NH), 1740, 1664 (C=O) cm^{-1}; ^1H NMR (500 MHz, CD_3OD) δ 6.94 (1H, dd, *J* = 8.3, 2 Hz; H-8), 6.92 (1H, d, *J* = 2 Hz, H-12), 6.91 (1H, d, *J* = 8.3 Hz; H-9), 6.52 (2H, s; H-2), 6.51 (1H, d, *J* = 12 Hz; H-5 or H-6), 6.47 (1H, d, *J* = 12 Hz; H-6 or H-5), 4.51 (1H, br t, *J* ~ 4 Hz; H-2´), 4.41 (1H, d, *J* = 15 Hz; H-13a), 4.37 (1H, d, *J* = 15 Hz; H-13b), 3.98 (1H, dd, *J* = 11.2, 4 Hz; serine CH_2), 3.85 (1H, dd, overlapped; serine CH_2), 3.84 (3H, s; OMe), 3.74 (3H, s; OMe), 3.65 (6H, s; 2 x OMe) (NH and two OH signals are not detected as they may be overlapping with the water signal at 4.80 ppm); ^{13}C NMR (125 MHz, CD_3OD) δ 173.3*, 171.0 (C-14, C-1´); 154.3 (x 2, C-3), 150.7 (C-11), 148.5 (C-10), 138.4 (C-4), 134.4 (C-1), 131.8 (C-7), 130.5, 130.3 (C-5, C-6), 125.2 (C-8), 118.3 (C-9), 113.2 (C-12), 107.5 (x 2, C-2), 55.8* (serine α-C); 70.7 (C-13), 62.9 (serine CH_2); 61.2 (OMe), 56.5 (3 x OMe) (the signals marked with * appear low and broad); HR ESMS *m/z* 484.1584 $(M+Na)^+$. Calc. for $C_{23}H_{27}NNaO_9$, 484.1577.

(2-(2-Methoxy-5-((*Z*)-3,4,5-trimethoxystyryl)phenoxy)acetyl)-L-threonine (4.7): yield, 68%, yellowish oil; $[\alpha]_D$ +5.2 (*c* 1.1, $CHCl_3$); IR: ν_{max} 3500-3200 (br, OH, NH), 1739, 1664 (C=O) cm^{-1}; ^1H NMR (500 MHz, CD_3OD) δ 6.94 (1H, dd, *J* = 8.3, 2 Hz; H-8), 6.92 (1H, d, *J* = 2 Hz; H-12), 6.90 (1H, d, *J* = 8.3 Hz; H-9), 6.52 (2H, s; H-2), 6.49 (1H, d, *J* = 12.2 Hz; H-5 or H-6), 6.47 (1H, d, *J* = 12.2 Hz;

H-6 or H-5), 4.46 (1H, br d, *J* ~ 3 Hz; H-2´), 4.45 (1H, d, *J* = 15 Hz; H-13a), 4.40 (1H, d, *J* = 15 Hz; H-13b), 4.37 (1H, qd, *J* = 7, 3 Hz; threonine β-H), 3.84 (3H, s; OMe), 3.74 (3H, s; OMe), 3.64 (6H, s; 2 x OMe), 1.16 (3H, d, *J* = 7 Hz; threonine Me) (NH and two OH signals are not detected as they may be overlapping with the water signal at 4.80 ppm); ^{13}C NMR (125 MHz, CD_3OD) δ 173.4*, 171.5 (C-14, C-1´), 154.3 (x 2, C-3), 150.7 (C-11), 148.5 (C-10), 138.4 (C-4), 134.4 (C-1), 131.7 (C-7), 130.5, 130.3 (C-5, C-6), 125.3 (C-8), 118.4 (C-9), 113.2 (C-12), 107.5 (x 2, C-2), 68.3 (C-2´), 58.6 (threonine β-C); 70.8 (C-13), 61.2 (OMe), 56.6 (OMe), 56.5 (2 x OMe), 20.5 (threonine Me) (the signal marked with * appears low and broad); HR ESMS *m/z* 474.1766 $(M-H)^-$. Calc. for $C_{24}H_{28}NO_9$, 474.1764.

(*Z*)-(2-(2-Methoxy-5-(3,4,5-trimethoxystyryl)phenoxy)acetyl)-L-tyrosine (4.8): yield, 97%, off-white solid, m. p. 71-73 ºC; $[\alpha]_D$ +31.5 (*c* 1.0, $CHCl_3$); IR: ν_{max} 3500-3200 (br, OH, NH), 1735, 1662 (C=O) cm^{-1}; ^{1}H NMR (500 MHz, CD_3OD) δ 6.95-6.90 (3H, m; H-8 and two tyrosine aromatic protons), 6.86 (1H, d, *J* = 8.3 Hz; H-9), 6.83 (1H, d, *J* = 2 Hz; H-12), 6.63 (2H, d, *J* = 8.4 Hz, tyrosine aromatic protons), 6.51 (2H, s; H-2), 6.48 (2H, app s; H-5 and H-6), 4.68 (1H, dd, *J* = 7, 5.3 Hz; H-2´), 4.34 (1H, d, *J* = 15 Hz; H-13a), 4.26 (1H, d, *J* = 15 Hz; H-13b), 3.74 (3H, s, OMe), 3.72 (3H, s, OMe), 3.62 (6H, s, 2 x OMe), 3.08 (1H, dd, *J* = 14, 5.3 Hz; tyrosine CH_2), 2.96 (1H, dd, *J* = 14, 7 Hz; tyrosine CH_2) (NH and two OH signals are not detected as they may be overlapping with the water signal at 4.80 ppm); ^{13}C NMR (125 MHz, CD_3OD) δ 174.5*, 170.7 (C-14, C-1´), 157.3 (tyrosine aromatic carbon), 154.3 (x 2, C-3), 150.5 (C-11), 148.4 (C-10), 138.4 (C-4), 134.5 (C-1), 131.8 (C-7), 128.4 (tyrosine aromatic carbon); 131.4 (x 2, tyrosine aromatic carbons), 130.5, 130.3 (C-5, C-6), 125.3 (C-8), 118.1 (C-9), 116.2 (x 2, tyrosine aromatic carbons), 113.2 (C-12), 107.5 (x 2, C-2), 54.8* (C-2´), 70.6 (C-13), 37.7 (tyrosine CH_2); 61.2 (OMe), 56.5 (2 x OMe), 56.4 (OMe) (the signals marked with * appear low and broad); HR ESMS *m/z* 536.1918 $(M-H)^-$. Calc. for $C_{29}H_{30}NO_9$, 536.1921.

(*Z*)-(2-(2-Methoxy-5-(3,4,5-trimethoxystyryl)phenoxy)acetyl)-L-methionine (4.9): yield, 76%, off-white solid, m. p. 103-105 ºC; $[\alpha]_D$ +17.0 (*c* 1.1, $CHCl_3$); IR: ν_{max} 3500-3200 (br, OH, NH), 1735, 1678 (C=O) cm^{-1}; ^{1}H NMR (500 MHz, CD_3OD) δ 6.94 (1H, dd, *J* = 8.3, 2 Hz; H-8), 6.92 (1H, d, *J* = 2 Hz; H-12), 6.90 (1H, d, *J* = 8.3 Hz; H-9), 6.52 (2H, s, H-2), 6.50 (1H, d, *J* = 12.2 Hz; H-5 or H-6), 6.48 (1H, d, *J* = 12.2 Hz; H-6 or H-5), 4.63 (1H, dd, *J* = 8.3, 5 Hz; H-2´), 4.41 (1H, d, *J* = 15 Hz; H-13a), 4.38 (1H, d, *J* = 15 Hz; H-13b), 3.85 (3H, s; OMe), 3.74 (3H, s; OMe), 3.65 (6H, s; 2 x OMe), 2.50-2.40 (2H, br m; CH_2S), 2.20-2.15 (1H, m; methionine β-H), 2.05 (3H, s; methionine Me), 2.05-1.95 (1H, m; methionine β-H) (NH and OH signals are not detected as they may be overlapping with the water signal at 4.80 ppm); ^{13}C NMR (125 MHz, CD_3OD) δ 174.3, 171.1 (C-14, C-1´), 154.3 (x 2, C-3), 150.5 (C-11), 148.5 (C-10), 138.4 (C-4), 134.4 (C-1), 131.9 (C-7), 130.6, 130.3 (C-5, C-6), 125.2 (C-8), 118.1 (C-9), 113.3 (C-12), 107.5 (x 2, C-2), 52.3 (C-2´), 70.6 (C-13), 32.2, 31.0 (2 x methionine CH_2), 61.2 (OMe), 56.6 (OMe), 56.5 (2 x OMe), 15.2 (methionine Me); HR ESMS *m/z* 528.1676 $(M+Na)^+$. Calc. for $C_{25}H_{31}NNaO_8S$, 528.1668.

Methyl (*Z*)-(2-(2-methoxy-5-(3,4,5-trimethoxystyryl)phenoxy)acetyl)-D-valinate (5.1): yield, 92%, yellowish oil; $[\alpha]_D$ −11.5 (*c* 1.1, $CHCl_3$); IR, ^{1}H NMR, ^{13}C NMR and HR ESMS data identical to compound **3.2**; HR ESMS *m/z* 510.2099 $(M+Na)^+$. Calc. for $C_{26}H_{33}NNaO_8$, 510.2104.

Methyl (*Z*)-(2-(2-methoxy-5-(3,4,5-trimethoxystyryl)phenoxy)acetyl)-D-leucinate (5.2): yield, 87%, off-white solid, m. p. 85-87 ºC; $[\alpha]_D$ −1.4 (*c* 1.2, $CHCl_3$); IR, ^1H NMR, ^{13}C NMR and HR ESMS data identical to compound **3.3**.

Methyl (*Z*)-(2-(2-methoxy-5-(3,4,5-trimethoxystyryl)phenoxy)acetyl)-D-phenylalaninate (5.3): yield, 38%, yellowish oil; $[\alpha]_D$ −25.3 (*c* 1.0, $CHCl_3$); IR, ^1H NMR, ^{13}C NMR and HR ESMS data identical to compound **3.4**.

Methyl (*Z*)-(2-(2-methoxy-5-(3,4,5-trimethoxystyryl)phenoxy)acetyl)-D-prolinate (5.4): yield, 68%, yellowish oil; $[\alpha]_D$ +36.9 (*c* 1.1, $CHCl_3$); IR, ^1H NMR, ^{13}C NMR and HR ESMS data identical to compound **3.5**.

Methyl (*Z*)-(2-(2-methoxy-5-(3,4,5-trimethoxystyryl)phenoxy)acetyl)-D-serinate (5.5): yield, 70%, off-white solid, m. p. 83-85 ºC; $[\alpha]_D$ −16.4 (*c* 1.0, $CHCl_3$); IR, ^1H NMR, ^{13}C NMR and HR ESMS data identical to compound **3.6**.

(*Z*)-(2-(2-Methoxy-5-(3,4,5-trimethoxystyryl)phenoxy)acetyl)-D-valine (6.1): yield, 50%, off-white solid m. p. 71-73 ºC; $[\alpha]_D$ −14.6 (*c* 1.0, $CHCl_3$); IR, ^1H NMR, ^{13}C NMR and HR ESMS data identical to compound **4.2**.

(*Z*)-(2-(2-Methoxy-5-(3,4,5-trimethoxystyryl)phenoxy)acetyl)-D-leucine (6.2): yield, 91%, off-white solid, m. p. 105-107 ºC; $[\alpha]_D$ −0.9 (*c* 1.0, $CHCl_3$); IR, ^1H NMR, ^{13}C NMR and HR ESMS data identical to compound **4.3**.

(*Z*)-(2-(2-Methoxy-5-(3,4,5-trimethoxystyryl)phenoxy)acetyl)-D-phenylalanine (6.3): yield, 68%, yellowish oil; $[\alpha]_D$ −22.8 (*c* 1.1, $CHCl_3$); IR, ^1H NMR, ^{13}C NMR and HR ESMS data identical to compound **4.4**.

(*Z*)-(2-(2-Methoxy-5-(3,4,5-trimethoxystyryl)phenoxy)acetyl)-D-proline (6.4): yield, 85%, off-white solid, m. p. 67-69 ºC; $[\alpha]_D$ +46.4 (*c* 1.1, $CHCl_3$); IR, ^1H NMR, ^{13}C NMR and HR ESMS data identical to compound **4.5**.

(*Z*)-(2-(2-Methoxy-5-(3,4,5-trimethoxystyryl)phenoxy)acetyl)-D-serine (6.5): yield, 67%, off-white solid, m. p. 135-137 ºC; $[\alpha]_D$ +12.2 (*c* 1.1, $CHCl_3$); IR, ^1H NMR, ^{13}C NMR and HR ESMS data identical to compound **4.6**.

Signal assignments in the NMR spectra of molecules **7.1-7.9**, **8.1-8.9**, **9.1-9.4** and **10.1-10.4** having the general structures indicated below are based on the following, non-systematic numbering system:

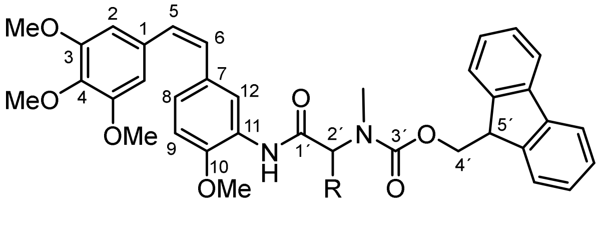

7.1-7.9 and **9.1-9.4**

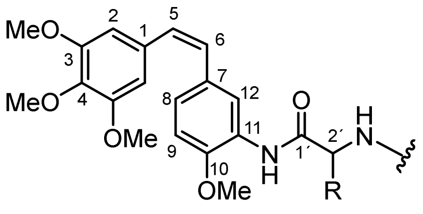

8.1-8.9 and **10.1-10.4**

(9*H*-Fluoren-9-yl)methyl (*Z*)-(2-((2-methoxy-5-(3,4,5-trimethoxystyryl)phenyl)amino)-2-oxoethyl) carbamate (7.1): yield, 72%, off-white solid, m. p.78-80 ºC; IR: ν_{max} 3400, 3300 (br NH), 1727, 1686 (C=O) cm^{-1}; ^1H NMR (500 MHz, $CDCl_3$) δ 8.30 (1H, br s; H-12), 8.14 (1H, br s, NH), 7.77 (2H, d, *J* ~ 7.5 Hz; fluorenyl aromatic protons), 7.62 (2H, br d, *J* ~ 7 Hz; fluorenyl aromatic protons), 7.40 (2H, br t, *J* ~ 7 Hz; fluorenyl aromatic protons), 7.32 (2H, m; fluorenyl aromatic protons), 7.02 (1H, d, *J* ~ 8.3 Hz; H-8), 6.71 (1H, d, *J* = 8.8 Hz; H-9), 6.53 (1H, d, *J* = 12.2 Hz; H-5 or H-6), 6.52 (2H, s; H-2), 6.45 (1H, d, *J* = 12.2 Hz; H-6 or H-5), 5.60 (1H, br s, NH), 4.46 (2H, d, *J* = 7.3 Hz; H-4´), 4.26 (1H, t, *J* = 7.3 Hz; H-5´), 4.04 (2H, br s; H-2´), 3.85 (3H, s; OMe), 3.79 (3H, s; OMe), 3.69 (6H, s; OMe); ^{13}C NMR (125 MHz, $CDCl_3$) δ 166.6 (C-1´), 156.6* (C-3´), 152.8 (x 2, C-3), 147.1 (C-10), 143.7 (x 2, fluorenyl aromatic carbons), 141.3 (x 2, fluorenyl aromatic carbons), 137.1 (C-4), 132.8 (C-1), 130.2 (C-7), 126.7 (C-11); 129.5, 129.3 (C-5, C-6), 127.7 (x 2, fluorenyl aromatic carbons), 127.1 (x 2, fluorenyl aromatic carbons), 125.0 (x 2, fluorenyl aromatic carbons), 124.7 (C-8), 120.8 (C-9), 120.0 (x 2, fluorenyl aromatic carbons), 109.6 (C-12), 106.1 (x 2, C-2), 47.1 (C-5´), 67.4 (C-4´), 45.4* (C-2´), 60.8 (OMe), 55.9 (2 x OMe), 55.8 (OMe) (the signals marked with * appear low and broad); HR ESMS *m/z* 617.2262 $(M+Na)^+$. Calc. for $C_{35}H_{34}N_2NaO_7$, 617.2264.

(9*H*-Fluoren-9-yl)methyl (*S*,*Z*)-(1-((2-methoxy-5-(3,4,5-trimethoxystyryl)phenyl)amino)-1-oxo-propan-2-yl)carbamate (7.2): yield, 51%; off-white solid, m. p.143-145 ºC; $[\alpha]_D$ −29.2 (*c* 1.0, $CHCl_3$); IR: ν_{max} 3400, 3300 (br NH), 1701, 1686 (C=O) cm^{-1}; ^1H NMR (500 MHz, $CDCl_3$) δ 8.33 (1H, br d, *J* ~ 1.5 Hz; H-12), 8.25 (1H, br s, NH), 7.77 (2H, d, *J* ~ 7.8 Hz; fluorenyl aromatic protons), 7.60 (2H, br d, *J* ~ 7 Hz; fluorenyl aromatic protons), 7.40 (2H, br t, *J* ~ 7 Hz; fluorenyl aromatic protons), 7.31 (2H, m; fluorenyl aromatic protons), 7.02 (1H, dd, *J* = 8.3, 1.5 Hz; H-8), 6.71 (1H, d, *J* = 8.3 Hz; H-9), 6.52 (2H, s; H-2), 6.51 (1H, d, *J* = 12.2 Hz; H-5 or H-6), 6.45 (1H, d, *J* = 12.2 Hz; H-6 or H-5), 5.40 (1H, br s, NH), 4.50-4.40 (3H, m; H-2´, H-4´), 4.25 (1H, t, *J* = 7 Hz; H-5´), 3.85 (3H, s; OMe), 3.80 (3H, s; OMe), 3.70 (6H, s; 2 x OMe), 1.49 (3H, d, *J* = 7.3 Hz; alanine Me); ^{13}C NMR (125 MHz, $CDCl_3$) δ 170.1 (C-1´), 156.0* (C-3´); 152.8 (x 2, C-3), 147.1 (C-10), 143.7 (x 2, fluorenyl aromatic carbons), 141.3 (x 2, fluorenyl aromatic carbons), 137.1 (C-4), 132.8 (C-1), 130.1 (C-7), 126.9 (C-11), 129.5, 129.2 (C-5, C-6), 127.7 (x 2, fluorenyl aromatic carbons), 127.1 (x 2, fluorenyl aromatic carbons), 125.0 (x 2, fluorenyl aromatic carbons), 124.7 (C-8), 120.8 (C-9), 120.0 (x 2, fluorenyl aromatic carbons), 109.6 (C-12), 106.1 (x 2, C-2), 51.5* (C-2´), 47.1 (C-5´), 67.1 (C-4´), 60.8 (OMe), 55.9 (2 x OMe), 55.8 (OMe), 18.6 (alanine Me) (the signals marked with * appear low and broad); HR ESMS *m/z* 609.2600 $(M+H)^+$. Calc. for $C_{36}H_{37}N_2O_7$, 609.2607.

(9*H*-Fluoren-9-yl)methyl (*S*,*Z*)-(1-((2-methoxy-5-(3,4,5-trimethoxystyryl)phenyl)amino)-3-methyl-1-oxobutan-2-yl)carbamate (7.3): yield, 61%, off-white solid, m. p.176-178 ºC; $[\alpha]_D$ −35.3 (*c* 1.0, $CHCl_3$); IR: ν_{max} 3400, 3300 (br NH), 1714, 1683 (C=O) cm^{-1}; ^1H NMR (500 MHz, $CDCl_3$) δ 8.34 (1H, br d , *J* ~ 1.5 Hz; H-12), 8.00 (1H, br s, NH), 7.77 (2H, d, *J* ~ 7.5 Hz; fluorenyl aromatic protons), 7.60 (2H, br d, *J* ~ 6 Hz; fluorenyl aromatic protons), 7.40 (2H, br t, *J* ~ 7 Hz; fluorenyl aromatic protons), 7.32 (2H, m; fluorenyl aromatic protons), 7.03 (1H, dd, *J* = 8.3, 1.5 Hz; H-8), 6.72 (1H, d, *J* = 8.3 Hz; H-9), 6.53 (2H, s; H-2), 6.51 (1H, d, *J* = 12.2 Hz; H-5 or H-6), 6.46 (1H, d, *J* = 12.2 Hz; H-6 or H-5), 5.45 (1H, br s, NH), 4.50-4.40 (2H, m; H-4´), 4.24 (1H, t, *J* = 7 Hz; H-5´), 4.14 (1H, m; H-2´), 3.85 (3H, s; OMe), 3.83 (3H, br s; OMe), 3.70 (6H, s; 2 x OMe), 2.22 (1H, dqq, *J* = 7, 7, 7 Hz; isopropyl CH), 1.02 (6H, app t, *J* ~ 7 Hz; 2 x isopropyl Me); ^{13}C NMR (125 MHz, $CDCl_3$) δ 169.2 (C-1´), 156.4 (C-3´), 152.8 (x 2, C-3), 147.2 (C-10), 143.7 (x 2, fluorenyl aromatic carbons), 141.2 (x 2, fluorenyl aromatic carbons), 137.1 (C-4), 132.7 (C-1), 130.1 (C-7), 126.7 (C-11), 129.5, 129.2, 127.6 (x 2, fluorenyl aromatic carbons), 127.0 (x 2, fluorenyl aromatic carbons), 124.9 (x 2, fluorenyl aromatic carbons), 124.7 (C-8), 120.8 (C-9), 119.8 (x 2, fluorenyl aromatic carbons), 109.6 (C-12), 106.0 (x 2, C-2), 61.3 (C-2´), 47.1 (C-5´), 31.2 (isopropyl CH), 67.1 (C-4´), 60.8 (OMe), 55.9 (2 x OMe), 55.8 (OMe), 19.1, 17.8 (2 x isopropyl Me); HR ESMS *m/z* 659.2725 $(M+Na)^+$. Calc. for $C_{38}H_{40}N_2NaO_7$, 659.2733.

(9*H*-Fluoren-9-yl)methyl (*S*,*Z*)-(1-((2-methoxy-5-(3,4,5-trimethoxystyryl)phenyl)amino)-4-methyl-1-oxopentan-2-yl)carbamate (7.4): yield, 61%, off-white solid, m. p.72-74 ºC; $[\alpha]_D$ −34.3 (*c* 1.0, $CHCl_3$); IR: ν_{max} 3400, 3320 (br NH), 1701, 1685 (C=O) cm^{-1}; ^1H NMR (500 MHz, $CDCl_3$) δ 8.34 (1H, br d, *J* ~ 1.5 Hz; H-12), 8.20 (1H, br s, NH), 7.77 (2H, br d, *J* ~ 7 Hz; fluorenyl aromatic protons), 7.60 (2H, br d, *J* ~ 7 Hz; fluorenyl aromatic protons), 7.40 (2H, br t, *J* ~ 7 Hz; fluorenyl aromatic protons), 7.31 (2H, m; fluorenyl aromatic protons), 7.02 (1H, dd, *J* = 8.3, 1.5 Hz; H-8), 6.70 (1H, d, *J* = 8.3 Hz; H-9), 6.53 (2H, s; H-2), 6.51 (1H, d, *J* = 12.2 Hz; H-5 or H-6), 6.45 (1H, d, *J* = 12.2 Hz; H-6 or H-5), 5.25 (1H, br s, NH), 4.44 (2H, d, *J* = 7 Hz; H-4´), 4.34 (1H, m; H-2´), 4.24 (1H, t, *J* = 7 Hz; H-5´), 3.84 (3H, s; OMe), 3.81 (3H, br s; OMe), 3.69 (6H, s; 2 x OMe), 1.80-1.70 (2H, m; leucine CH_2), 1.60 (1H, m; leucine CH), 0.99 (6H, br d, *J* ~ 7 Hz; 2 x leucine Me); ^{13}C NMR (125 MHz, $CDCl_3$) δ 170.1 (C-1´), 156.2 (C-3´), 152.8 (x 2, C-3), 147.2 (C-10), 143.7 (x 2, fluorenyl aromatic carbons), 141.2 (x 2, fluorenyl aromatic carbons), 137.1 (C-4), 132.7 (C-1), 130.1 (C-7), 126.9 (C-11), 129.5, 129.2 (C-5, C-6), 127.6 (x 2, fluorenyl aromatic carbons), 127.0 (x 2, fluorenyl aromatic carbons), 124.9 (x 2, fluorenyl aromatic carbons), 124.6 (C-8), 120.9 (C-9), 119.8 (x 2, fluorenyl aromatic carbons), 109.6 (C-12), 106.0 (x 2, C-2), 54.5 (C-2´), 47.1 (C-5´), 22.1 (isopropyl CH), 67.1 (C-4´), 41.6 (leucine CH_2), 60.8 (OMe), 55.9 (2 x OMe), 55.8 (OMe), 24.7, 22.8 (2 x isopropyl Me); HR ESMS *m/z* 673.2886 $(M+Na)^+$. Calc. for $C_{39}H_{42}N_2NaO_7$, 673.2890.

(9*H*-Fluoren-9-yl)methyl (*S*,*Z*)-(1-((2-methoxy-5-(3,4,5-trimethoxystyryl)phenyl)amino)-1-oxo-3-phenylpropan-2-yl)carbamate (7.5): yield, 59%, off-white solid, m. p.78-80 ºC; $[\alpha]_D$ −25.0 (*c* 1.0, $CHCl_3$); IR: ν_{max} 3400, 3300 (br NH), 1701, 1684 (C=O) cm^{-1}; ^1H NMR (500 MHz, $CDCl_3$) δ 8.32 (1H, br d, *J* ~ 1.5 Hz; H-12), 7.95 (1H, br s, NH), 7.77 (2H, br d, *J* ~ 7.3 Hz; fluorenyl aromatic protons), 7.56 (2H, app t, *J* ~ 7 Hz; fluorenyl aromatic protons), 7.40 (2H, br t, *J* ~ 7.3 Hz; fluorenyl aromatic protons), 7.35-7.20 (7H, br m; 2 x fluorenyl aromatic protons + 5 x phenylalanine aromatic protons), 7.01 (1H, dd, *J* = 8.8, 1.5 Hz; H-8), 6.66 (1H, d, *J* = 8.8 Hz; H-9), 6.53 (2H, s; H-2), 6.52 (1H, d, *J* = 12.2 Hz; H-5 or H-6), 6.47 (1H, d, *J* = 12.2 Hz; H-6 or H-5), 5.60 (1H, br s, NH), 4.60 (1H, br s; H-2´),

4.50-4.35 (2H, m; H-4´), 4.22 (1H, t, *J* = 7 Hz; H-5´), 3.86 (3H, s; OMe), 3.70 (6H, br s; 2 x OMe), 3.68 (3H, s; OMe), 3.25-3.10 (2H, br m; phenylalanine CH_2); ^{13}C NMR (125 MHz, $CDCl_3$) δ 168.6 (C-1´), 155.9* (C-3´), 152.8 (x 2, C-3), 147.5 (C-10), 143.7 (x 2, fluorenyl aromatic carbons), 141.2 (x 2, fluorenyl aromatic carbons), 137.1 (C-4), 136.2* (phenylalanine carbon), 132.7 (C-1), 130.1 (C-7), 126.6* (C-11), 129.5, 129.3 (C-5, C-6), 129.2 (x 3, three phenylalanine carbons), 128.7 (x 2, two phenylalanine carbons), 127.7 (x 2, fluorenyl aromatic carbons), 127.0 (x 2, fluorenyl aromatic carbons), 125.0 (x 2, fluorenyl aromatic carbons), 124.7 (C-8), 120.8 (C-9), 120.0 (x 2, fluorenyl aromatic carbons), 109.5 (C-12), 106.1 (x 2, C-2), 57.1* (C-2´), 47.1 (C-5´), 67.2 (C-4´), 38.7 (phenylalanine CH_2), 60.8 (OMe), 55.9 (2 x OMe), 55.8 (OMe) (the signals marked with * appear low and broad); HR ESMS *m/z* 685.2906 $(M+H)^+$. Calc. for $C_{42}H_{41}N_2O_7$, 685.2914.

(9*H*-Fluoren-9-yl)methyl (*S*,*Z*)-2-((2-methoxy-5-(3,4,5-trimethoxystyryl)phenyl)carbamoyl)-pyrrolidine-1-carboxylate (7.6): yield, 71%, off-white solid, m. p.70-72 ºC; $[\alpha]_D$ −73.3 (*c* 1.0, $CHCl_3$); IR: ν_{max} 3400, 3300 (br NH), 1700 (br C=O) cm^{-1}; ^{1}H NMR (500 MHz, $CDCl_3$) δ 8.90 (1H, br s, NH), 8.38 (1H, br s; H-12), 7.80-7.20 (8H, br m; fluorenyl aromatic protons), 7.10-7.00 (1H, br m; H-8), 6.67 (1H, d, *J* = 8.3 Hz; H-9), 6.55 (2H, s; H-2), 6.55-6.40 (2H, br m; H-5, H-6), 4.60-4.40 (4H, br m, H-2´, H-4´, H-5´), 3.85 (6H, s; 2 x OMe), 3.73 (3H, br s; OMe), 3.68 (3H, s; OMe), 3.65-3.60 (2H, br m; CH_2N), 2.45-2.00 (4H, br m; proline ring protons); ^{13}C NMR (125 MHz, $CDCl_3$) δ 169.4* (C-1´), 156.0* (C-3´); 152.8 (x 2, C-3), 147.2 (C-10), 143.7 (x 2, fluorenyl aromatic carbons), 141.2 (x 2, fluorenyl aromatic carbons), 137.2 (C-4), 132.8 (C-1), 130.0 (C-7), 126.9* (C-11), 129.6, 129.0 (C-5, C-6), 127.7 (x 2, fluorenyl aromatic carbons), 127.0 (x 2, fluorenyl aromatic carbons), 124.9 (x 2, fluorenyl aromatic carbons), 124.4* (C-8), 120.9* (C-9), 119.9 (x 2, fluorenyl aromatic carbons), 109.6* (C-12), 106.1 (x 2, C-2), 61.5* (C-2´), 47.2 (C-5´), 67.9* (C-4´), 31.2*, 28.4*, 24.5* (3 x proline ring CH_2), 60.8 (OMe), 55.8 (2 x OMe), 55.7 (OMe) (the signals marked with * appear low and broad); HR ESMS *m/z* 657.2582 $(M+Na)^+$. Calc. for $C_{38}H_{38}N_2NaO_7$, 657.2578.

(9*H*-Fluoren-9-yl)methyl ((2*S*,3*R*)-3-hydroxy-1-((2-methoxy-5-((*Z*)-3,4,5-trimethoxystyryl)phenyl) amino)-1-oxobutan-2-yl)carbamate (7.7): yield, 44%, off-white solid, m. p. 145-147 ºC; $[\alpha]_D$ −51.5 (*c* 1.0, $CHCl_3$); IR: ν_{max} 3400, 3300 (br, OH, NH), 1701, 1684 (C=O) cm^{-1}; ^{1}H NMR (500 MHz, $CDCl_3$) δ 8.80 (1H, br s, NH), 8.29 (1H, br s; H-12), 7.76 (2H, br d, *J* ~ 7.3 Hz; fluorenyl aromatic protons), 7.60 (2H, br d, *J* ~ 7.3 Hz; fluorenyl aromatic protons), 7.40 (2H, m; fluorenyl aromatic protons), 7.30 (2H, m; fluorenyl aromatic protons), 7.02 (1H, dd, *J* = 8.3, 1.5 Hz; H-8), 6.69 (1H, d, *J* = 8.3 Hz; H-9), 6.53 (2H, s; H-2), 6.50 (1H, d, *J* = 12.2 Hz; H-5 or H-6), 6.45 (1H, d, *J* = 12.2 Hz; H-6 or H-5), 6.00 (1H, br d, *J* ~ 7 Hz, NH), 4.50-4.40 (3H, m; H-2´, H-4´a, threonine β-H), 4.32 (1H, br d, *J* ~ 7 Hz; H-4´b), 4.25 (1H, t, *J* = 7 Hz; H-5´), 3.85 (3H, s; OMe), 3.74 (3H, br s; OMe), 3.69 (6H, s; 2 x OMe), 3.45 (1H, br s, OH), 1.23 (3H, br d, *J* ~ 6.5 Hz; threonine Me); ^{13}C NMR (125 MHz, $CDCl_3$) δ 168.7 (C-1´), 156.9 (C-3´), 152.8 (x 2, C-3), 147.5 (C-10), 143.6 (x 2, fluorenyl aromatic carbons), 141.2 (x 2, fluorenyl aromatic carbons), 137.1 (C-4), 132.7 (C-1), 130.0 (C-7), 126.7 (C-11), 129.5, 129.2 (C-5, C-6, 127.7 (x 2, fluorenyl aromatic carbons), 127.0 (x 2, fluorenyl aromatic carbons), 125.0 (x 2, fluorenyl aromatic carbons), 124.9 (C-8), 121.1 (C-9), 119.9 (x 2, fluorenyl aromatic carbons), 109.7 (C-12), 106.1 (x 2, C-2), 66.7 (threonine β-C), 59.4 (C-2´), 47.1 (C-5´), 67.4 (C-4´), 60.8 (OMe), 55.9 (2 x OMe), 55.8 (OMe), 18.2 (threonine Me); HR ESMS *m/z* 661.2521 $(M+Na)^+$. Calc. for $C_{37}H_{38}N_2NaO_8$, 661.2526.

(9*H*-Fluoren-9-yl)methyl (*S*,*Z*)-(3-(4-hydroxyphenyl)-1-((2-methoxy-5-(3,4,5-trimethoxystyryl)phenyl) amino)-1-oxopropan-2-yl)carbamate (7.8): yield, 60%, off-white solid, m. p. 94-96 °C; $[\alpha]_D$ −25.3 (*c* 1.0, $CHCl_3$); IR: ν_{max} 3400, 3300 (br, OH, NH), 1700 (br, C=O) cm^{-1}; ^1H NMR (500 MHz, $CDCl_3$) δ 8.20 (1H, br s; H-12), 7.85 (1H, br s, NH), 7.77 (2H, br d, *J* ~ 7.3 Hz; fluorenyl aromatic protons), 7.57 (2H, br d, *J* ~ 4 Hz; fluorenyl aromatic protons), 7.40 (2H, br t, *J* ~ 6.5 Hz), 7.30 (2H, m; fluorenyl aromatic protons), 7.01 (3H, m; H-8 and two tyrosine aromatic protons), 6.68 (3H, m; H-9 and two tyrosine aromatic protons), 6.54 (1H, d, *J* = 12.2 Hz; H-5 or H-6), 6.49 (2H, s; H-2), 6.46 (1H, d, *J* = 12.2 Hz; H-6 or H-5), 5.75 (1H, br s, NH), 5.50 (1H, br s, OH), 4.55-4.35 (3H, m; H-2´, H-4´), 4.22 (1H, t, *J* = 6.5 Hz; H-5´), 3.86 (3H, s, OMe), 3.78 (3H, s, OMe), 3.65 (6H, br s, 2 x OMe), 3.14 (1H, m; tyrosine CH_2), 2.98 (1H, m; tyrosine CH_2); ^{13}C NMR (125 MHz, $CDCl_3$) δ 168.9 (C-1´), 156.0* (C-3´), 155.3 (tyrosine aromatic carbon), 152.8 (x 2, C-3), 147.3 (C-10), 143.6 (x 2, fluorenyl aromatic carbons), 141.2 (x 2, fluorenyl aromatic carbons), 136.8 (C-4), 132.8 (C-1), 130.0 (C-7), 126.4 (C-11), 130.3 (x 2, tyrosine aromatic carbons), 129.6, 129.2 (C-5, C-6), 127.7 (x 3, 2 x fluorenyl + 1 x quaternary tyrosine aromatic carbons), 127.0 (x 2, fluorenyl aromatic carbons), 124.9 (x 2, fluorenyl aromatic carbons), 124.7 (C-8), 120.9* (C-9), 119.9 (x 2, fluorenyl aromatic carbons), 115.6 (x 2, tyrosine aromatic carbons), 109.6 (C-12), 106.1 (x 2, C-2), 57.3* (C-2´), 47.1 (C-5´); 67.2* (C-4´), 37.9 (tyrosine CH_2); 60.8 (OMe), 55.8 (2 x OMe), 55.7 (OMe) (the signals marked with * appear low and broad); HR ESMS *m/z* 723.2676 $(M+Na)^+$. Calc. for $C_{42}H_{40}N_2NaO_7$, 723.2682.

(9*H*-Fluoren-9-yl)methyl (*S*,*Z*)-(1-((2-methoxy-5-(3,4,5-trimethoxystyryl)phenyl)amino)-4-(methylthio)-1-oxobutan-2-yl)carbamate (7.9): yield, 69%, off-white solid, m. p. 71-73 °C; $[\alpha]_D$ −23.3 (*c* 1.0, $CHCl_3$); IR: ν_{max} 3400, 3320 (br, NH), 1700, 1686 (br, C=O) cm^{-1}; ^1H NMR (500 MHz, $CDCl_3$) δ 8.40 (1H, br s, NH), 8.32 (1H, d, *J* = 1.5 Hz; H-12), 7.76 (2H, d, *J* = 7.8 Hz; fluorenyl aromatic protons), 7.60 (2H, d, *J* = 7.4 Hz; fluorenyl aromatic protons), 7.38 (2H, br t, *J* ~ 7 Hz; fluorenyl aromatic protons), 7.30 (2H, m; fluorenyl aromatic protons), 7.03 (1H, dd, *J* = 8.3, 1.5 Hz; H-8), 6.70 (1H, d, *J* = 8.3 Hz; H-9), 6.54 (2H, s; H-2), 6.51 (1H, d, *J* = 12.2 Hz; H-5 or H-6), 6.45 (1H, d, *J* = 12.2 Hz; H-6 or H-5), 5.80 (1H, br d, *J* ~ 7 Hz, NH), 4.57 (1H, m; H-2´), 4.44 (2H, d, *J* = 7 Hz; H-4´), 4.23 (1H, t, *J* = 7 Hz; H-5´), 3.85 (3H, s; OMe), 3.78 (3H, br s; OMe), 3.69 (6H, s; 2 x OMe), 2.70-2.55 (2H, br m; CH_2S), 2.20-2.15 (1H, m; methionine β-H), 2.12 (3H, br s; SMe), 2.10-2.05 (1H, m; methionine β-H); ^{13}C NMR (125 MHz, $CDCl_3$) δ 169.0 (C-1´), 156.0* (C-3´), 152.8 (x 2), 147.3 (C-10), 143.7 (x 2, fluorenyl aromatic carbons), 141.2 (x 2, fluorenyl aromatic carbons), 137.2 (C-4), 132.8 (C-1), 130.1 (C-7), 126.7* (C-11), 129.5, 129.2 (C-5, C-6), 127.7 (x 2, fluorenyl aromatic carbons), 127.0 (x 2, fluorenyl aromatic carbons), 124.9 (x 2, fluorenyl aromatic carbons), 124.8 (C-8), 121.0 (C-9)*, 119.9 (x 2, fluorenyl aromatic carbons), 109.6 (C-12), 106.1 (x 2, C-2), 54.6* (C-2´), 47.1 (C-5´), 67.1* (C-4´), 31.6, 30.1 (2 x methionine CH_2), 60.8 (OMe), 55.8 (2 x OMe), 55.7 (OMe), 15.1 (MeS) (the signals marked with * appear low and broad); HR ESMS *m/z* 691.2460 $(M+Na)^+$. Calc. for $C_{38}H_{40}N_2NaO_7S$, 691.2454.

(*Z*)-2-Amino-*N*-(2-methoxy-5-(3,4,5-trimethoxystyryl)phenyl)acetamide (8.1): yield, 59%, colorless oil; IR: ν_{max} 3400, 3300 (br, NH, NH_2), 1683 (br, C=O) cm^{-1}; ^1H NMR (500 MHz, $CDCl_3$) 9.60 (1H, br s, NH), 8.37 (1H, br s; H-12), 7.00 (1H, dd, *J* = 8.3, 1.5 Hz; H-8), 6.73 (1H, d, *J* = 8.3 Hz; H-9), 6.54 (1H, d, *J* = 12.2 Hz; H-5 or H-6), 6.52 (2H, s; H-2), 6.44 (1H, d, *J* = 12.2 Hz; H-6 or H-5), 3.87 (3H, s; OMe), 3.83 (3H, s; OMe), 3.68 (6H, s; 2 x OMe), 3.50 (2H, br s; H-2´), 2.00 (2H, br s, NH_2); ^{13}C NMR

(125 MHz, $CDCl_3$) δ 170.5 (C-1´), 152.8 (x 2; C-3), 147.5 (C-10), 137.1 (C-4), 132.8 (C-1), 130.1 (C-7), 127.2 (C-11), 129.8, 129.0 (C-5, C-6), 124.2 (C-8), 120.6 (C-9), 109.6 (C-12), 106.1 (x 2, C-2), 45.5 (C-2´), 60.8 (OMe), 55.9 (2 x OMe), 55.8 (OMe); HR ESMS *m/z* 373.1769 $(M+H)^+$. Calc. for $C_{20}H_{25}N_2O_5$, 373.1763.

(*S*,*Z*)-2-Amino-*N*-(2-methoxy-5-(3,4,5-trimethoxystyryl)phenyl)propanamide (8.2): yield, 41%, colorless oil; $[\alpha]_D$ −7.6 (*c* 1.0, $CHCl_3$); IR: ν_{max} 3300 (br, NH, NH_2), 1685 (br, C=O) cm^{-1}; ^1H NMR (500 MHz, $CDCl_3$) δ 9.75 (1H, br s, NH), 8.42 (1H, d, *J* = 2 Hz; H-12), 7.00 (1H, dd, *J* = 8.8, 2 Hz; H-8), 6.72 (1H, d, *J* = 8.8 Hz; H-9), 6.54 (1H, d, *J* = 12.2 Hz; H-5 or H-6), 6.53 (2H, s; H-2), 6.43 (1H, d, *J* = 12.2 Hz; H-6 or H-5), 3.87 (3H, s; OMe), 3.84 (3H, s; OMe), 3.69 (6H, s; 2 x OMe), 3.65 (1H, q, *J* = 7 Hz; H-2´), 1.80 (2H, br s, NH_2), 1.43 (3H, d, *J* = 7 Hz; alanine Me); ^{13}C NMR (125 MHz, $CDCl_3$) δ 173.6 (C-1´), 152.8 (x 2, C-3), 147.5 (C-10), 137.1 (C-4), 132.9 (C-1), 130.1 (C-7), 127.3 (C-11), 129.9, 129.0 (C-5, C-6), 124.2 (C-8), 120.5 (C-9), 109.6 (C-12), 106.1 (x 2, C-2), 51.6 (C-2´); 60.8 (OMe), 55.9 (2 x OMe), 55.8 (OMe), 21.7 (alanine Me); HR ESMS *m/z* 387.1925 $(M+H)^+$. Calc. for $C_{21}H_{27}N_2O_5$, 387.1920.

(*S*,*Z*)-2-Amino-*N*-(2-methoxy-5-(3,4,5-trimethoxystyryl)phenyl)-3-methylbutanamide (8.3): yield, 87%, colorless oil; $[\alpha]_D$ −34.4 (*c* 0.7, $CHCl_3$); IR: ν_{max} 3400, 3300 (br, NH, NH_2), 1682 (C=O) cm^{-1}; ^1H NMR (500 MHz, $CDCl_3$) δ 9.75 (1H, br s, NH), 8.42 (1H, d, *J* = 2 Hz; H-12), 6.99 (1H, dd, *J* = 8.3, 2 Hz; H-8), 6.72 (1H, d, *J* = 8.3 Hz; H-9), 6.53 (2H, s; H-2), 6.52 (1H, d, *J* = 12.2 Hz; H-5 or H-6), 6.42 (1H, d, *J* = 12.2 Hz; H-6 or H-5), 3.86 (3H, s; OMe), 3.83 (3H, s; OMe), 3.68 (6H, s; 2 x OMe), 3.36 (1H, d, *J* = 4 Hz; H-2´), 2.39 (1H, m; valine β-H), 1.55 (2H, br s, NH_2), 1.03 (3H, d, *J* = 6.8 Hz; valine Me), 0.87 (3H, d, *J* = 6.8 Hz; valine Me); ^{13}C NMR (125 MHz, $CDCl_3$) δ 172.4 (C-1´), 152.8 (x 2, C-3), 147.5 (C-10), 137.1 (C-4), 132.9 (C-1), 130.1 (C-7), 127.3 (C-11), 129.8, 128.9 (C-5, C-6), 124.1 (C-8), 120.5 (C-9), 109.6 (C-12), 106.1 (x 2, C-2), 60.8 (C-2´), 30.9 (valine β-C), 60.9 (OMe), 55.9 (x 2, OMe), 55.8 (OMe), 19.7, 16.1 (2 x valine Me); HR ESMS *m/z* 415.2235 $(M+H)^+$. Calc. for $C_{23}H_{31}N_2O_5$, 415.2233.

(*S*,*Z*)-2-Amino-*N*-(2-methoxy-5-(3,4,5-trimethoxystyryl)phenyl)-4-methylpentanamide (8.4): yield, 87%, colorless oil; $[\alpha]_D$ −19.7 (*c* 1.0, $CHCl_3$); IR: ν_{max} 3400, 3300 (br, NH, NH_2), 1684 (C=O) cm^{-1}; ^1H NMR (500 MHz, $CDCl_3$) δ 9.80 (1H, br s, NH), 8.41 (1H, d, *J* = 2 Hz; H-12), 6.99 (1H, dd, *J* = 8.3, 2 Hz; H-8), 6.72 (1H, d, *J* = 8.3 Hz; H-9), 6.53 (2H, s; H-2), 6.52 (1H, d, *J* = 12.2 Hz; H-5 or H-6), 6.43 (1H, d, *J* = 12.2 Hz; H-6 or H-5), 3.87 (3H, s; OMe), 3.83 (3H, s; OMe), 3.68 (6H, s; 2 x OMe), 3.51 (1H, br dd, *J* ~ 9.5, 3 Hz; H-2´), 1.85-1.75 (2H, m; leucine CH_2), 1.60 (2H, br s, NH_2), 1.44 (1H, m; isopropyl CH), 0.99 (3H, d, *J* = 6.5 Hz; isopropyl Me), 0.97 (3H, d, *J* = 6.5 Hz; isopropyl Me); ^{13}C NMR (125 MHz, $CDCl_3$) δ 173.5 (C-1´), 152.8 (x 2, C-3), 147.5 (C-10), 137.1 (C-4), 132.9 (C-1), 130.1 (C-7), 127.4 (C-11), 129.9, 128.9 (C-5, C-6), 124.0 (C-8), 120.5 (C-9), 109.5 (C-12), 106.1 (x 2, C-2), 54.4 (C-2´), 25.0 (isopropyl CH), 44.1 (leucine CH_2), 60.8 (OMe), 55.9 (2 x OMe), 55.8 (OMe), 23.3, 21.4 (2 x isopropyl Me); HR ESMS *m/z* 429.2390 $(M+H)^+$. Calc. for $C_{24}H_{33}N_2O_5$, 429.2389.

(*S*,*Z*)-2-Amino-*N*-(2-methoxy-5-(3,4,5-trimethoxystyryl)phenyl)-3-phenylpropanamide (8.5): yield, 72%, off-white solid m. p. 40-42 ºC; $[\alpha]_D$ −60.0 (*c* 0.9, $CHCl_3$); IR: ν_{max} 3300 (br, NH, NH_2), 1683 (C=O) cm^{-1}; ^1H NMR (500 MHz, $CDCl_3$) δ 9.80 (1H, br s, NH), 8.45 (1H, d, *J* = 2 Hz; H-12), 7.35-7.25 (5H, br m; phenylalanine aromatic protons), 7.01 (1H, dd, *J* = 8.3, 1.5 Hz; H-8), 6.72 (1H, d, *J* = 8.3 Hz;

H-9), 6.55 (1H, d, J = 12.2 Hz; H-5 or H-6), 6.54 (2H, s; H-2), 6.45 (1H, d, J = 12.2 Hz; H-6 or H-5), 3.85 (6H, s; 2 x OMe), 3.72 (1H, m; H-2´), 3.70 (6H, s; 2 x OMe), 3.38 (1H, dd, J = 13.5, 3.5 Hz; phenylalanine β-H), 2.78 (1H, dd, J = 13.5, 9.8 Hz; phenylalanine β-H), 1.55 (2H, br s, NH_2); ^{13}C NMR (125 MHz, $CDCl_3$) δ 172.2 (C-1´), 152.8 (x 2, C-2), 147.6 (C-10), 137.9 (phenylalanine aromatic carbon), 137.1 (C-4), 132.9 (C-1), 130.1 (C-7), 126.8 (C-11), 129.8 (C-5 or C-6), 129.2 (x 3, phenylalanine aromatic carbons), 129.0 (C-6 or C-5), 128.7 (x 2, phenylalanine aromatic carbons), 124.2 (C-8), 120.5 (C-9), 109.5 (C-12), 106.1 (x 2, C-2), 57.3 (C-2´), 40.8 (phenylalanine CH_2), 60.8 (OMe), 55.9 (2 x OMe), 55.8 (OMe); HR ESMS *m/z* 463.2229 $(M+H)^+$. Calc. for $C_{27}H_{31}N_2O_5$, 463.2233.

(*S*,*Z*)-*N*-(2-Methoxy-5-(3,4,5-trimethoxystyryl)phenyl)pyrrolidine-2-carboxamide (8.6): yield, 54%, colorless oil; $[\alpha]_D$ −34.1 (*c* 1.0, $CHCl_3$); IR: ν_{max} 3300 (br, NH), 1684 (C=O) cm^{-1}; ^{1}H NMR (500 MHz, $CDCl_3$) δ 10.0 (1H, br s, NH), 8.39 (1H, d, J = 2 Hz; H-12), 7.00 (1H, dd, J = 8.3, 2 Hz; H-8), 6.71 (1H, d, J = 8.3 Hz; H-9), 6.53 (2H, s; H-2), 6.52 (1H, d, J = 12.2 Hz; H-5 or H-6), 6.43 (1H, d, J = 12.2 Hz; H-6 or H-5), 3.88 (1H, m; H-2´), 3.86 (3H, s; OMe), 3.84 (3H, s; OMe), 3.69 (6H, s; 2 x OMe), 3.10, 3.02 (2 x 1H, m; CH_2N), 2.20 (2H, m; NH + proline ring CH), 2.05 (1H, m; proline ring CH), 1.80-1.75 (2H, m; 2 proline ring CH); ^{13}C NMR (125 MHz, $CDCl_3$) δ 173.1 (C-1´), 152.8 (x 2, C-3), 147.6 (C-10), 137.1 (C-4), 132.8 (C-1), 130.1 (C-7), 127.3 (C-11), 129.8, 128.9 (C-5, C-6), 124.1 (C-8), 120.5 (C-9), 109.6 (C-12), 106.1 (x 2, C-2), 61.4 (C-2´), 47.3, 30.8, 26.2 (proline ring CH_2), 60.8 (OMe), 55.9 (2 x OMe), 55.8 (OMe); HR ESMS *m/z* 413.2070 $(M+H)^+$. Calc. for $C_{23}H_{29}N_2O_5$, 413.2076.

(2*S*,3*R*)-2-Amino-3-hydroxy-*N*-(2-methoxy-5-((*Z*)-3,4,5-trimethoxystyryl)phenyl)butanamide (8.7): yield, 51%, colorless oil; $[\alpha]_D$ −8.2 (*c* 0.7, $CHCl_3$); IR: ν_{max} 3450, 3300 (br, OH, NH, NH_2), 1683 (C=O) cm^{-1}, NMR MN ^{1}H NMR (500 MHz, $CDCl_3$) δ 9.85 (1H, br s, NH), 8.37 (1H, d, J = 2 Hz; H-12), 7.01 (1H, dd, J = 8.3, 2 Hz; H-8), 6.72 (1H, d, J = 8.3 Hz; H-9), 6.52 (2H, s; H-2), 6.51 (1H, d, J = 12.2 Hz; H-5 or H-6), 6.43 (1H, d, J = 12.2 Hz; H-6 or H-5), 4.40 (1H, qd, J = 6.5, 2.5 Hz; threonine β-H), 3.86 (3H, s; OMe), 3.84 (3H, s; OMe), 3.69 (6H, s; 2 x OMe), 3.35 (1H, br d, J ~ 2.5 Hz; H-2´), 2.10 (3H, br s, OH, NH_2), 1.25 (3H, d, J = 6.5 Hz; threonine Me); ^{13}C NMR (125 MHz, $CDCl_3$) δ 171.7 (C-1´), 152.8 (x 2, C-3), 147.6 (C-10), 137.1 (C-4), 132.8 (C-1), 130.1 (C-7), 127.0 (C-11), 129.7, 129.0 (C-5, C-6), 124.5 (C-8), 120.6 (C-9), 109.6 (C-12), 106.1 (x 2, C-2), 67.8 (threonine β-C), 60.4 (C-2´), 60.9 (OMe), 55.9 (2 x OMe), 55.8 (OMe), 19.0 (threonine Me); HR ESMS *m/z* 417.2024 $(M+H)^+$. Calc. for $C_{22}H_{29}N_2O_6$, 417.2026.

(*S*,*Z*)-2-Amino-3-(4-hydroxyphenyl)-*N*-(2-methoxy-5-(3,4,5-trimethoxystyryl)phenyl)propanamide (8.8): yield, 75%, off-white solid 63-65 ºC; $[\alpha]_D$ −61.1 (*c* 0.9, $CHCl_3$); IR: ν_{max} 3400, 3300 (br, OH, NH, NH_2), 1682 (C=O) cm^{-1}; ^{1}H NMR (500 MHz, $CDCl_3$) δ 9.70 (1H, br s, NH), 8.37 (1H, d, J = 2 Hz; H-12), 7.07 (2H, d, J = 8.2 Hz; tyrosine aromatic protons), 7.01 (1H, dd, J = 8.3, 2 Hz; H-8), 6.75 (2H, d, J = 8; tyrosine aromatic protons), 6.72 (1H, d, J = 8.3 Hz; H-9), 6.56 (1H, d, J = 12.2 Hz; H-5 or H-6), 6.52 (2H, s; H-2), 6.45 (1H, d, J = 12.2 Hz; H-5 or H-6), 3.85 (3H, s; OMe), 3.84 (3H, s; OMe), 3.68 (1H, m; H-2´), 3.66 (6H, s; 2 x OMe), 3.17 (1H, dd, J = 13.5, 3.5 Hz; tyrosine β-H), 2.82 (1H, dd, J = 13.5, 8.8 Hz; tyrosine β-H) (OH/NH_2 signals are not observed); ^{13}C NMR (125 MHz, $CDCl_3$) δ 172.7 (C-1´), 155.3 (tyrosine aromatic carbon), 152.8 (x 2, C-3), 147.7 (C-10), 136.9 (C-4), 132.9 (C-1), 130.1 (C-7), 128.6 (tyrosine aromatic carbon), 126.9 (C-11), 130.3 (x 2, tyrosine aromatic carbons), 129.8, 129.1 (C-5, C-6), 124.4 (C-8), 120.7 (C-9), 115.7 (x 2, tyrosine aromatic carbons), 109.7 (C-12), 106.1

(x 2, C-2), 57.3 (C-2´), 40.0 (tyrosine CH_2), 60.9 (OMe), 55.9 (2 x OMe), 55.8 (OMe); HR ESMS *m/z* 479.2187 $(M+H)^+$. Calc. for $C_{27}H_{31}N_2O_6$, 479.2182.

(*S*,*Z*)-2-Amino-*N*-(2-methoxy-5-(3,4,5-trimethoxystyryl)phenyl)-4-(methylthio)butanamide (8.9): yield, 79%, colorless oil; $[\alpha]_D$ −15.5 (*c* 0.7, $CHCl_3$); IR: ν_{max} 3300 (br, NH, NH_2), 1683 (C=O) cm^{-1}; ^1H NMR (500 MHz, $CDCl_3$) δ 9.75 (1H, br s, NH), 8.38 (1H, d, *J* = 2 Hz; H-12), 7.00 (1H, dd, *J* = 8.3, 2 Hz; H-8), 6.71 (1H, d, *J* = 8.3 Hz; H-9), 6.52 (1H, d, *J* = 12.2 Hz; H-5 or H-6), 6.51 (2H, s; H-2), 6.42 (1H, d, *J* = 12.2 Hz; H-6 or H-5), 3.85 (3H, s; OMe), 3.83 (3H, s; OMe), 3.66 (6H, s; 2 x OMe), 3.64 (1H, dd, *J* = 8.3, 4.5 Hz; H-2´), 2.65 (2H, m; CH_2S), 2.26 (1H, m; methionine β-H), 2.11 (3H, s; SMe), 1.84 (1H, m; methionine β-H), 1.65 (2H, br s, NH_2); ^{13}C NMR (125 MHz, $CDCl_3$) δ 172.5 (C-1´), 152.8 (x 2, C-3), 147.5 (C-10), 137.0 (C-4), 132.8 (C-1), 130.1 (C-7), 127.2 (C-11), 129.7, 129.0 (C-5, C-6), 124.2 (C-8), 120.5 (C-9), 109.5 (C-12), 106.1 (x 2, C-2), 55.0 (C-2´), 34.0, 30.6 (2 x methionine CH_2), 60.8 (OMe), 55.9 (2 x OMe), 55.8 (OMe), 15.2 (methionine Me); HR ESMS *m/z* 447.1954 $(M+H)^+$. Calc. for $C_{23}H_{31}N_2O_5S$, 447.1954.

(9*H*-fluoren-9-yl)methyl (*R*,Z)-(1-((2-methoxy-5-(3,4,5-trimethoxystyryl)phenyl)amino)-3-methyl-1-oxobutan-2-yl)carbamate (9.1): yield, 55%, off-white solid, m. p. 176-178 ºC; $[\alpha]_D$ +36 (*c* 1.0, $CHCl_3$); IR, ^1H NMR, ^{13}C NMR and HR ESMS data identical to compound **7.3**.

(9*H*-fluoren-9-yl)methyl (*R*,*Z*)-(1-((2-methoxy-5-(3,4,5-trimethoxystyryl)phenyl)amino)-4-methyl-1-oxopentan-2-yl)carbamate (9.2): yield, 60%, off-white solid, m. p. 72-74 ºC; $[\alpha]_D$ +34.0 (*c* 0.9, $CHCl_3$); IR, ^1H NMR, ^{13}C NMR and HR ESMS data identical to compound **7.4**.

(9*H*-fluoren-9-yl)methyl (*R*,*Z*)-(1-((2-methoxy-5-(3,4,5-trimethoxystyryl)phenyl)amino)-1-oxo-3-phenylpropan-2-yl)carbamate (9.3): yield, 69%, off-white solid, m. p. 76-78 ºC; $[\alpha]_D$ +24.0 (*c* 1.0, $CHCl_3$); IR, ^1H NMR, ^{13}C NMR and HR ESMS data identical to compound **7.5**.

(9*H*-fluoren-9-yl)methyl (*R*,*Z*)-2-((2-methoxy-5-(3,4,5-trimethoxystyryl)phenyl)-carbamoyl) pyrrolidine-1-carboxylate (9.4): yield, 73%, off-white solid, m. p. 69-71 ºC; $[\alpha]_D$ +73.2 (*c* 0.95, $CHCl_3$), IR, ^1H NMR, ^{13}C NMR and HR ESMS data identical to compound **7.6**.

(*R*,*Z*)-2-Amino-*N*-(2-methoxy-5-(3,4,5-trimethoxystyryl)phenyl)-3-methylbutanamide (10.1): yield, 71%, colorless oil; $[\alpha]_D$ +30.8 (*c* 0.9, $CHCl_3$), IR, ^1H NMR, ^{13}C NMR and HR ESMS data identical to compound **8.3**.

(*R*,*Z*)-2-Amino-*N*-(2-methoxy-5-(3,4,5-trimethoxystyryl)phenyl)-4-methylpentanamide (10.2): yield, 67%, colorless oil; $[\alpha]_D$ +19.8 (*c* 0.9, $CHCl_3$); IR, ^1H NMR, ^{13}C NMR and HR ESMS data identical to compound **8.4**.

(*R*,*Z*)-2-Amino-*N*-(2-methoxy-5-(3,4,5-trimethoxystyryl)phenyl)-3-phenylpropanamide (10.3): yield, 84%, off-white solid, m. p. 40-42 ºC; $[\alpha]_D$ +72.1 (*c* 1.0, $CHCl_3$); IR, ^1H NMR, ^{13}C NMR and HR ESMS data identical to compound **8.5**.

(*R*,*Z*)-*N*-(2-Methoxy-5-(3,4,5-trimethoxystyryl)phenyl)pyrrolidine-2-carboxamide (10.4): yield, 68%. colorless oil; $[\alpha]_D$ +32 (*c* 1.0, $CHCl_3$); IR, ^1H NMR, ^{13}C NMR and HR ESMS data identical to compound **8.6**.

Graphical NMR spectra

1

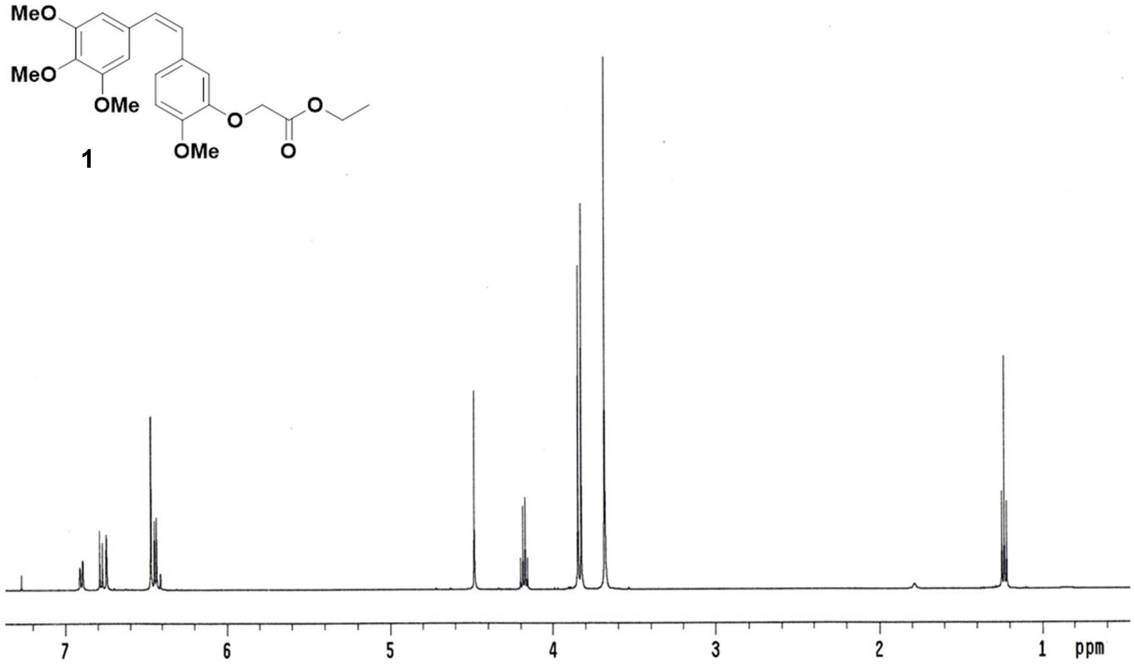

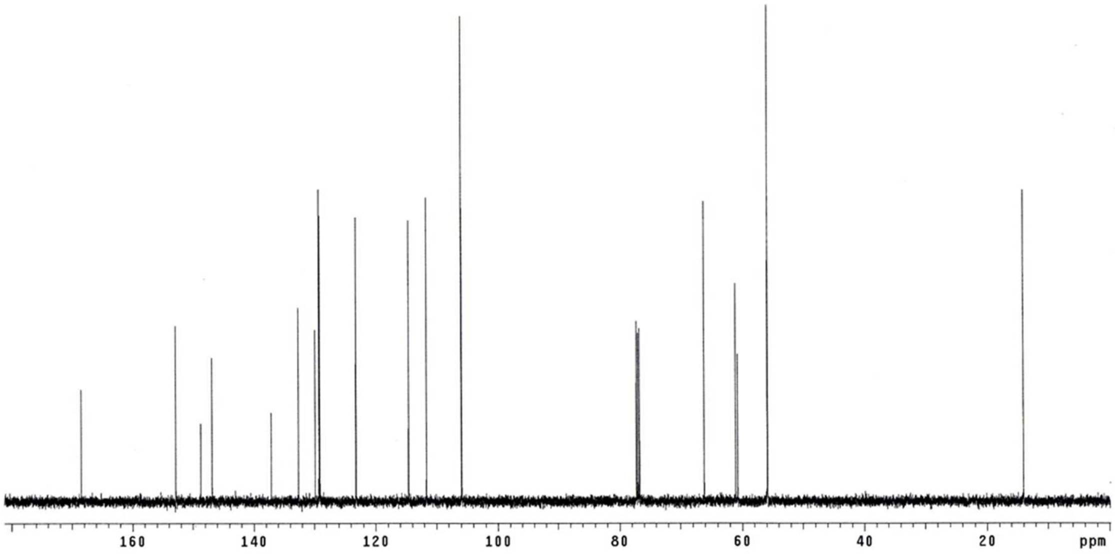

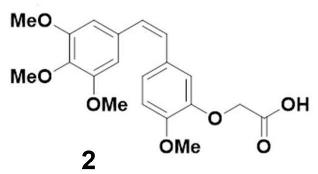
MeO
MeO
OMe
OMe
O
OH
O

2

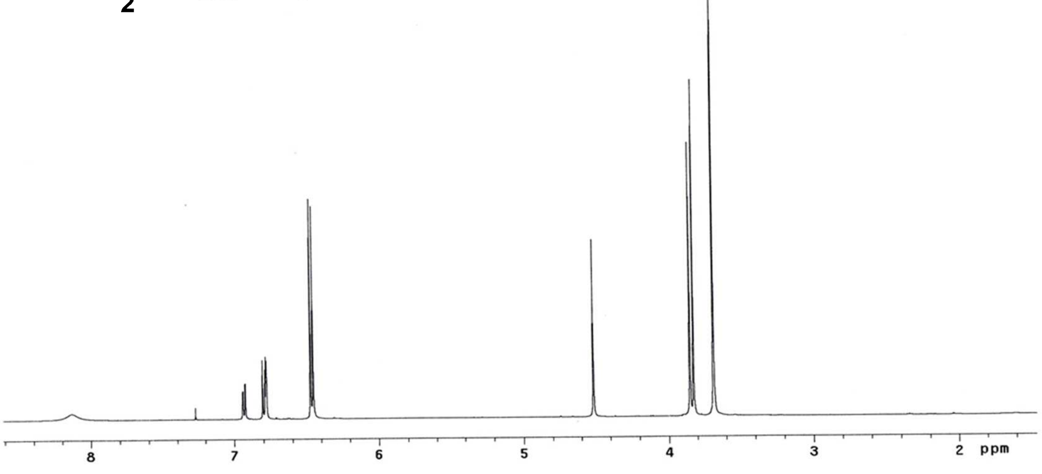
8
7
6
5
4
3
2 ppm

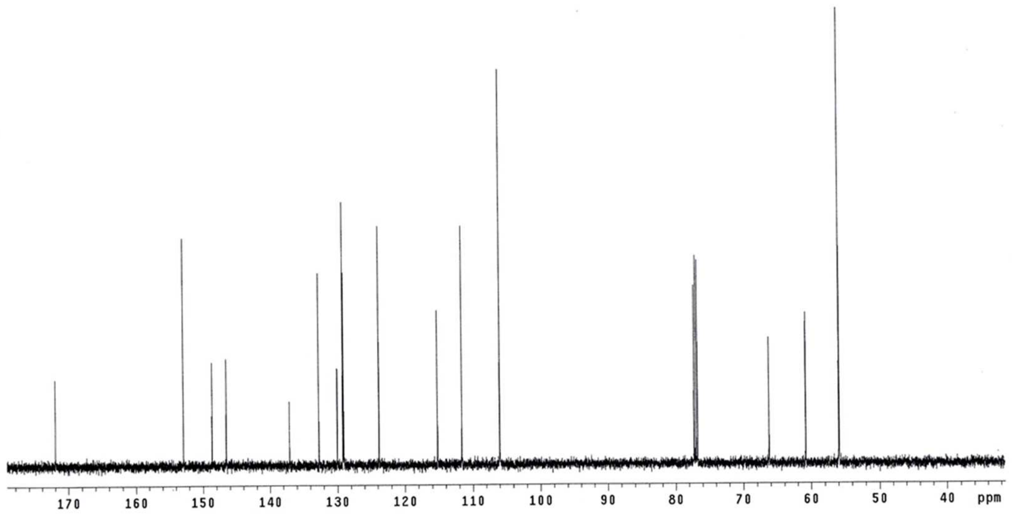
170
160
150
140
130
120
110
100
90
80
70
60
50
40
ppm

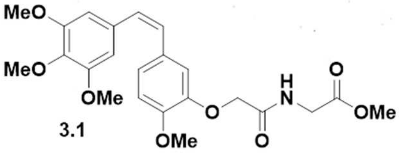
MeO
MeO
OMe
OMe
O
H
N
O
O
OMe

3.1

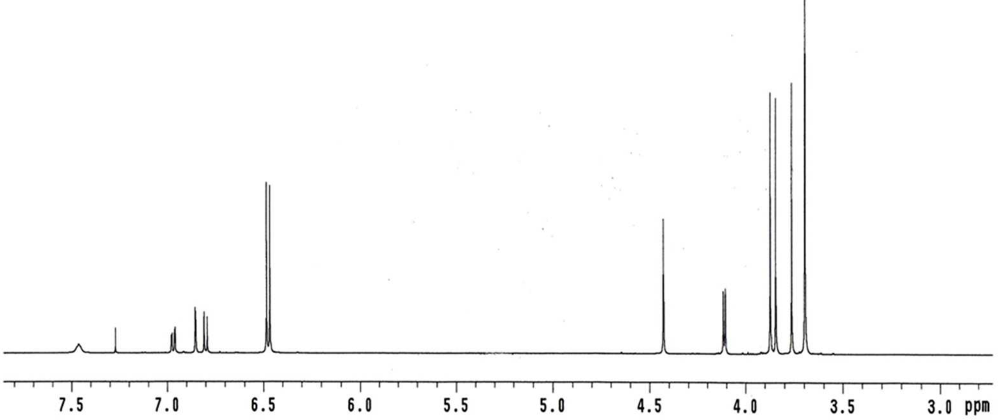
7.5 7.0 6.5 6.0 5.5 5.0 4.5 4.0 3.5 3.0 ppm

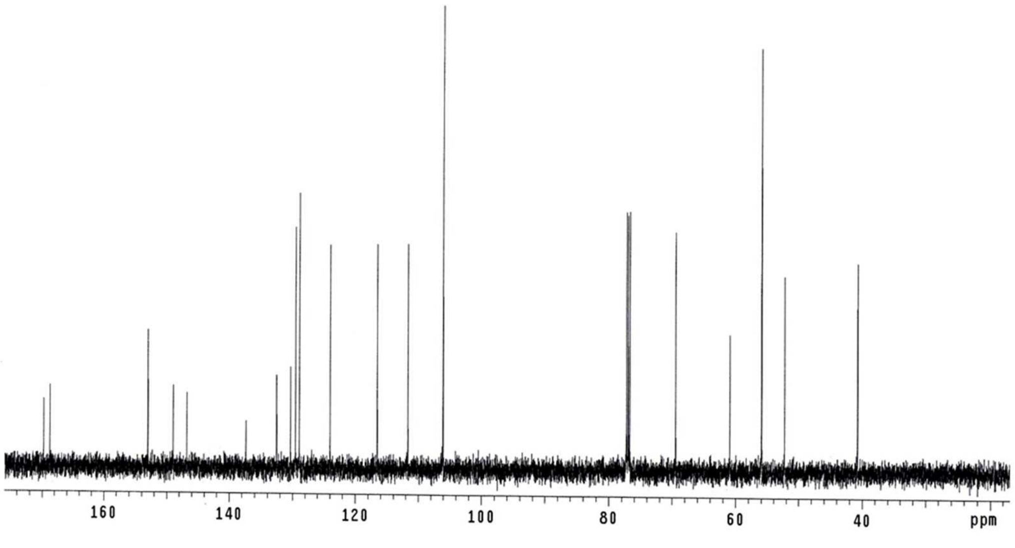
160 140 120 100 80 60 40 ppm

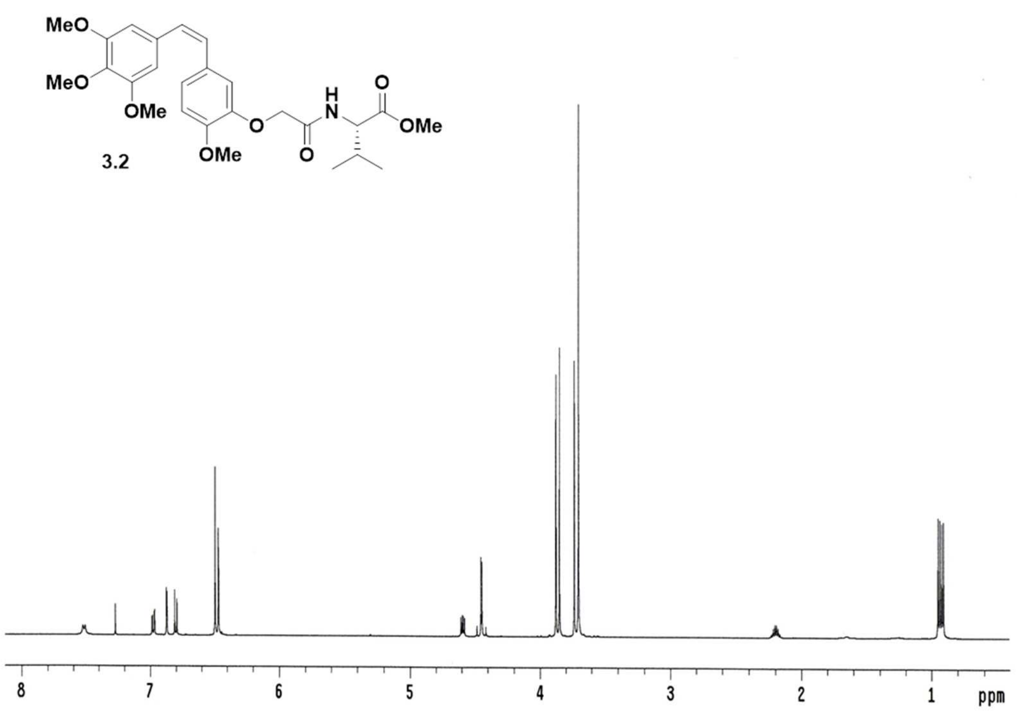
MeO
MeO
OMe
OMe
O
H
N
O
OMe
O
3.2
8
7
6
5
4
3
2
1
ppm

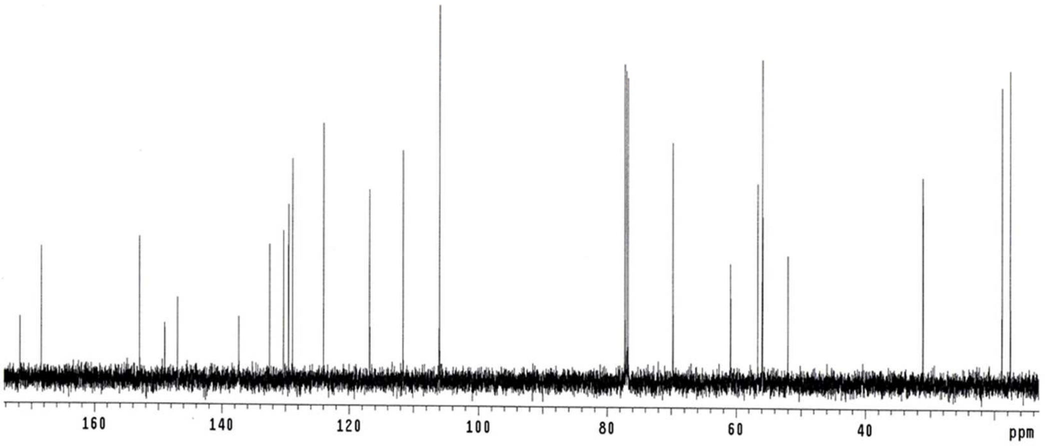
160
140
120
100
80
60
40
ppm

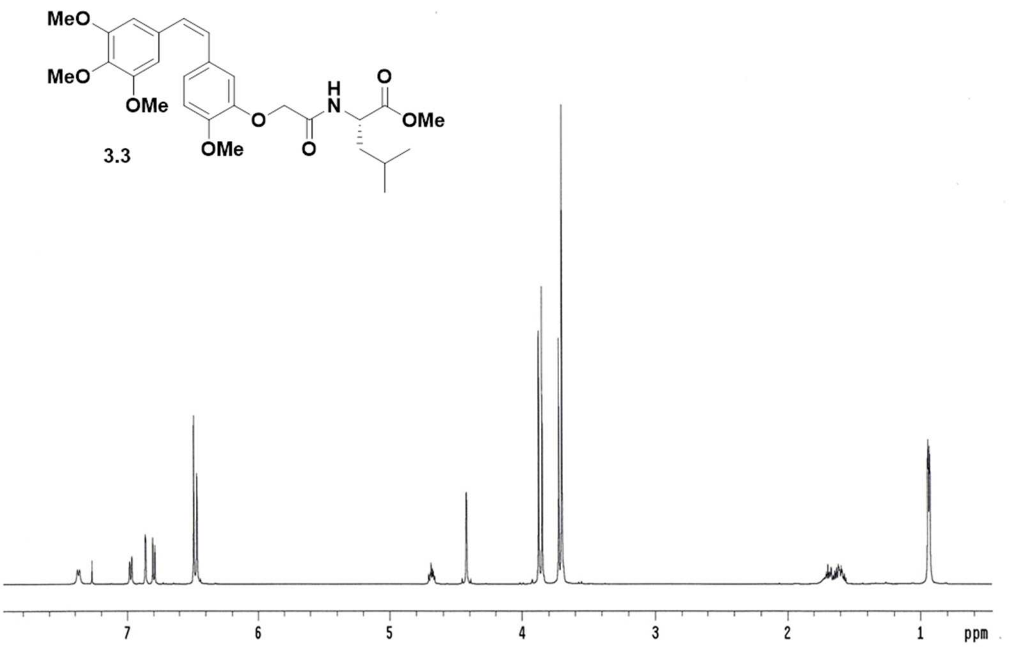
MeO
MeO
OMe
OMe
O
H
N
O
O
OMe
7
6
5
4
3
2
1
ppm

3.3

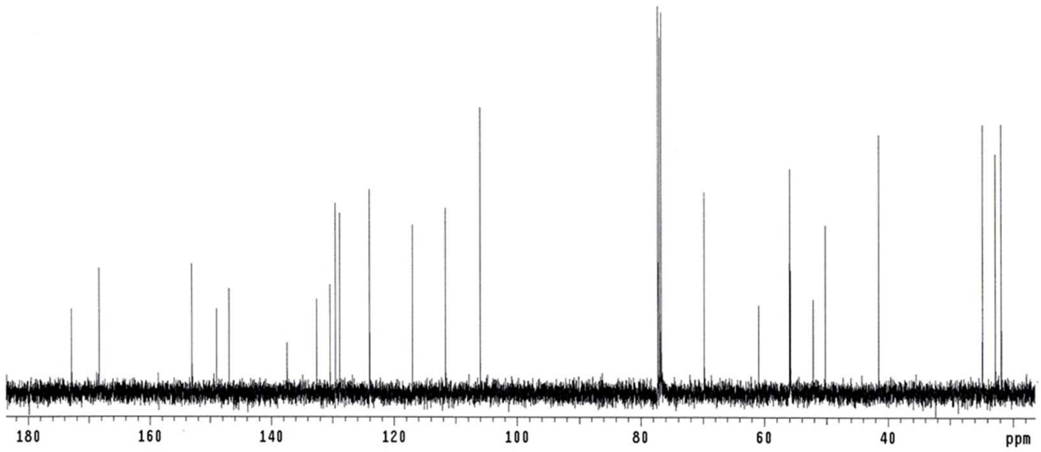
180
160
140
120
100
80
60
40
ppm

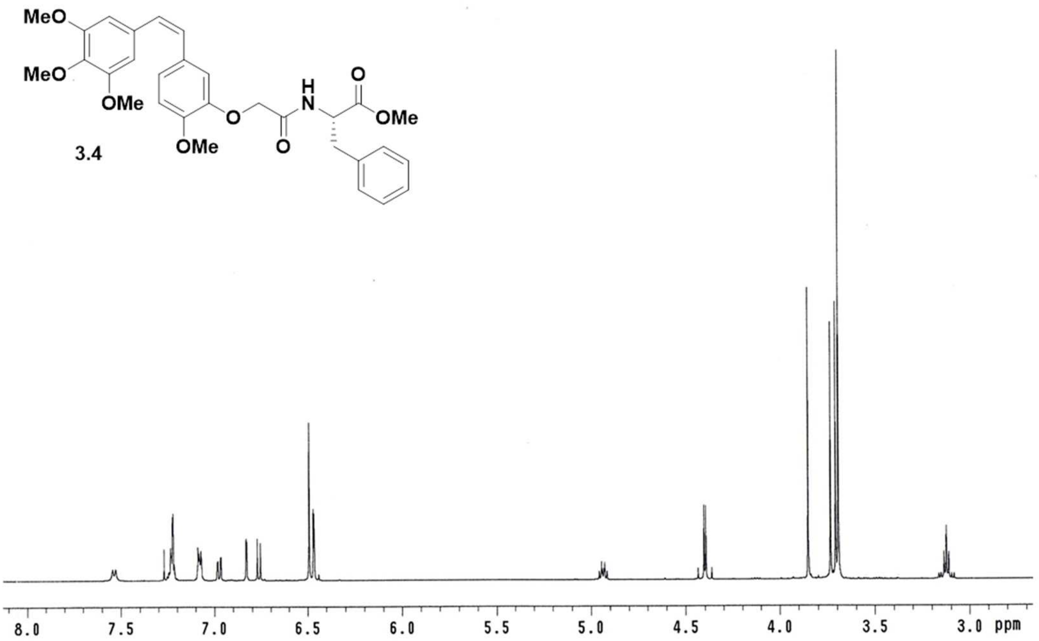
MeO
MeO
OMe
OMe
O
H
N
O
OMe
O
3.4
8.0 7.5 7.0 6.5 6.0 5.5 5.0 4.5 4.0 3.5 3.0 ppm

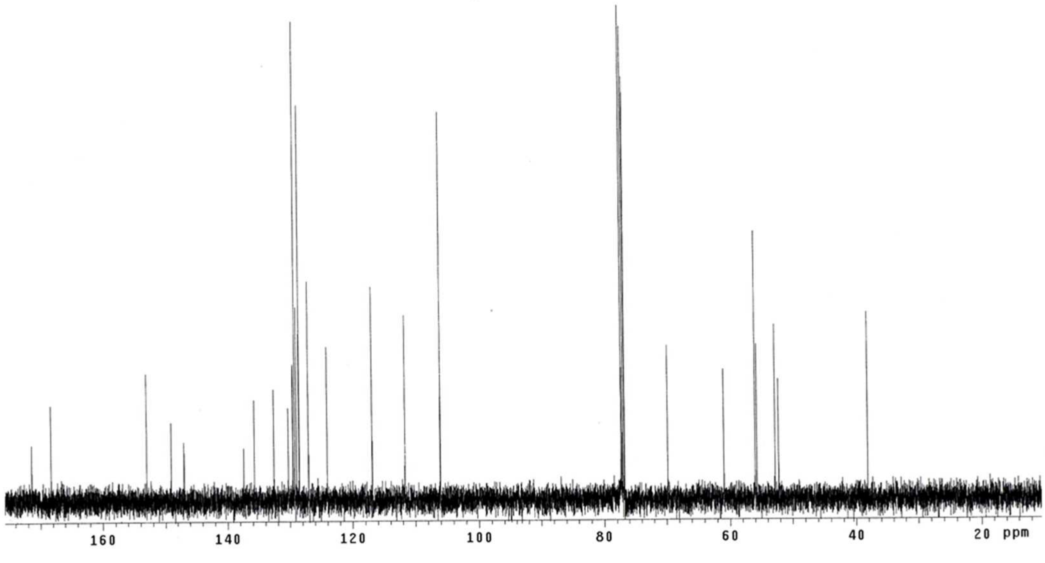
160 140 120 100 80 60 40 20 ppm

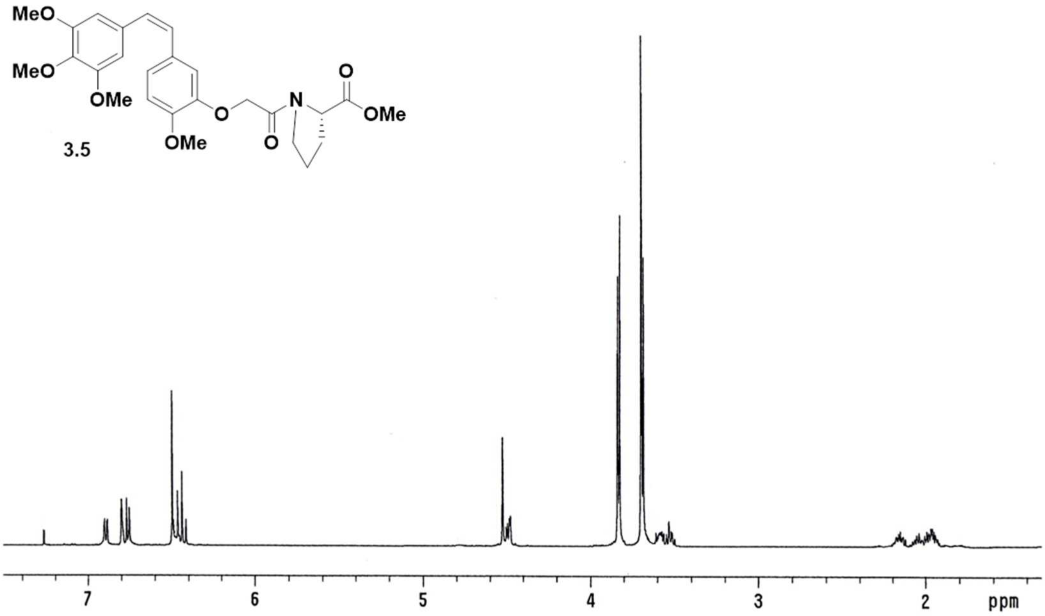
MeO
MeO
OMe
OMe
O
N
O
O
OMe
3.5
7
6
5
4
3
2
ppm

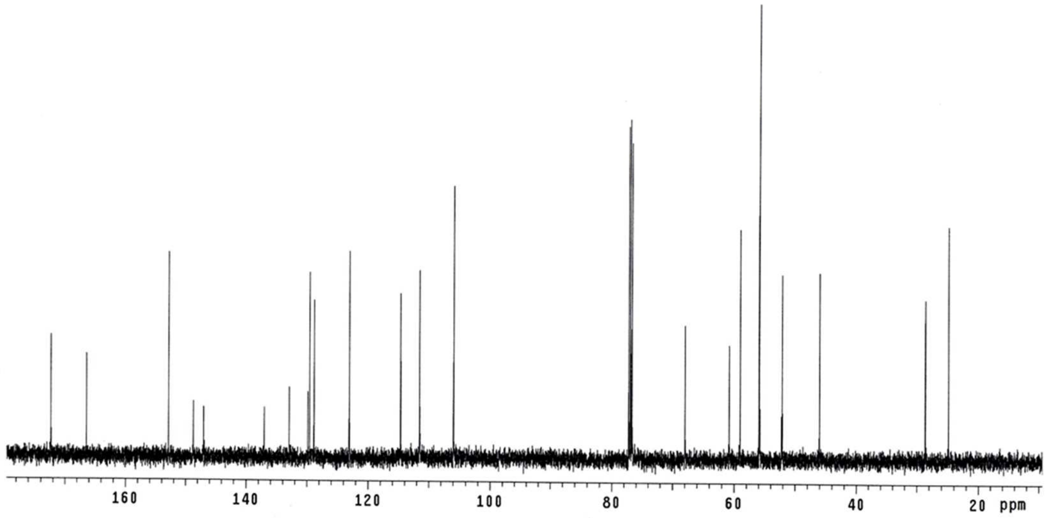
160
140
120
100
80
60
40
20
ppm

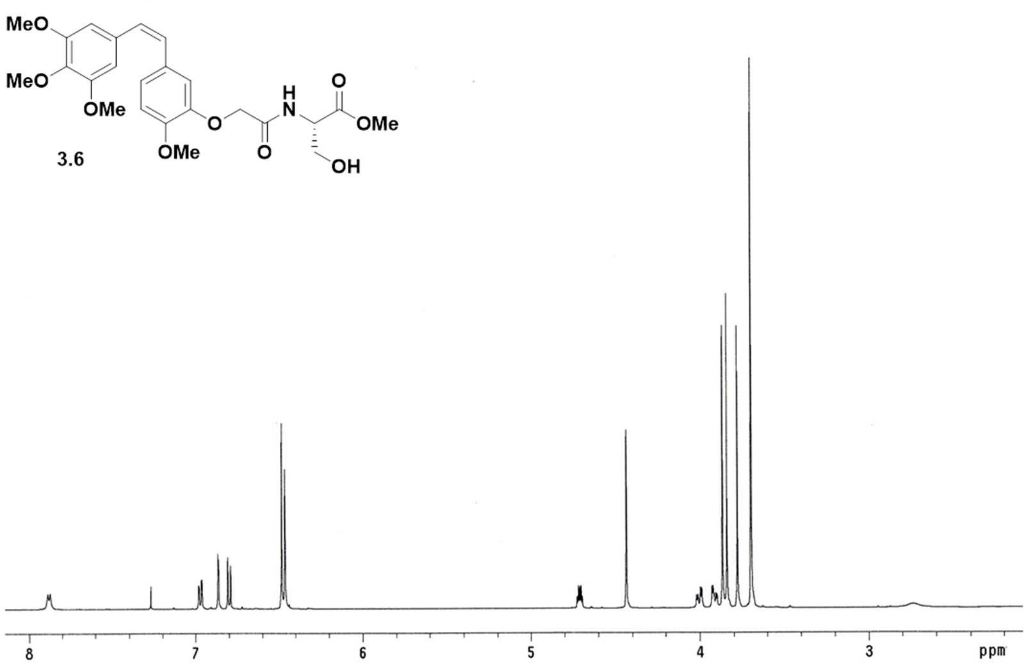
MeO
MeO
OMe
OMe
H
N
O
O
O
OMe
OH
3.6
8
7
6
5
4
3
ppm

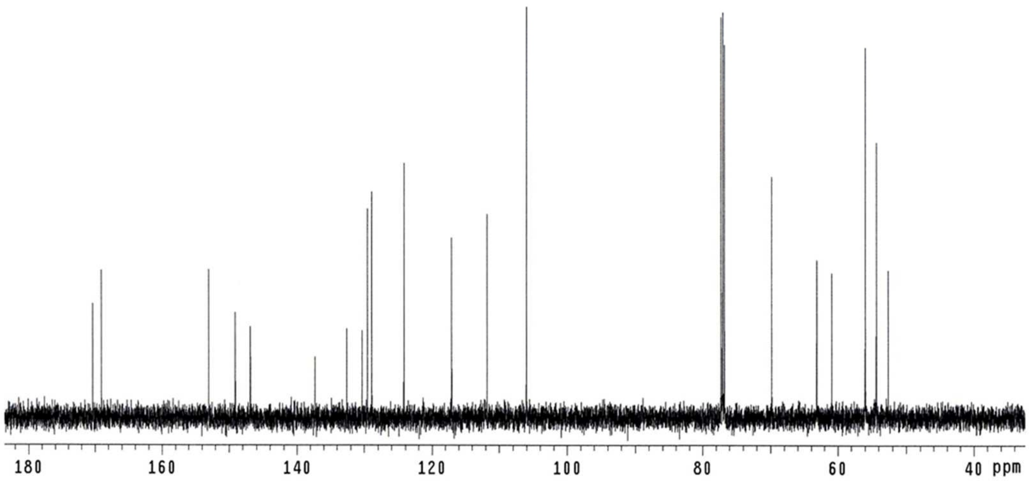
180
160
140
120
100
80
60
40 ppm

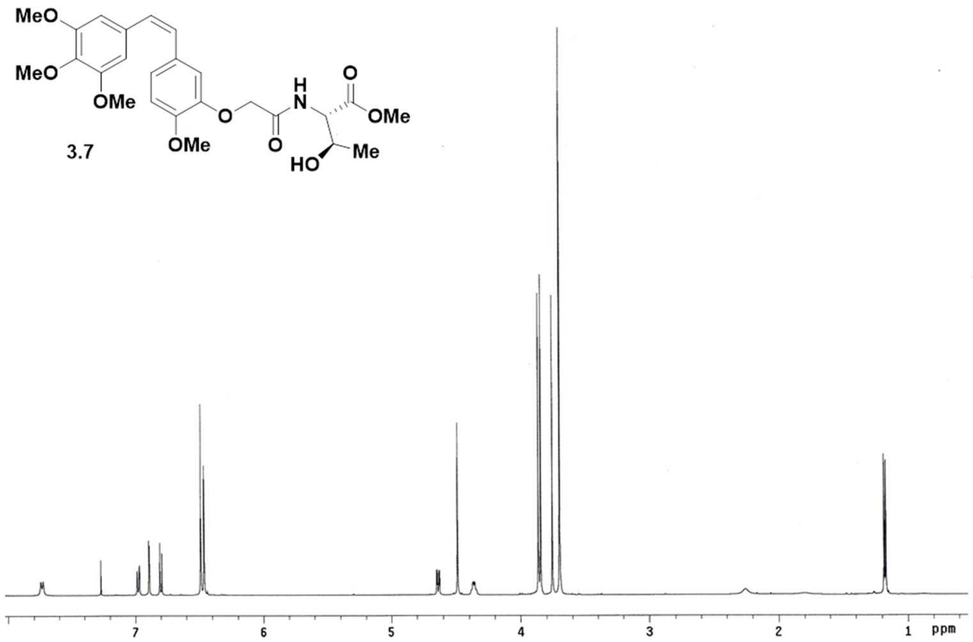
MeO
MeO
OMe
OMe
O
H
N
O
OMe
O
HO
Me
7
6
5
4
3
2
1 ppm

3.7

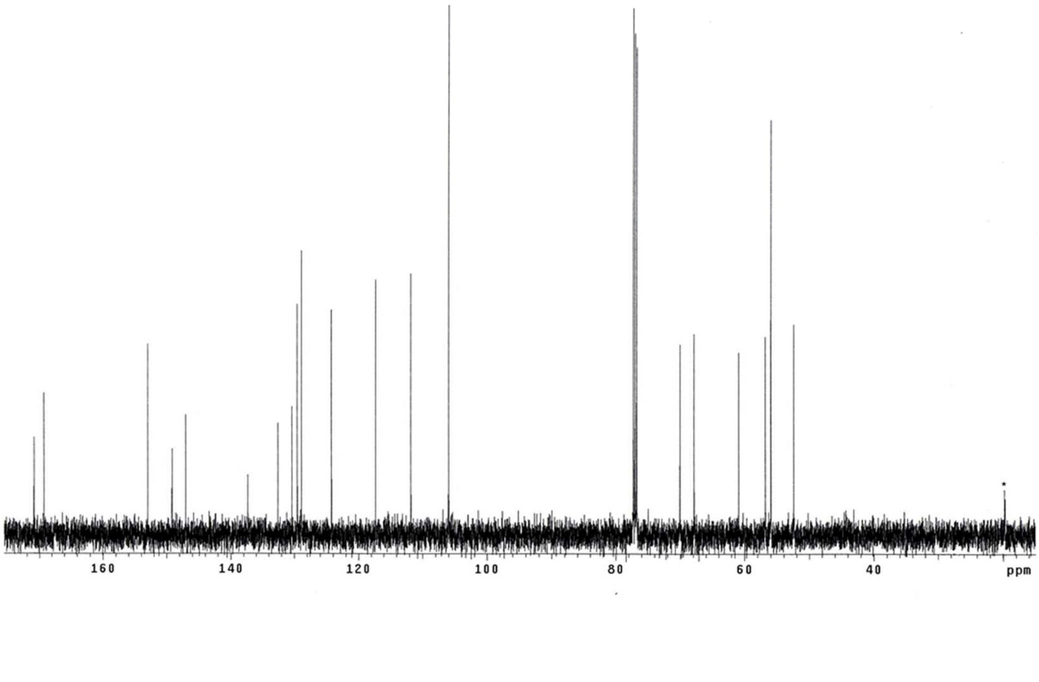
160
140
120
100
80
60
40
ppm

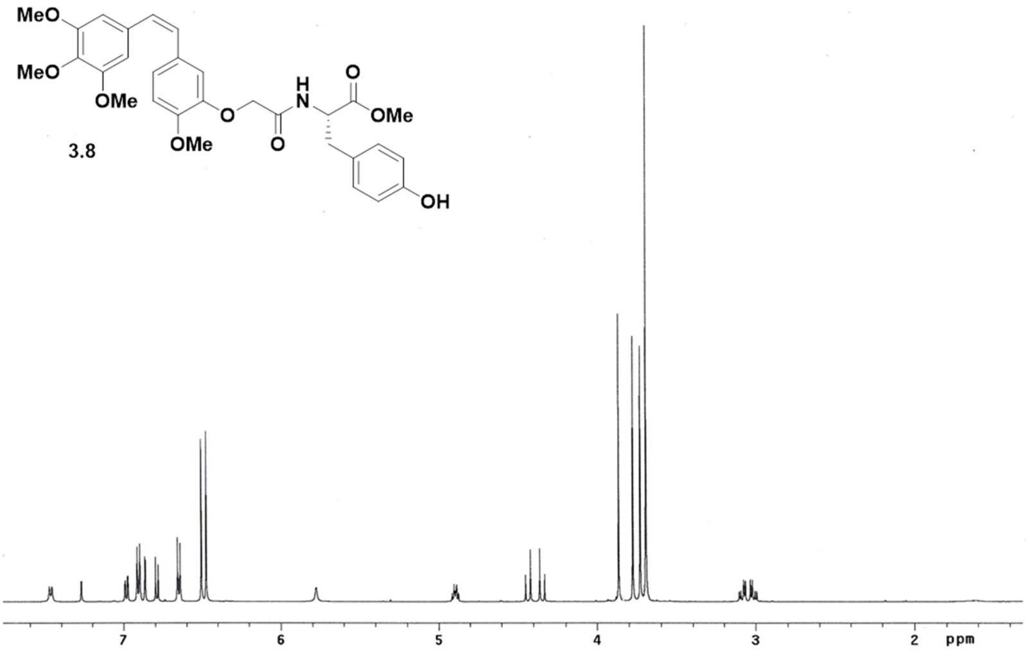
MeO
MeO
OMe
OMe
H
N
O
O
O
OMe
OH
3.8
7
6
5
4
3
2 ppm

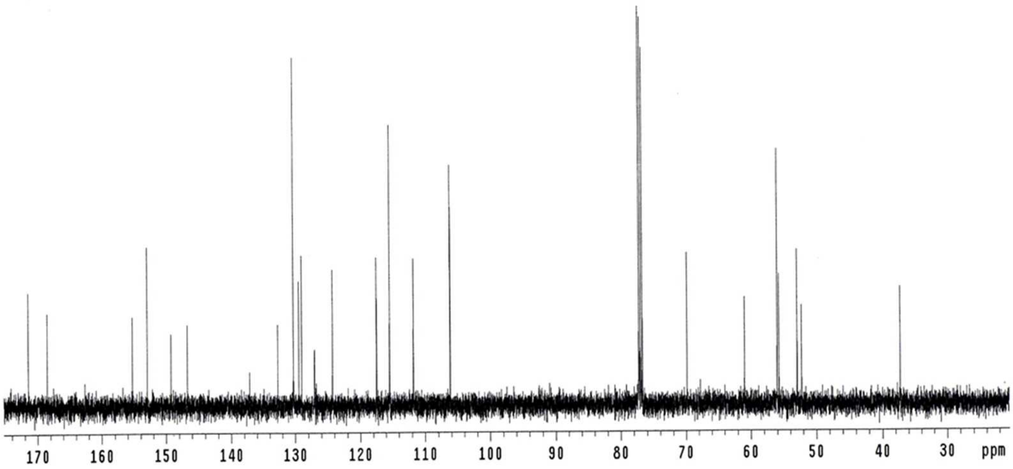
170 160 150 140 130 120 110 100 90 80 70 60 50 40 30 ppm

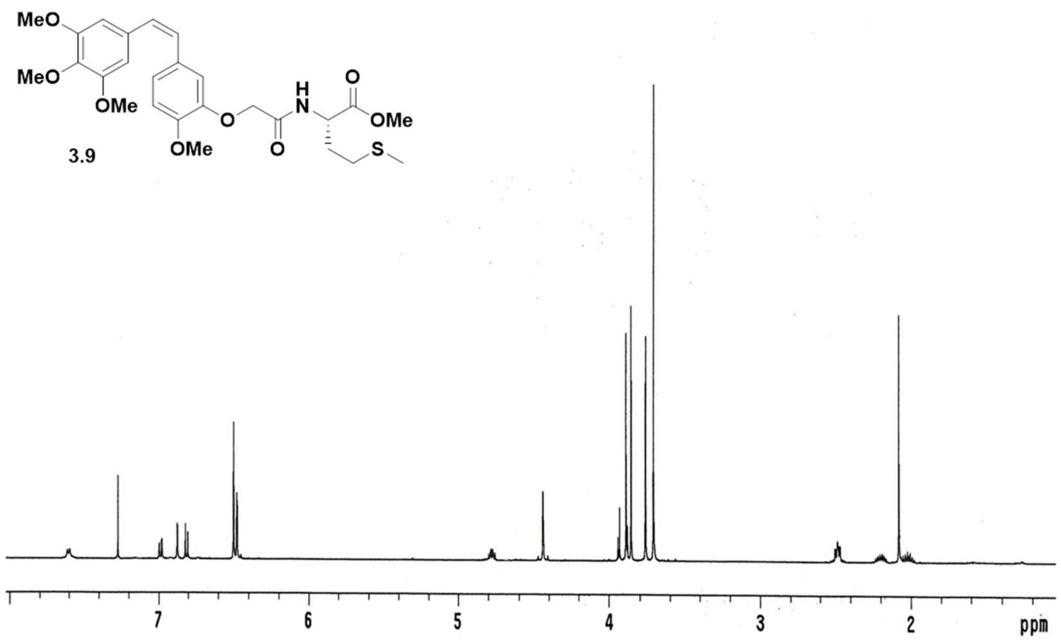
MeO
MeO
OMe
OMe
O
H
N
O
OMe
O
S
3.9
7
6
5
4
3
2
ppm

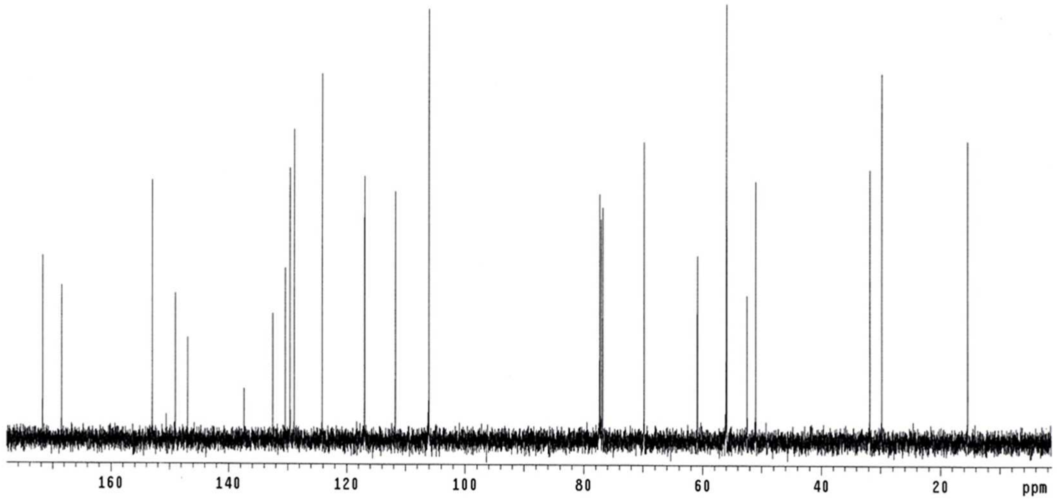
160
140
120
100
80
60
40
20
ppm

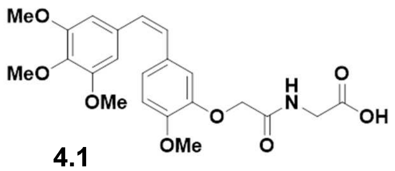
MeO
MeO
OMe
OMe
H
N
O
O
O
OH

4.1

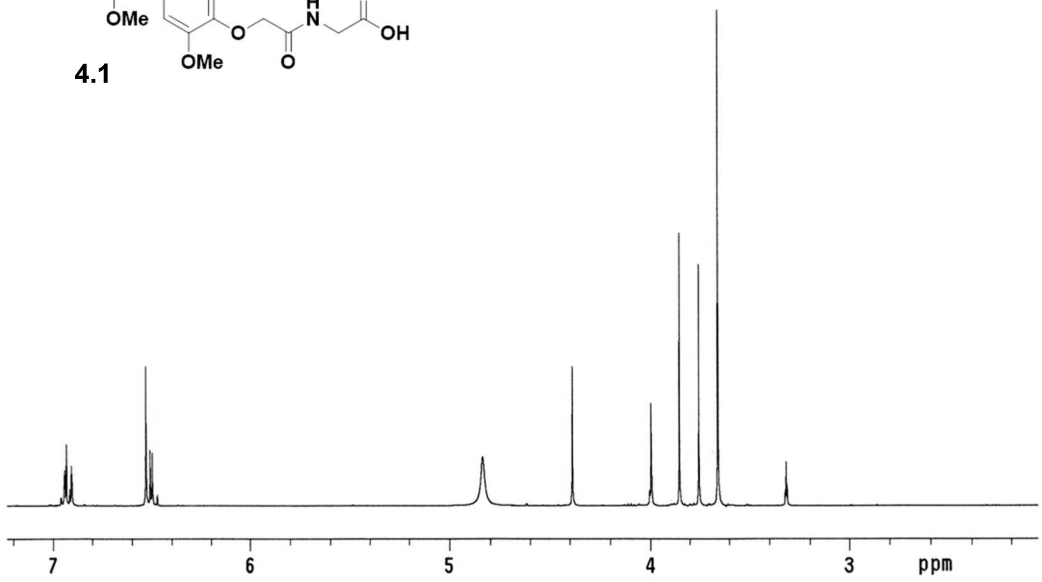
7
6
5
4
3
ppm

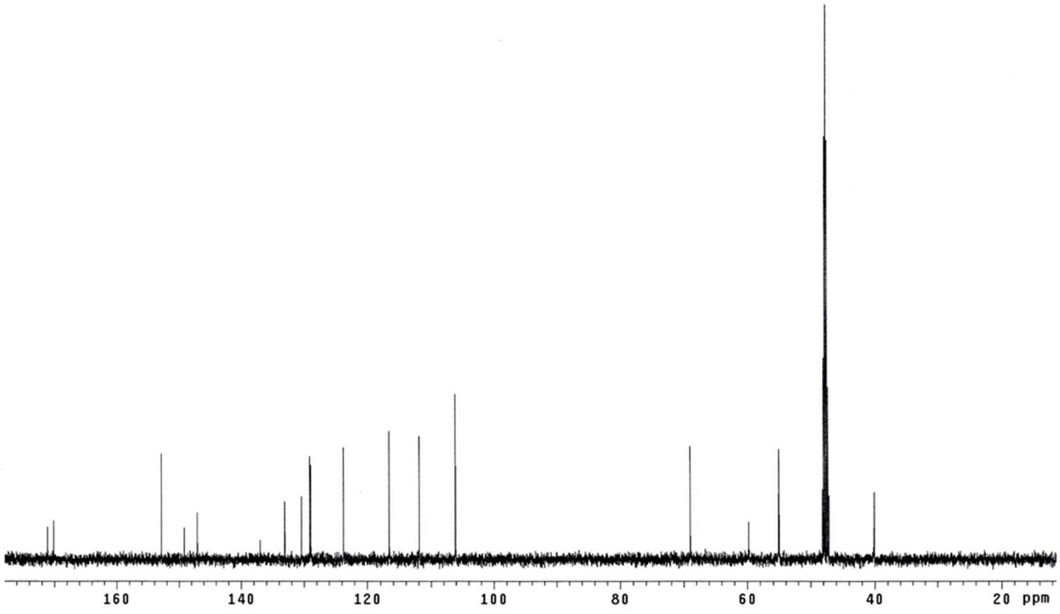
160
140
120
100
80
60
40
20 ppm

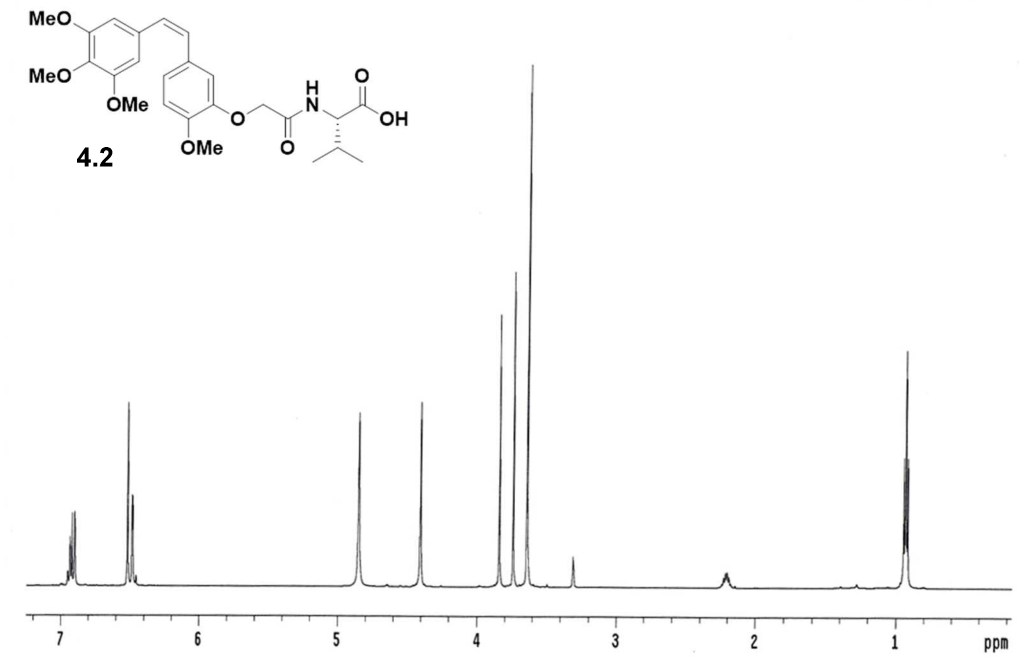
MeO
MeO
OMe
OMe
H
N
O
O
OH
O
4.2
7
6
5
4
3
2
1
ppm

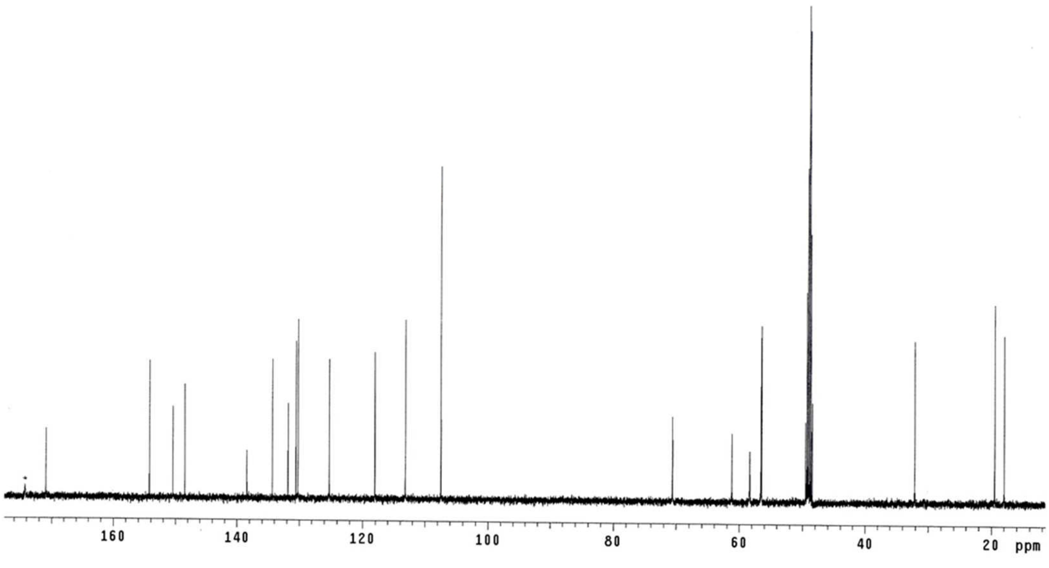
160
140
120
100
80
60
40
20
ppm

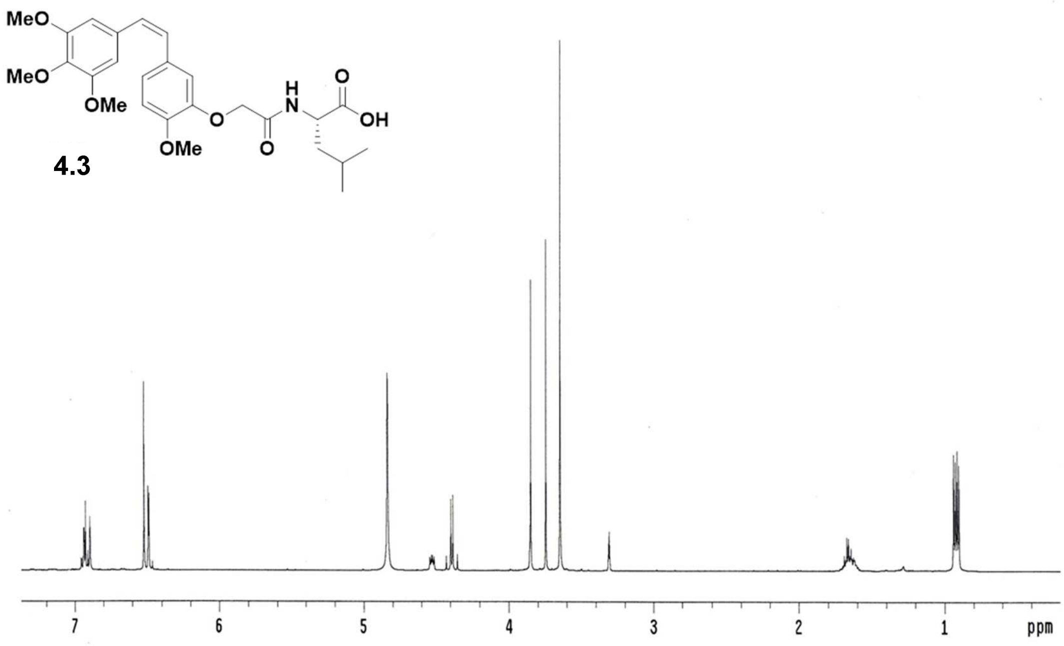
MeO
MeO
OMe
OMe
O
H
N
O
OH
O
4.3
7
6
5
4
3
2
1
ppm

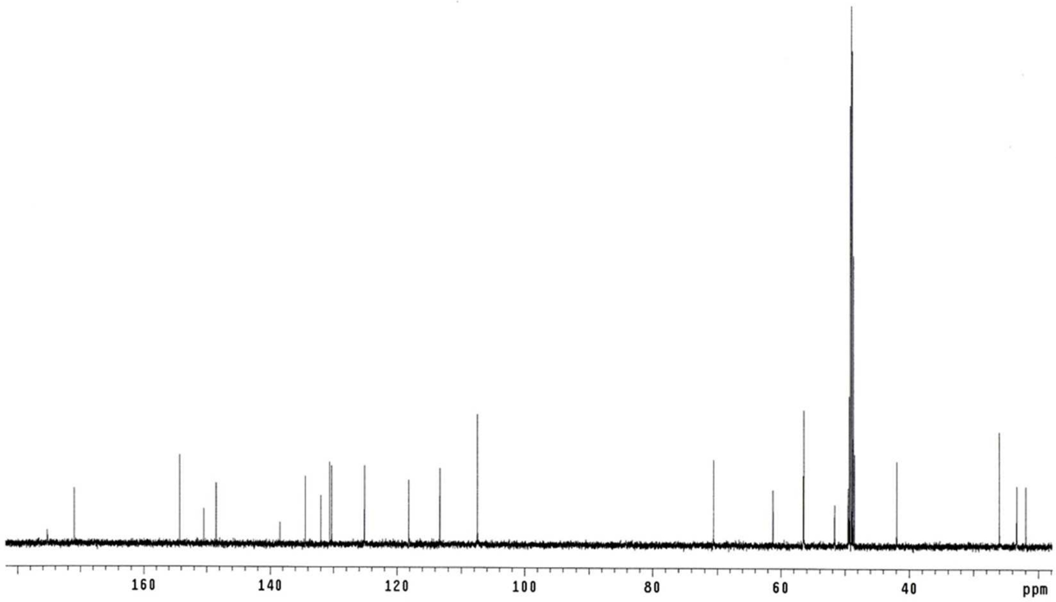
160
140
120
100
80
60
40
ppm

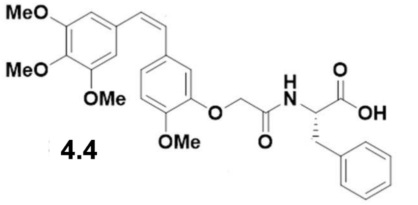
MeO
MeO
OMe
OMe
O
H
N
O
OH
O

4.4

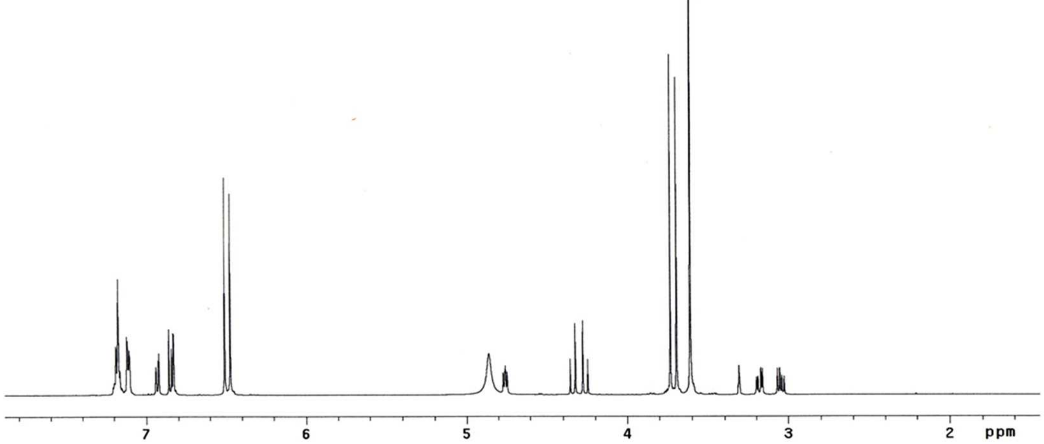
7
6
5
4
3
2
ppm

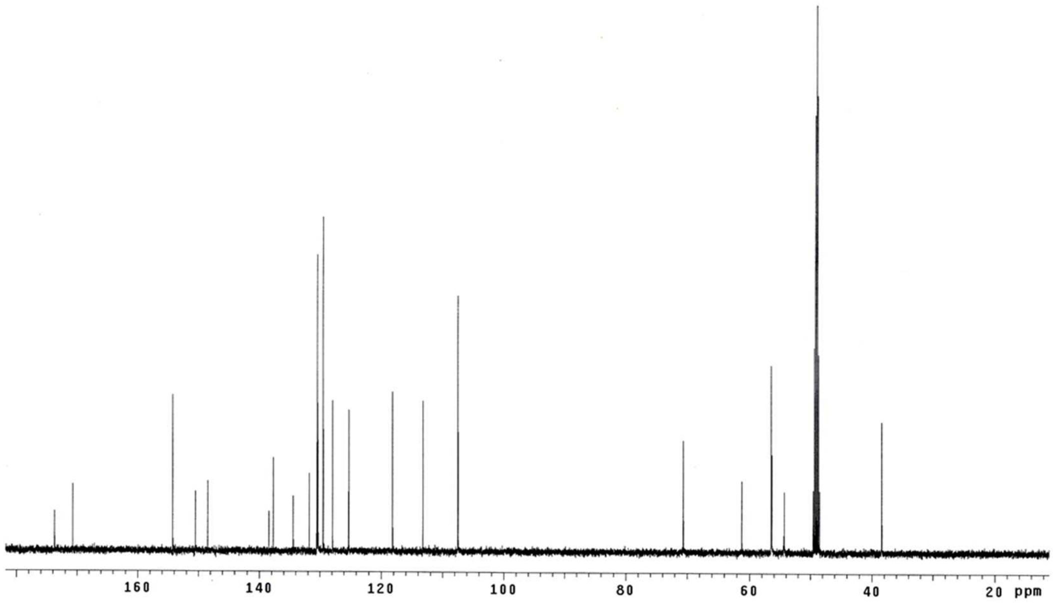
160
140
120
100
80
60
40
20
ppm

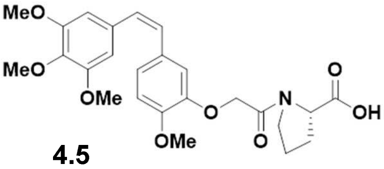
MeO
MeO
OMe
O
OMe
O
N
O
OH
O

4.5

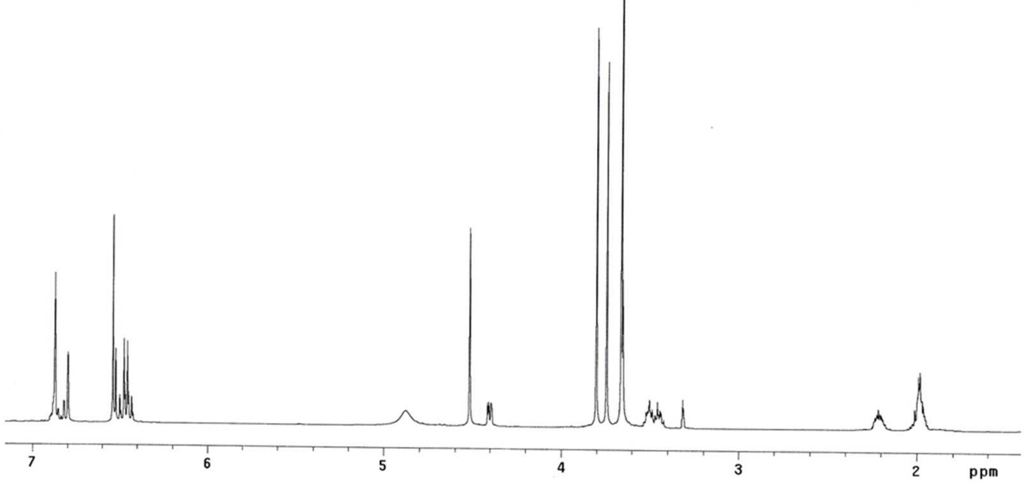
7
6
5
4
3
2
ppm

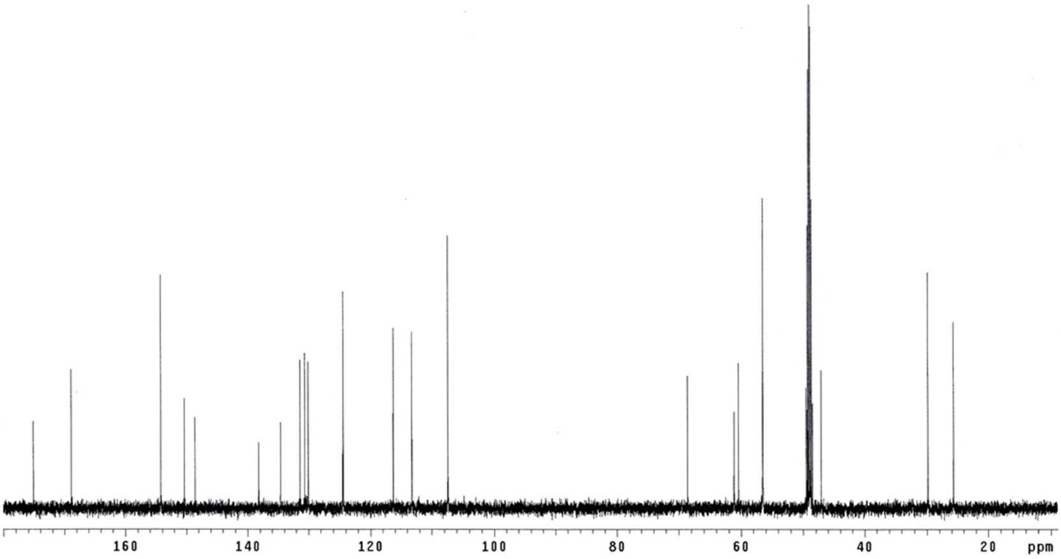
160
140
120
100
80
60
40
20
ppm

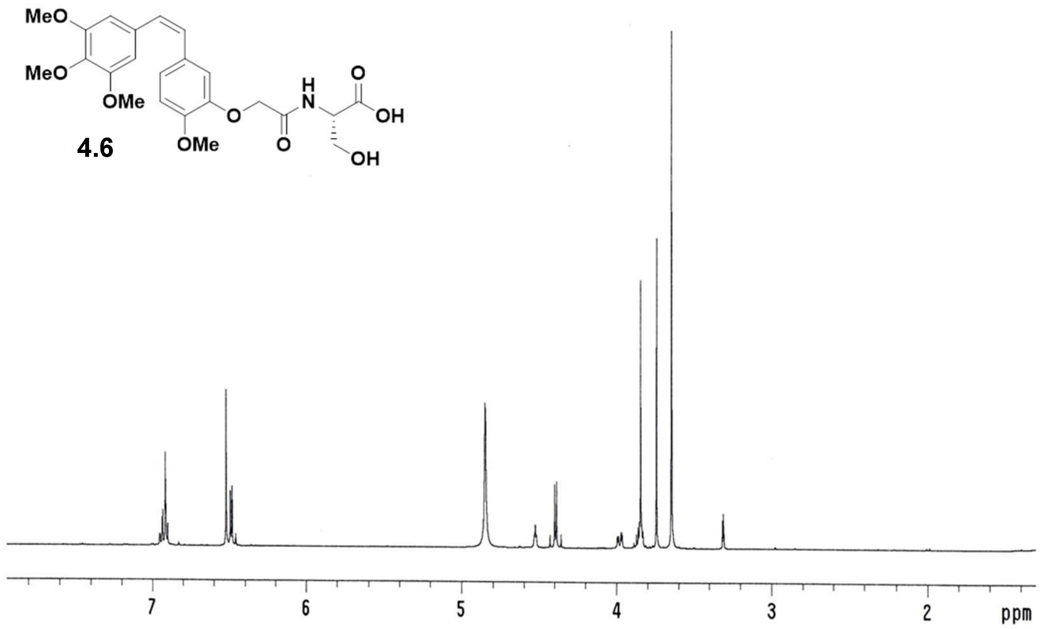
MeO
MeO
OMe
OMe
H
N
O
O
OH
OH
4.6
7
6
5
4
3
2
ppm

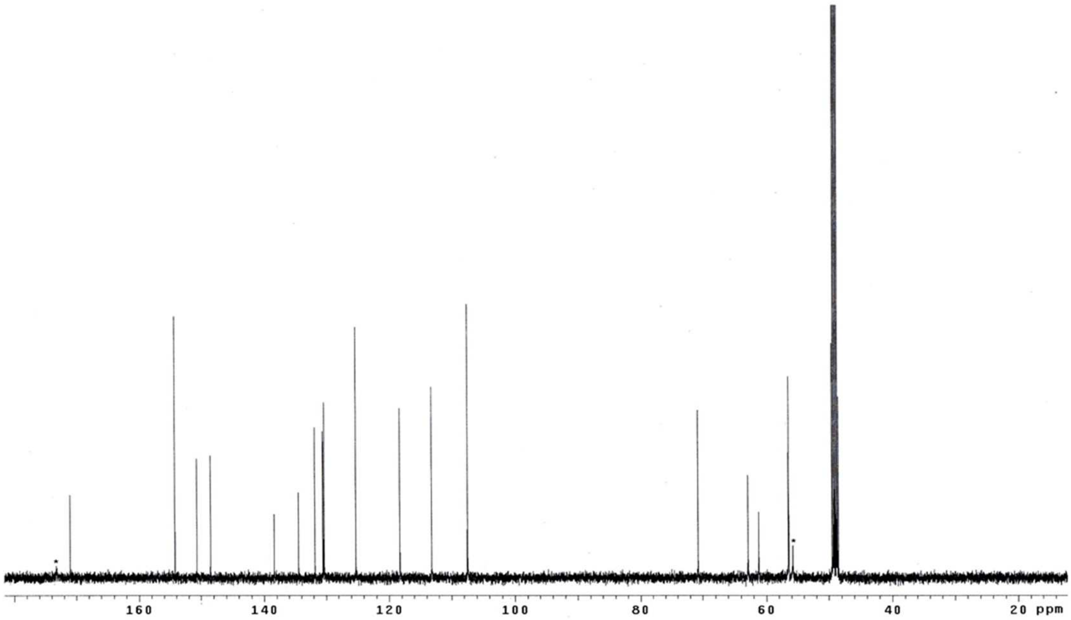
160
140
120
100
80
60
40
20 ppm

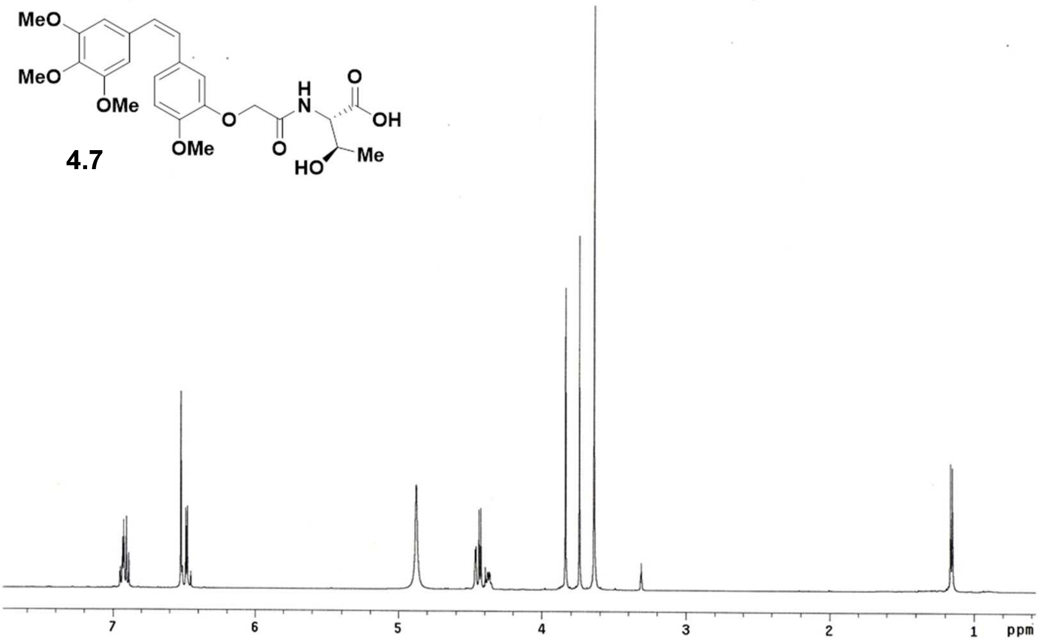
MeO
MeO
OMe
OMe
O
H
N
O
O
OH
HO
Me
4.7
7
6
5
4
3
2
1 ppm

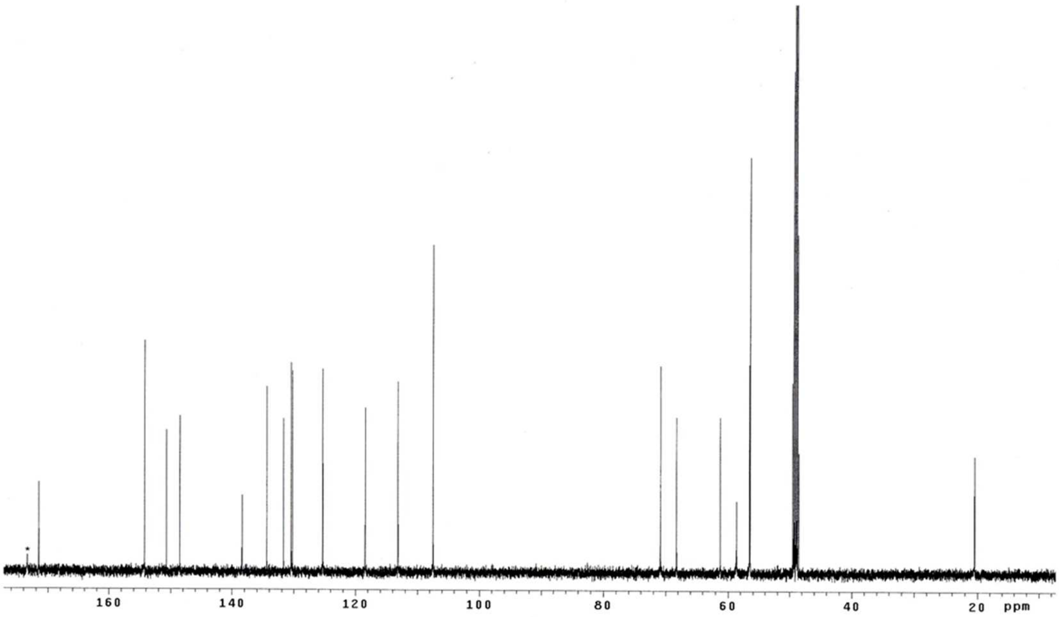
160
140
120
100
80
60
40
20 ppm

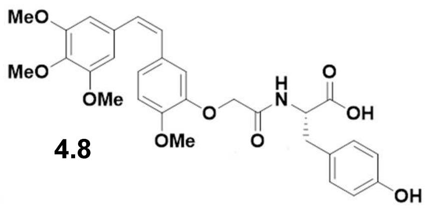
MeO
MeO
OMe
OMe
O
H
N
O
OH
O
OH

4.8

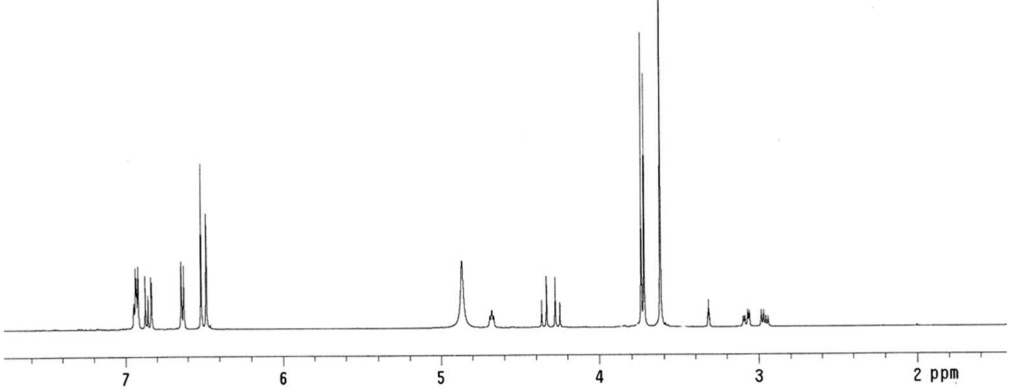
7
6
5
4
3
2 ppm

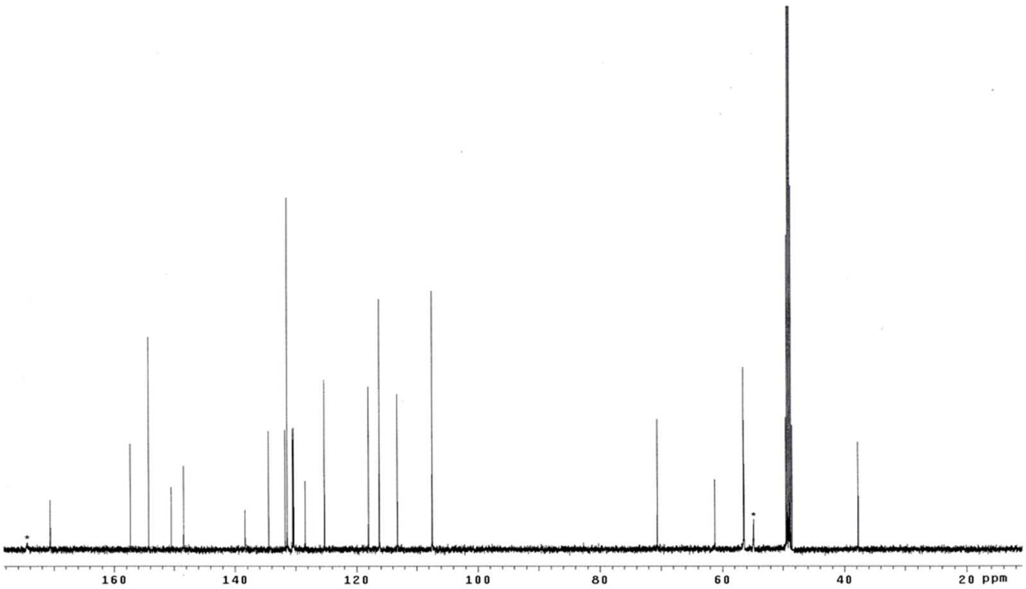
160
140
120
100
80
60
40
20 ppm

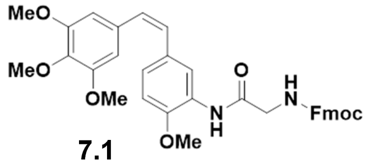
MeO
MeO
OMe
OMe
O
N
H
H
N
Fmoc

7.1

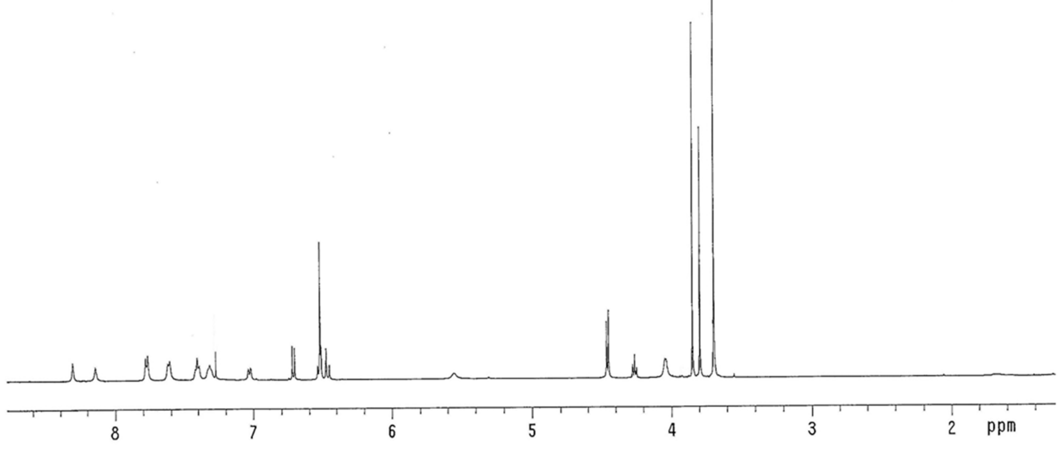
8
7
6
5
4
3
2
ppm

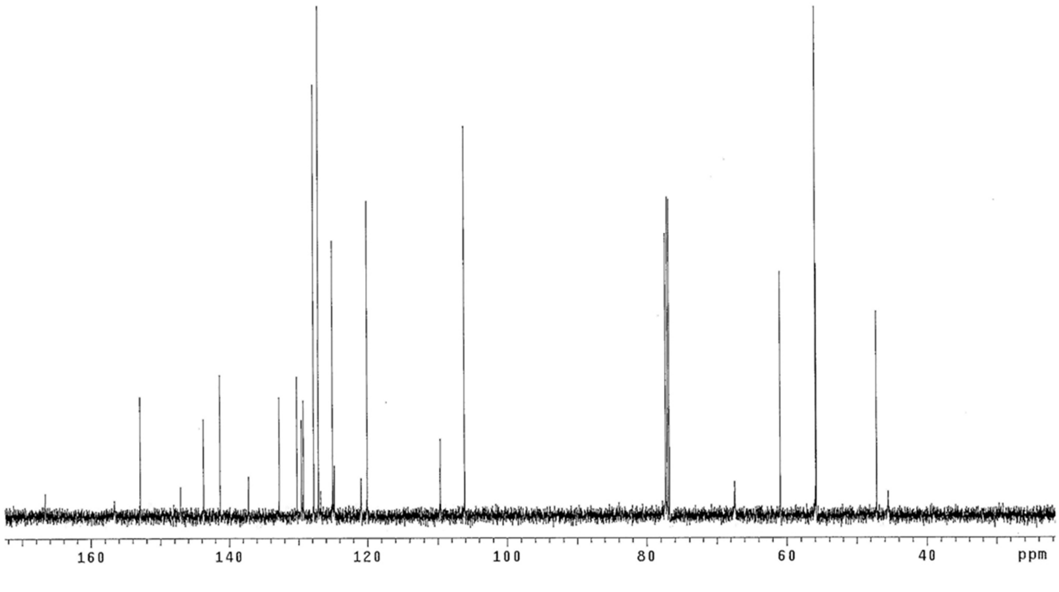
160
140
120
100
80
60
40
ppm

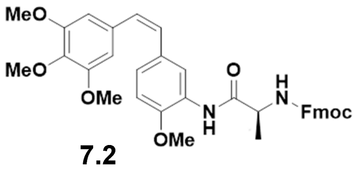
MeO
MeO
OMe
OMe
N
H
O
H
N
Fmoc

7.2

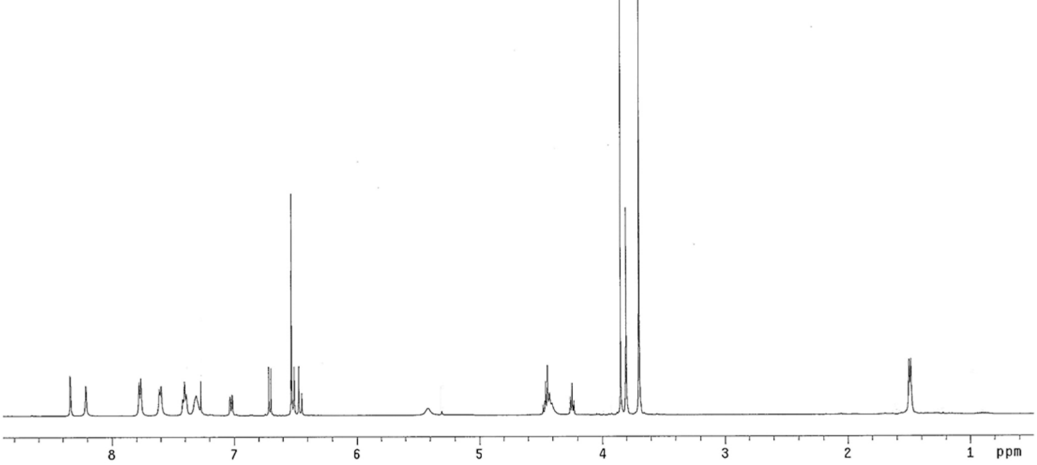
8 7 6 5 4 3 2 1 ppm

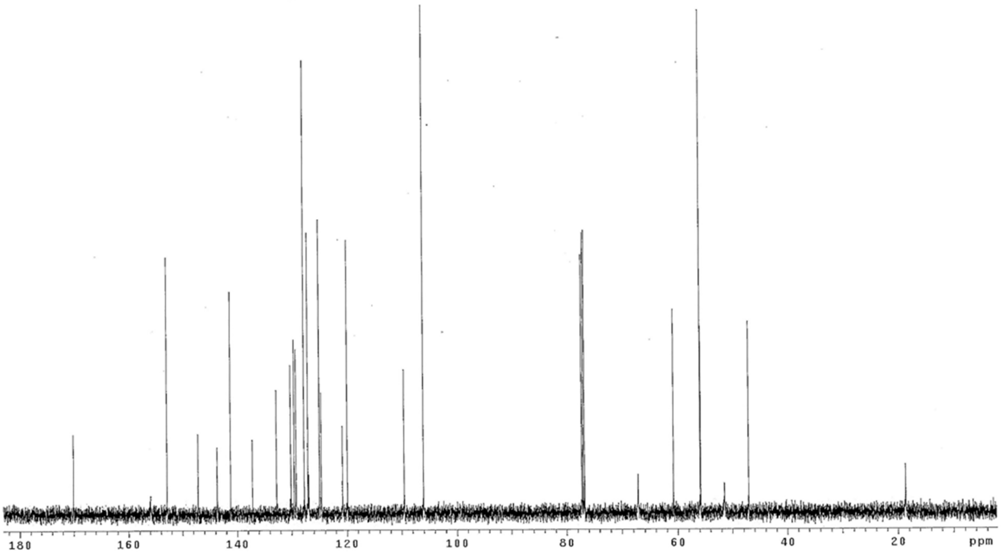
180 160 140 120 100 80 60 40 20 ppm

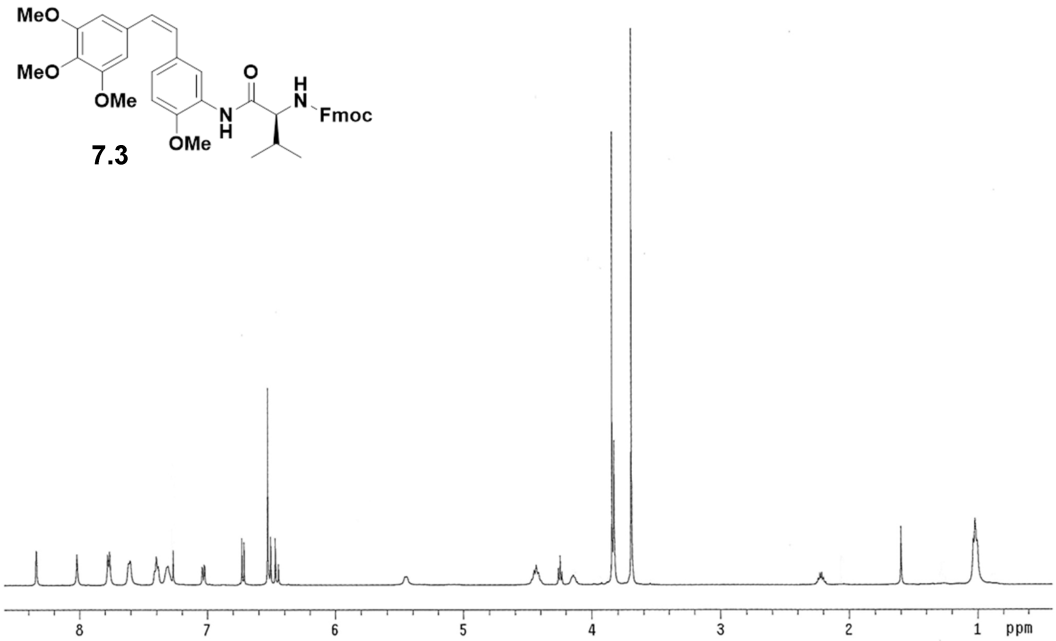
MeO
MeO
OMe
OMe
N
H
O
H
N
Fmoc
7.3
8
7
6
5
4
3
2
1
ppm

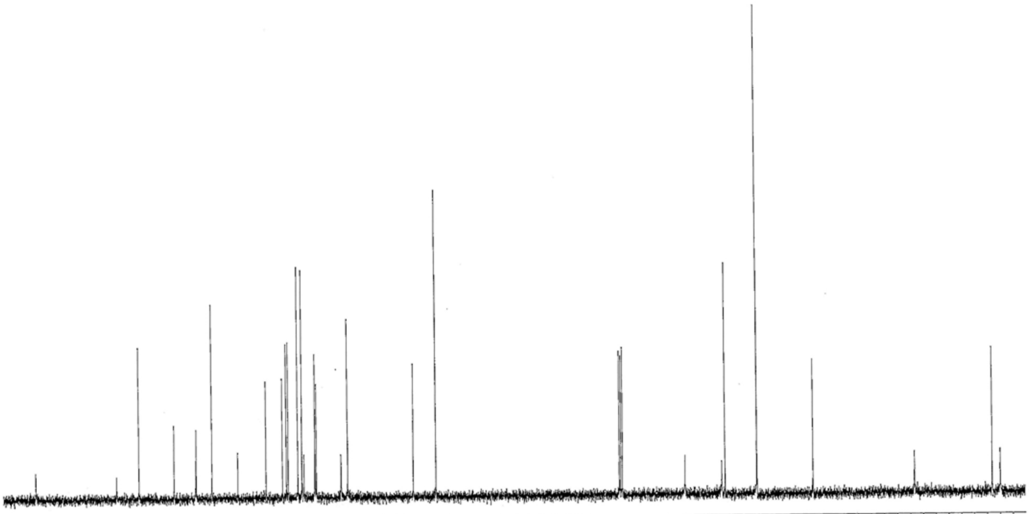
160
140
120
100
80
60
40
ppm

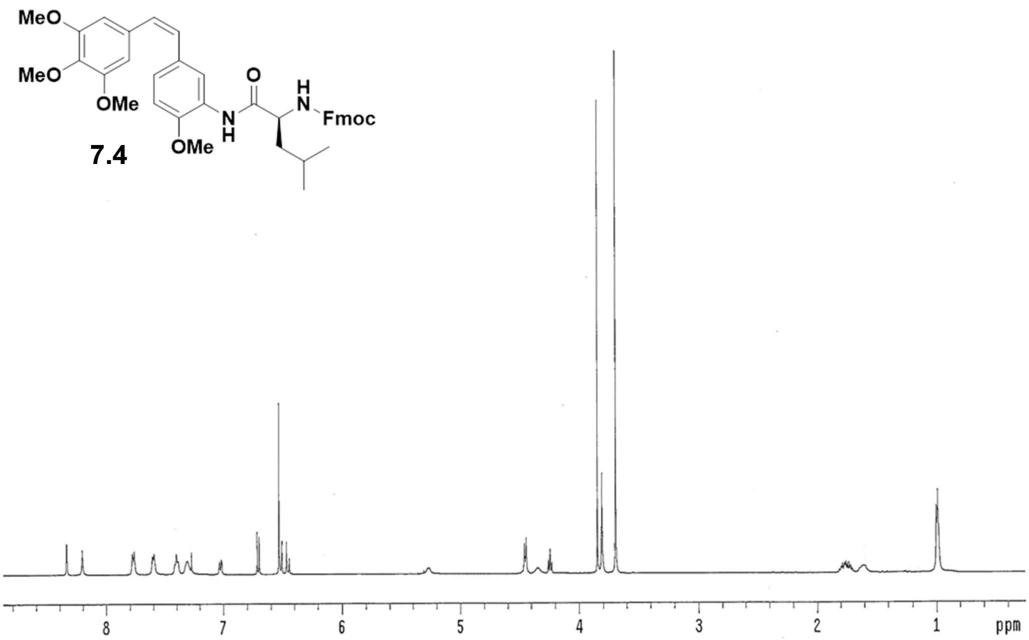
MeO
MeO
OMe
7.4
OMe
N
H
O
H
N
Fmoc
8 7 6 5 4 3 2 1 ppm

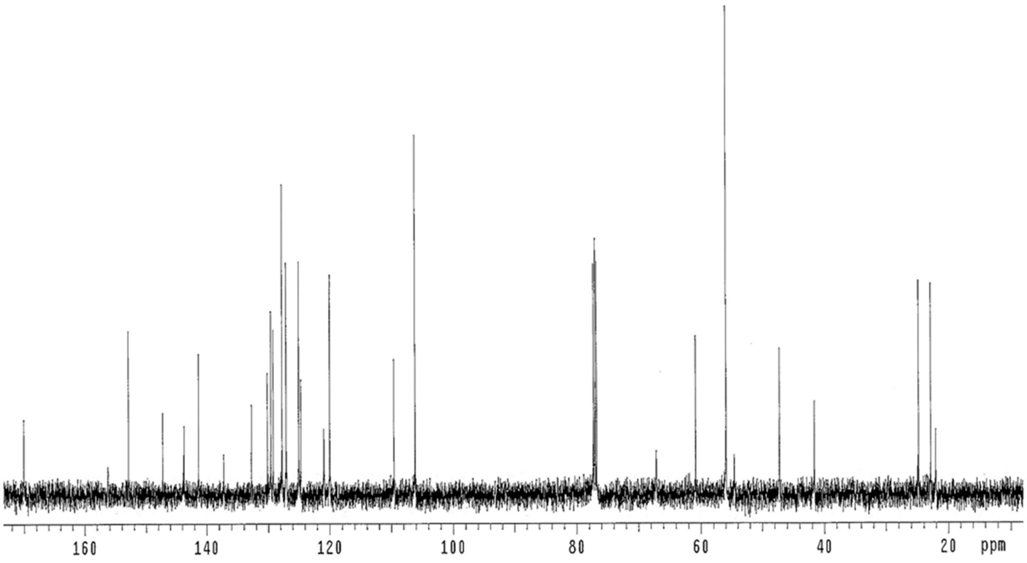
160 140 120 100 80 60 40 20 ppm

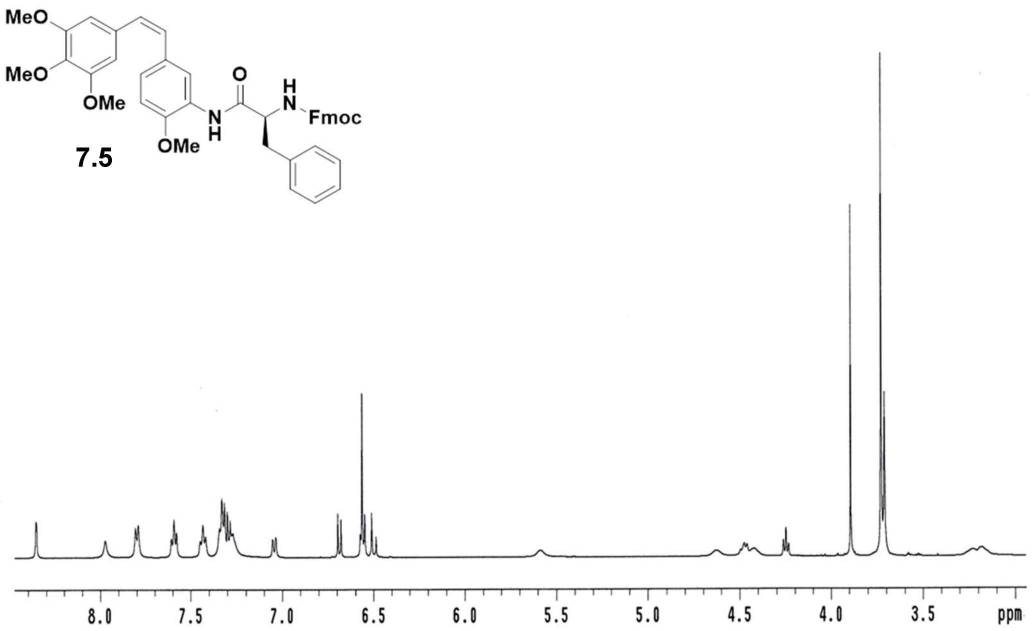
MeO
MeO
OMe
OMe
N
H
O
H
N
Fmoc
7.5
8.0 7.5 7.0 6.5 6.0 5.5 5.0 4.5 4.0 3.5 ppm

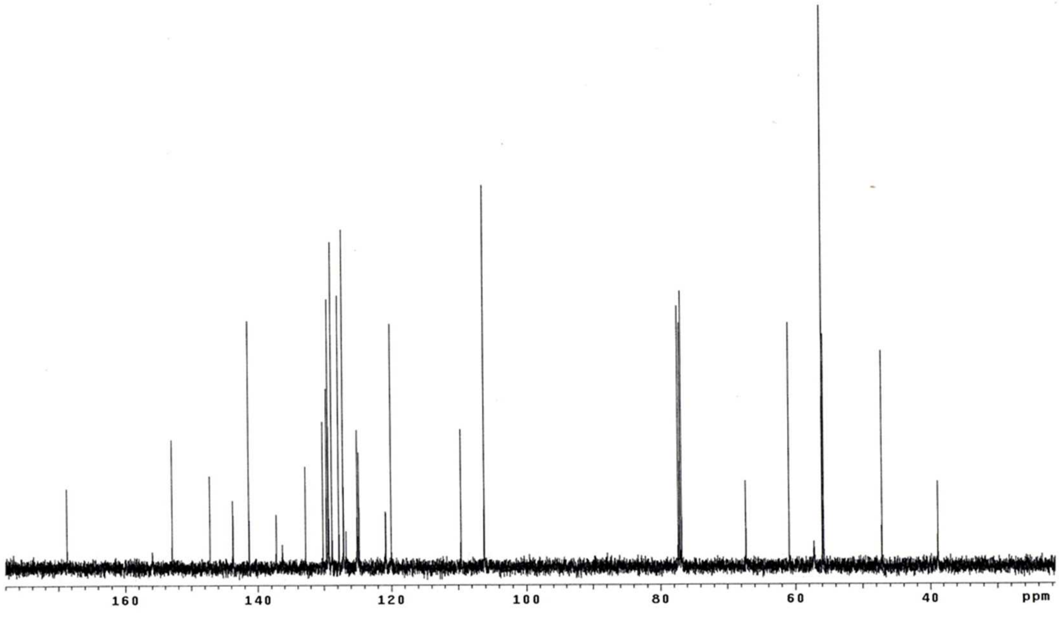
160 140 120 100 80 60 40 ppm

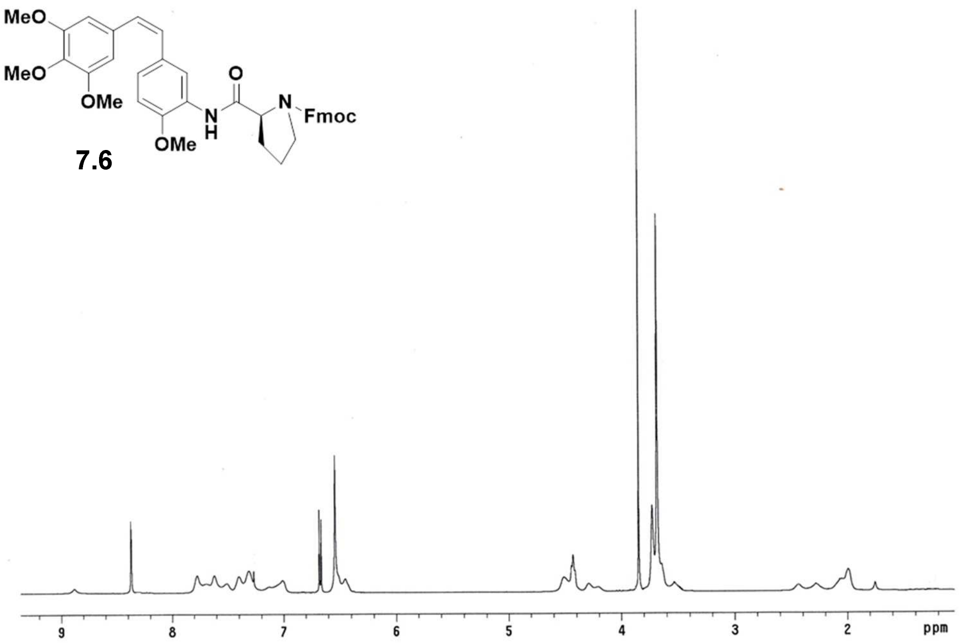
MeO
MeO
OMe
O
N
H
N
Fmoc
OMe
7.6
9
8
7
6
5
4
3
2
ppm

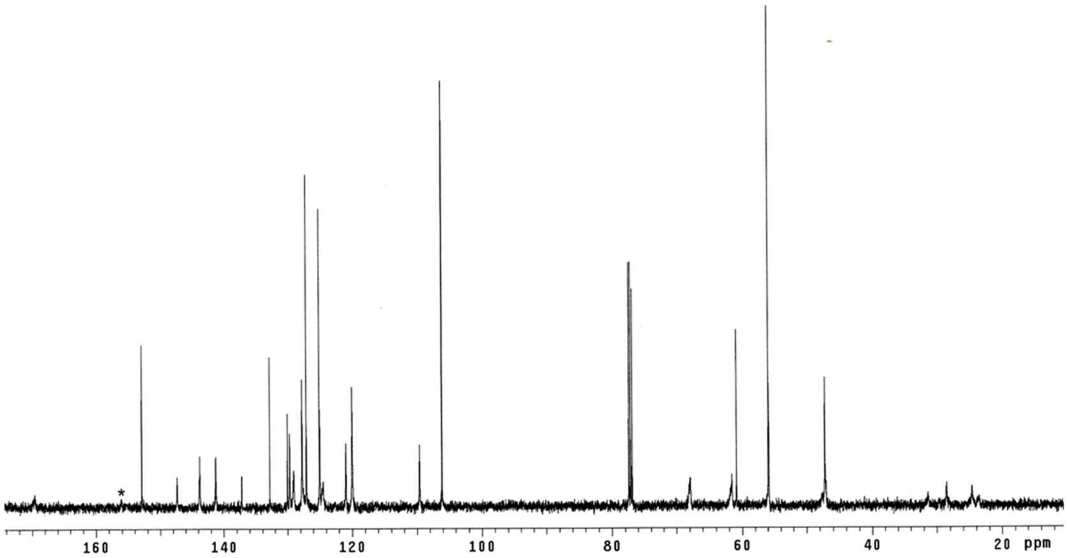
*
160
140
120
100
80
60
40
20
ppm

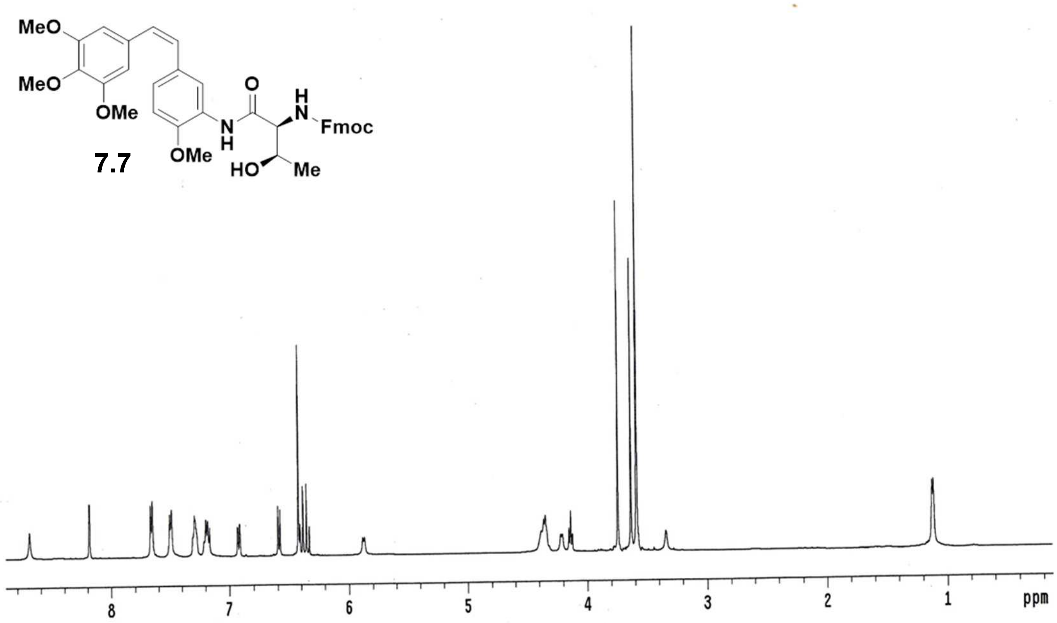
MeO
MeO
OMe
N
H
O
H
N
Fmoc
OMe
HO
Me
7.7
8
7
6
5
4
3
2
1
ppm

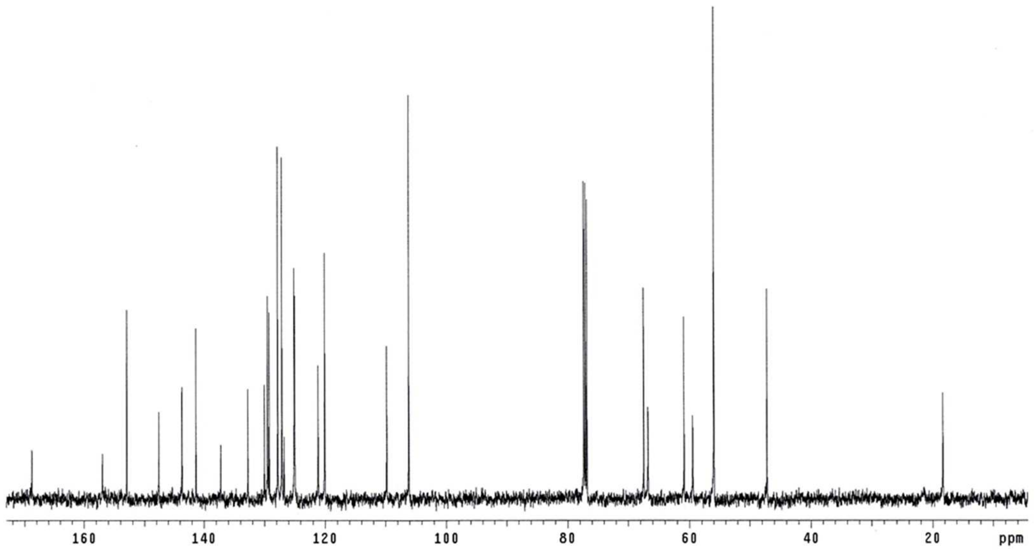
160
140
120
100
80
60
40
20
ppm

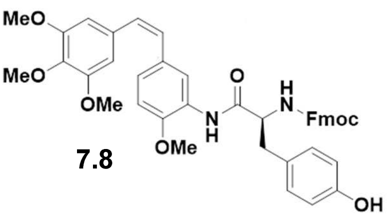
MeO
MeO
OMe
OMe
O
H
N
Fmoc
N
H
OH

7.8

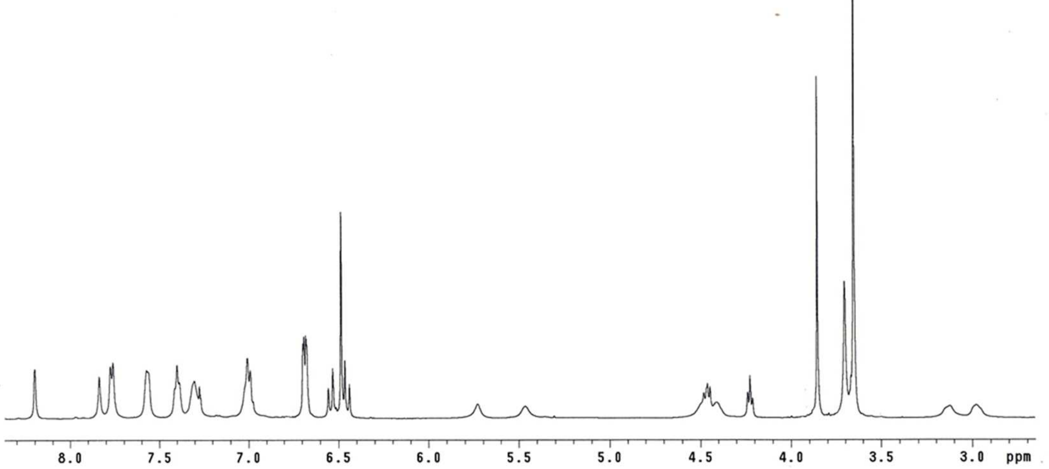
8.0
7.5
7.0
6.5
6.0
5.5
5.0
4.5
4.0
3.5
3.0
ppm

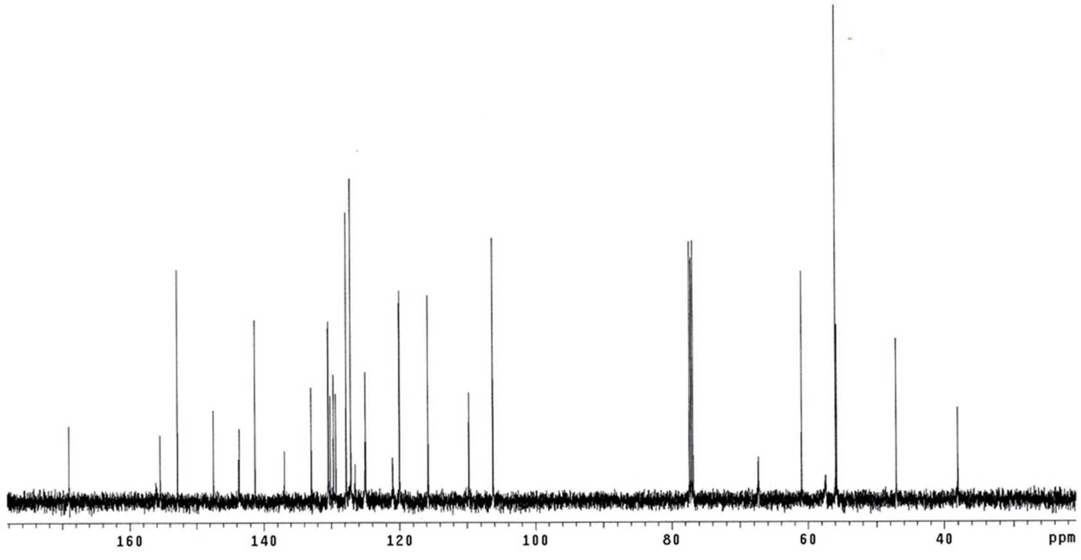
160
140
120
100
80
60
40
ppm

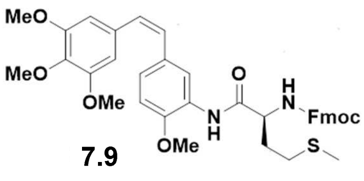
MeO
MeO
OMe
OMe
N
H
O
H
N
Fmoc
S

7.9

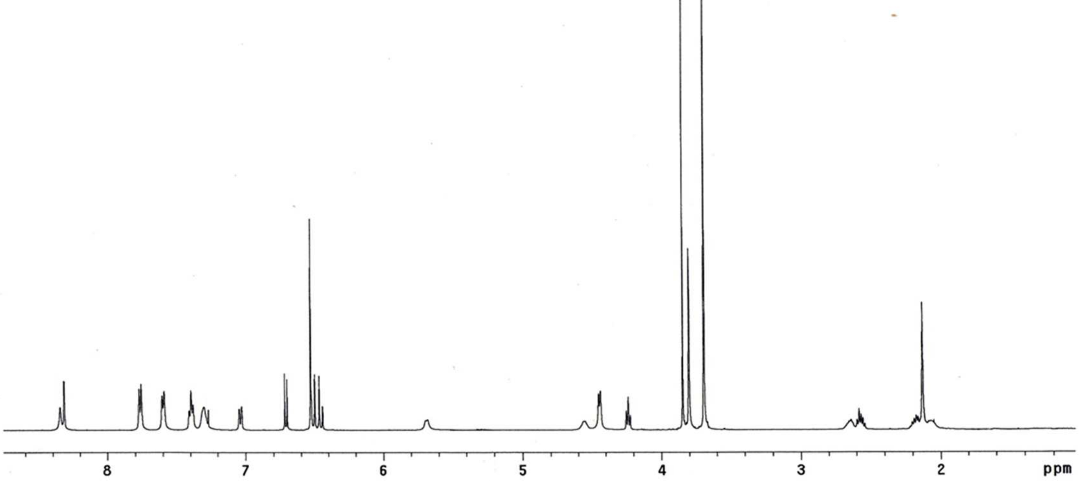
8
7
6
5
4
3
2
ppm

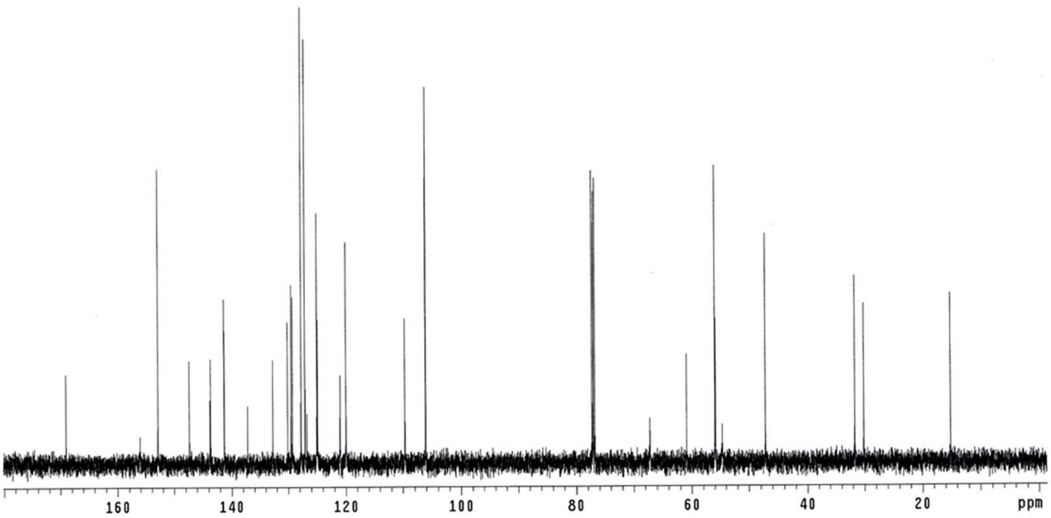
160
140
120
100
80
60
40
20
ppm

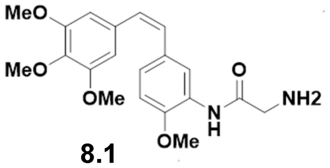
MeO
MeO
OMe
OMe
O
N
H
NH2

8.1

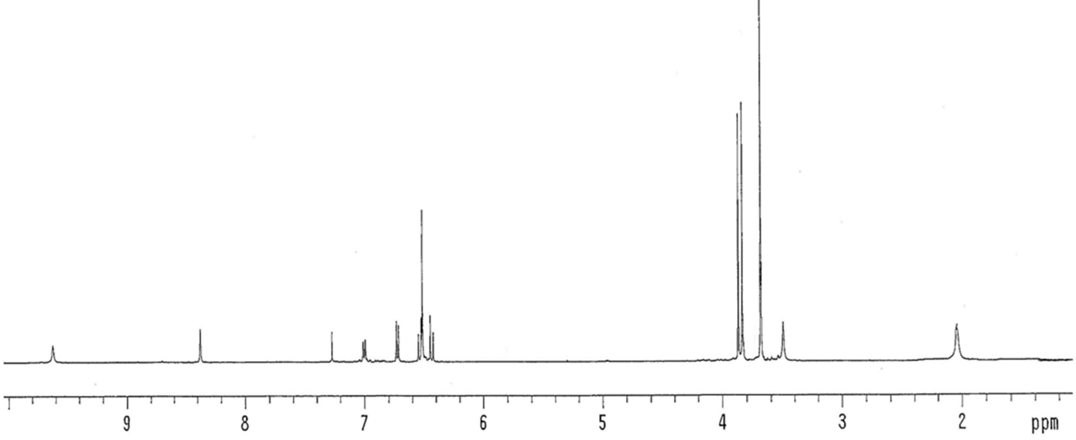
9
8
7
6
5
4
3
2
ppm

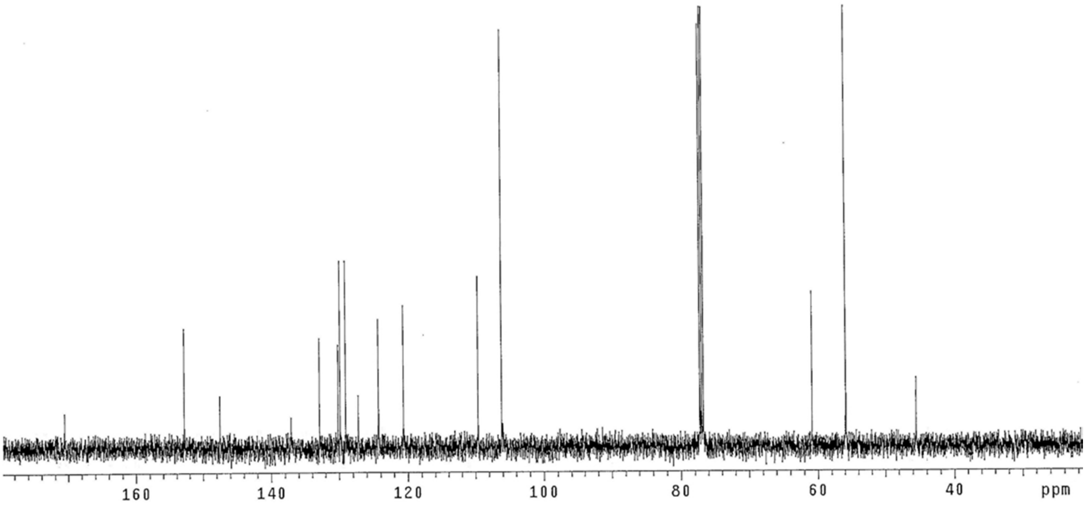
160
140
120
100
80
60
40
ppm

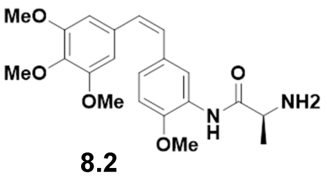
MeO
MeO
OMe
OMe
N
H
O
NH2

8.2

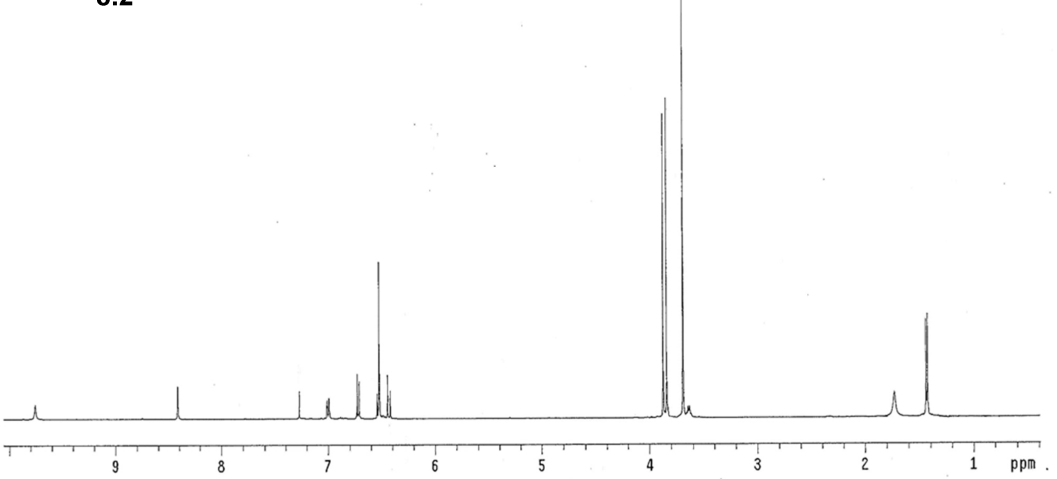
9
8
7
6
5
4
3
2
1
ppm

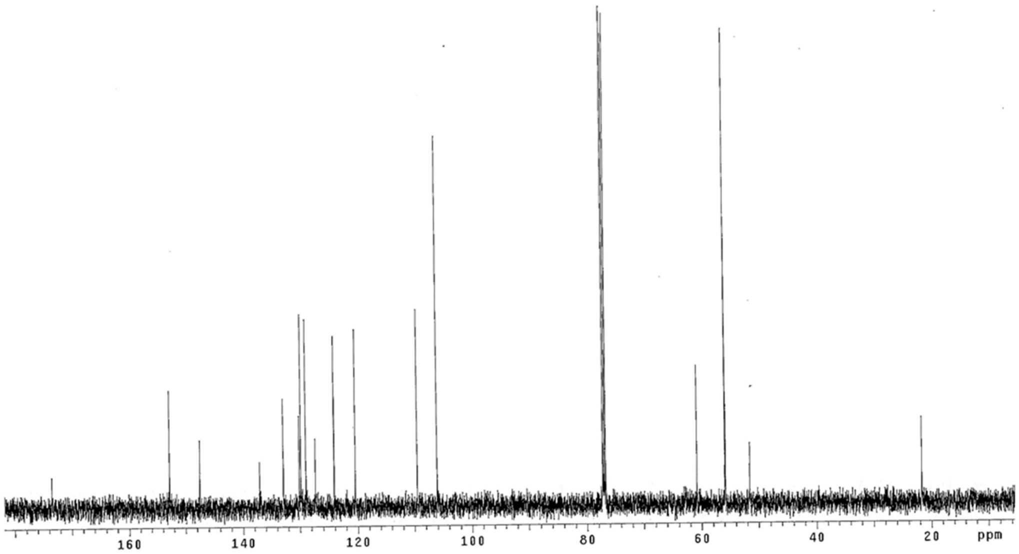
160
140
120
100
80
60
40
20
ppm

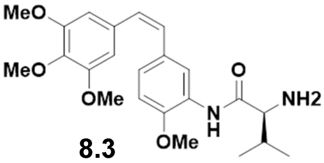
MeO
MeO
OMe
O
NH2
N
H
OMe

8.3

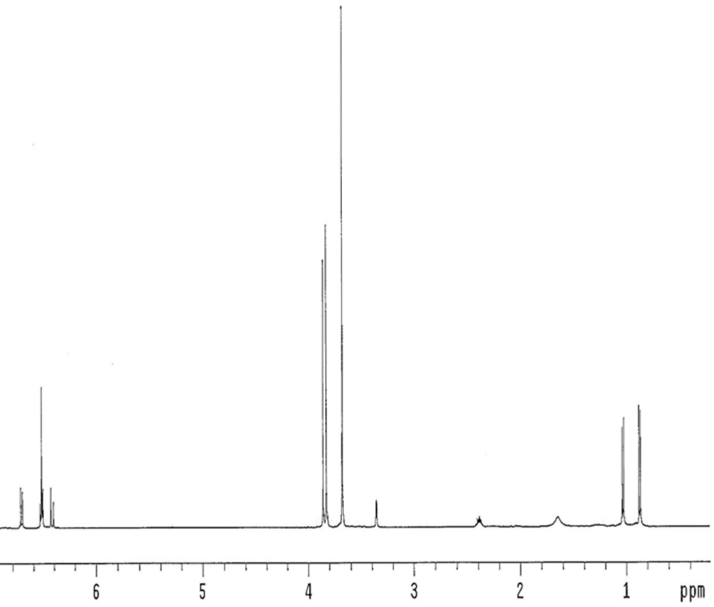
10
9
8
7
6
5
4
3
2
1
ppm

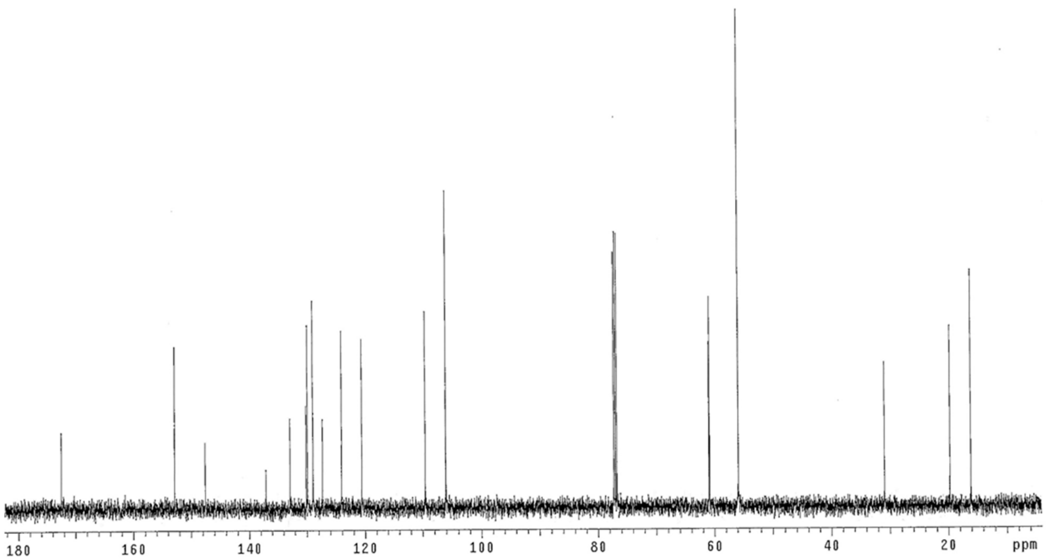
180
160
140
120
100
80
60
40
20
ppm

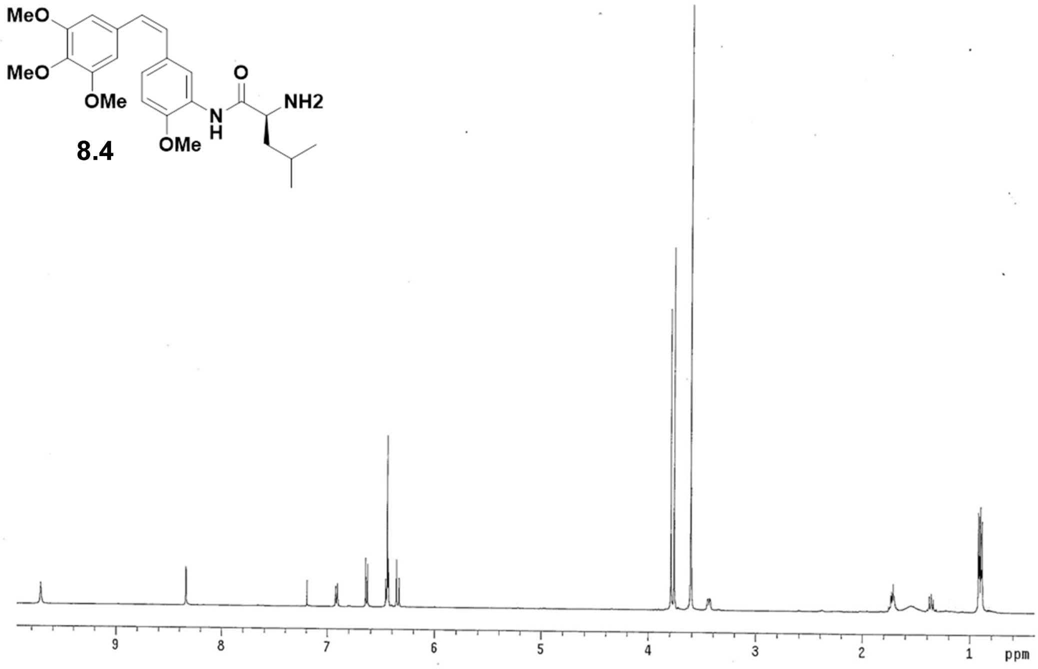
MeO
MeO
OMe
OMe
N
H
O
NH2
9
8
7
6
5
4
3
2
1
ppm

8.4

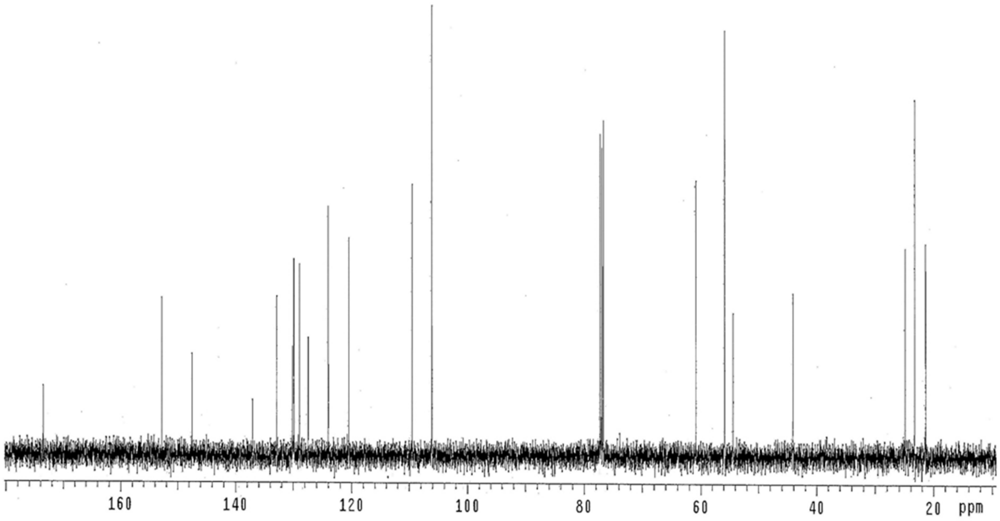
160
140
120
100
80
60
40
20
ppm

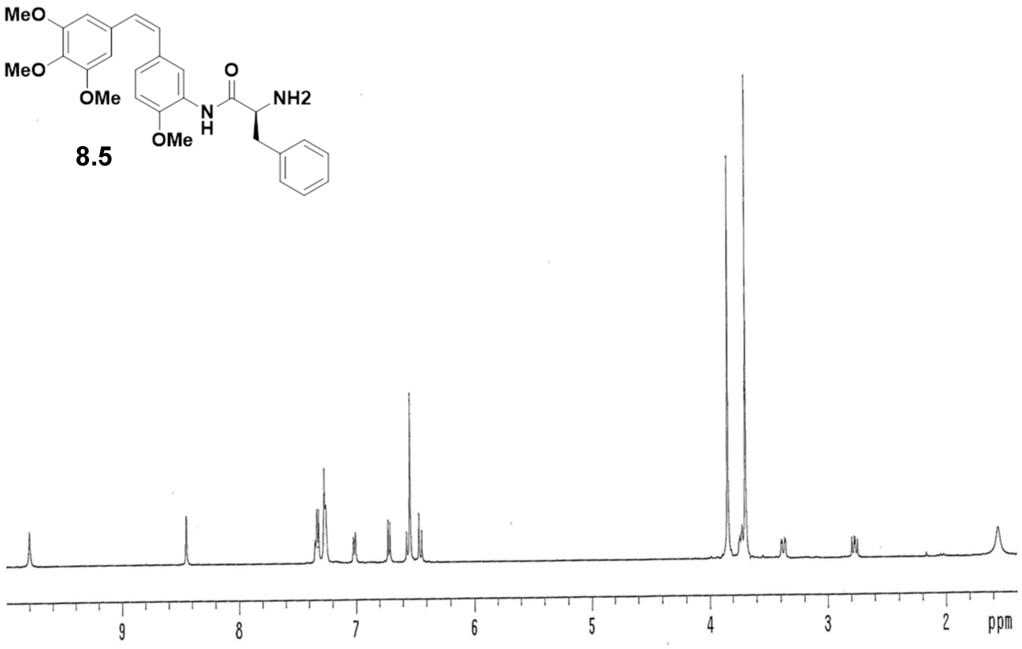
MeO
MeO
OMe
OMe
N
H
O
NH2
8.5
9
8
7
6
5
4
3
2
ppm

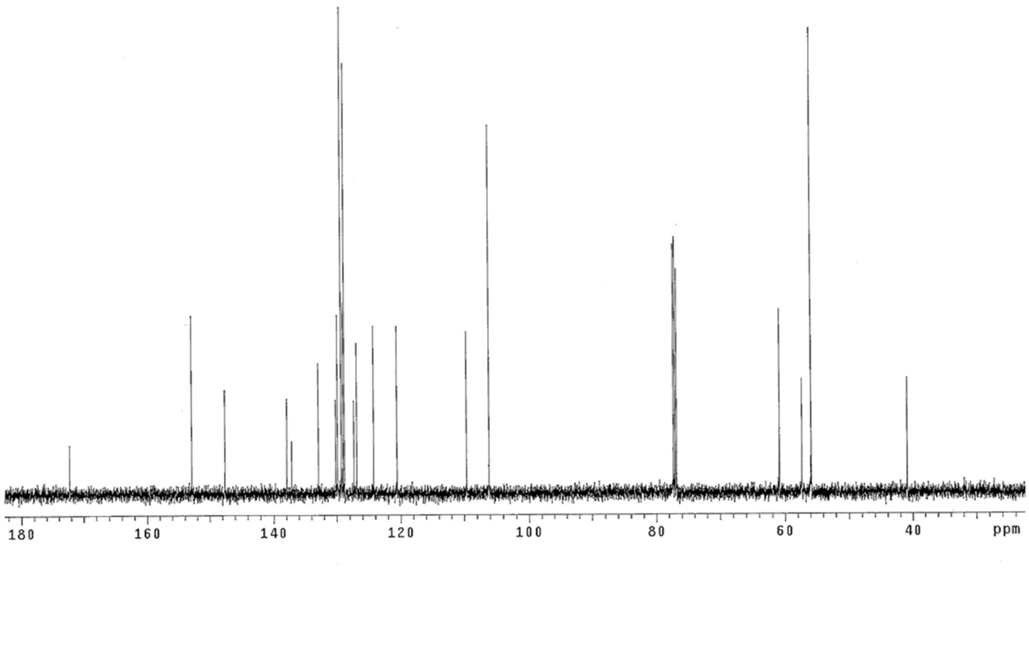
180
160
140
120
100
80
60
40
ppm

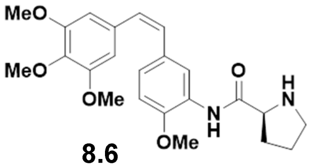
MeO
MeO
OMe
O
H
N
N
H
OMe

8.6

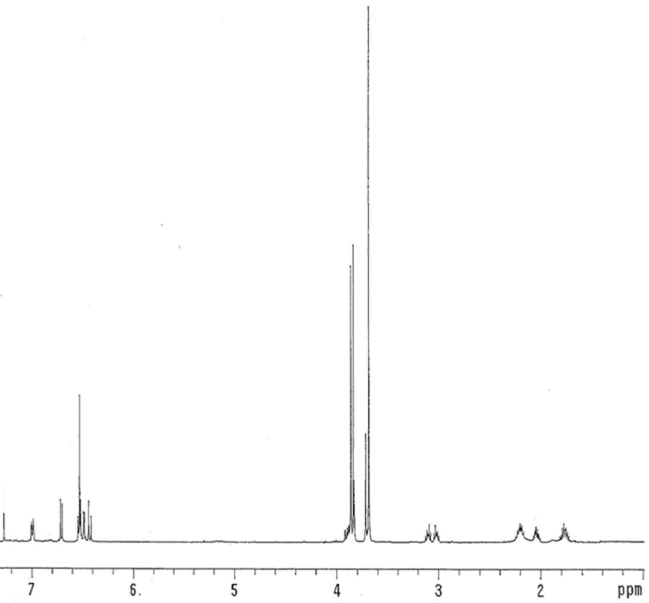
10 9 8 7 6. 5 4 3 2 ppm

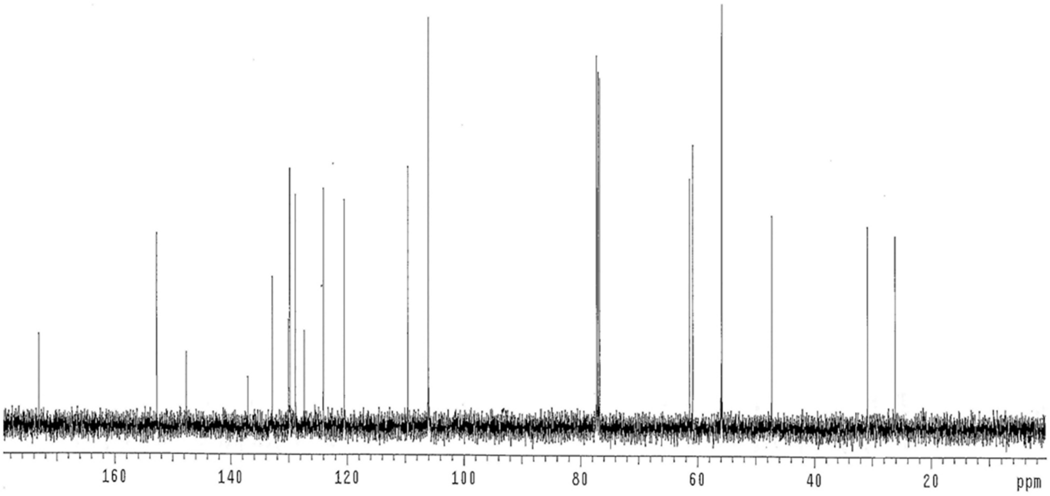
160 140 120 100 80 60 40 20 ppm

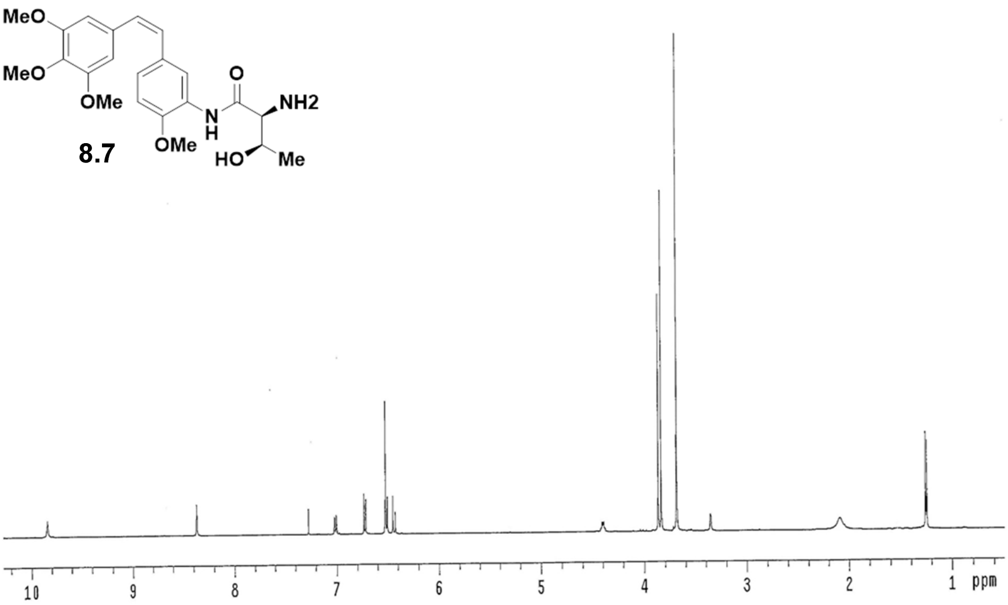
MeO
MeO
OMe
OMe
N
H
O
NH2
HO
Me
8.7
10
9
8
7
6
5
4
3
2
1 ppm

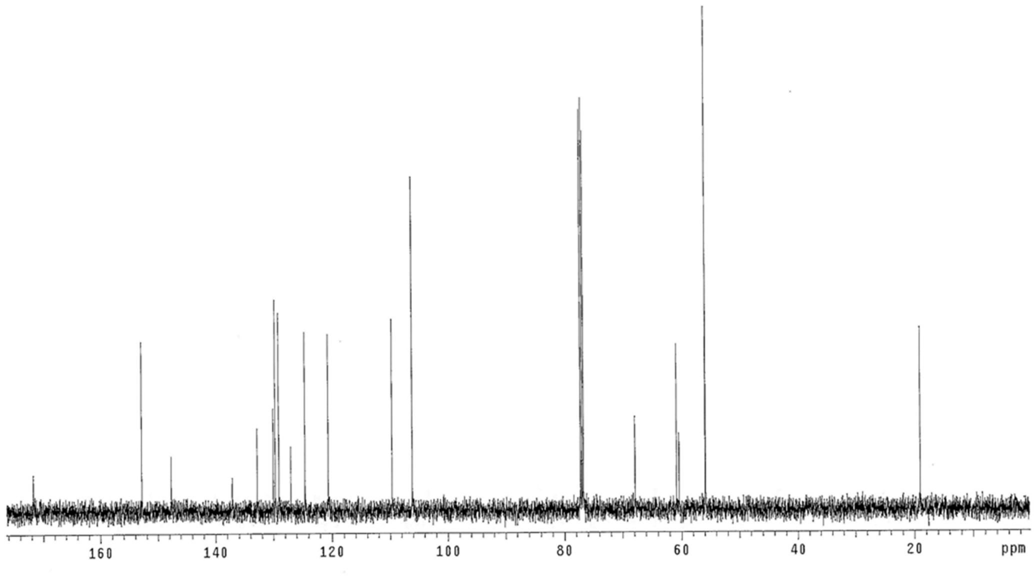
160
140
120
100
80
60
40
20
ppm

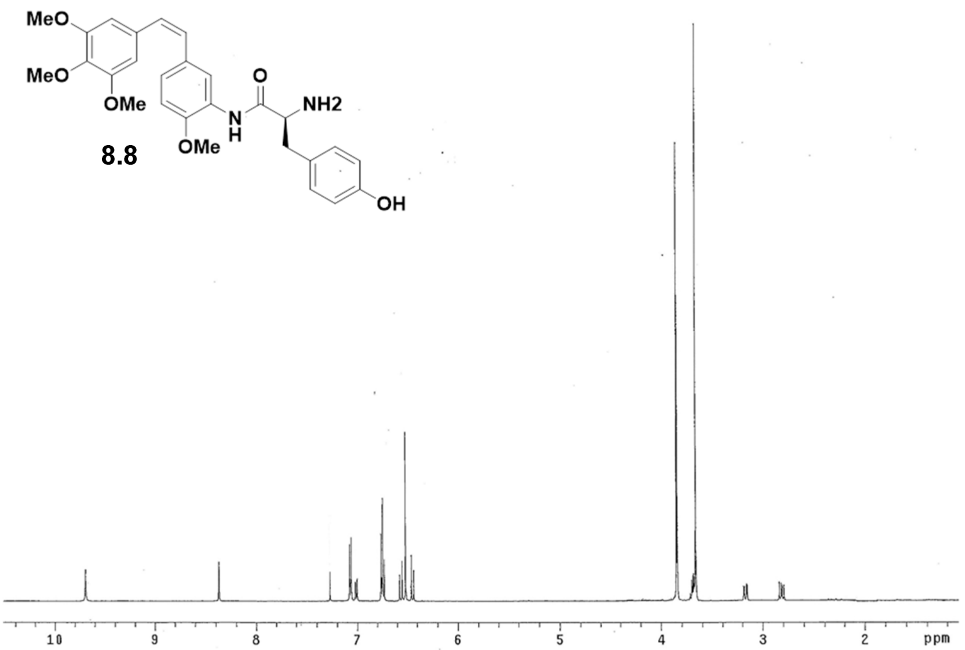
MeO
MeO
OMe
OMe
N
H
O
NH2
OH

8.8

10
9
8
7
6
5
4
3
2
ppm

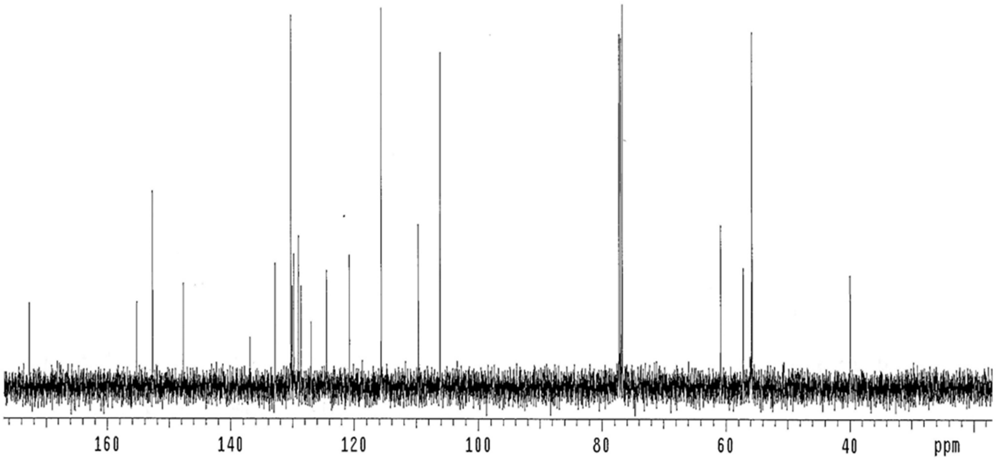
160
140
120
100
80
60
40
ppm

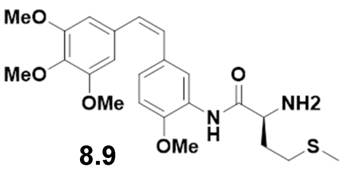
MeO
MeO
OMe
OMe
N
H
O
NH2
S

8.9

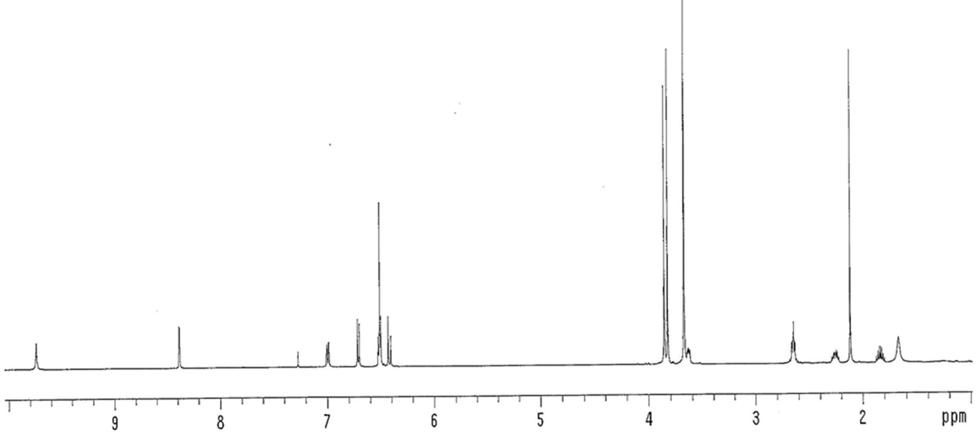
9
8
7
6
5
4
3
2
ppm

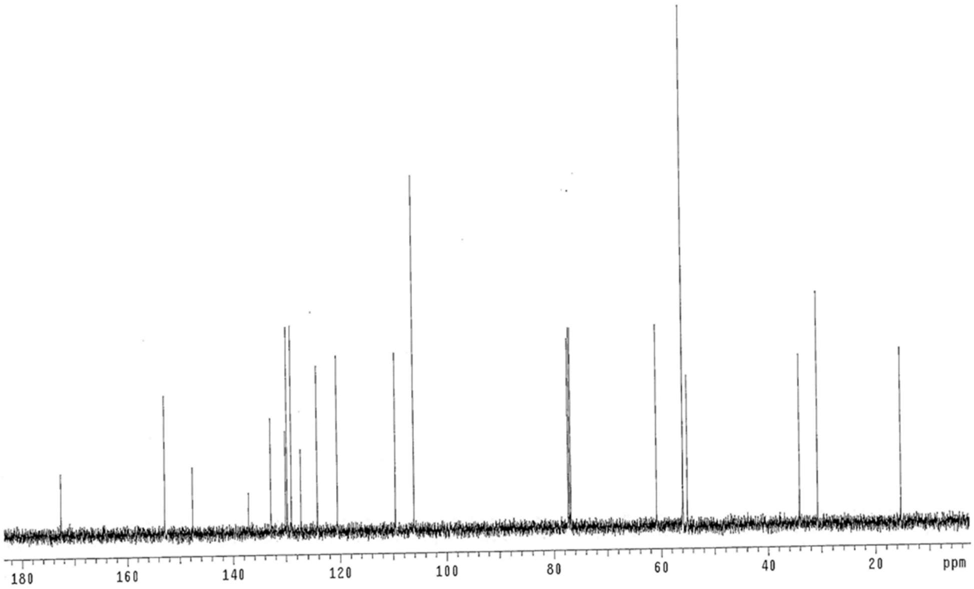
180
160
140
120
100
80
60
40
20
ppm
